# A Y-linked anti-Müllerian hormone type-II receptor is the sex-determining gene in ayu, *Plecoglossus altivelis*

**DOI:** 10.1371/journal.pgen.1009705

**Published:** 2021-08-26

**Authors:** Masatoshi Nakamoto, Tsubasa Uchino, Eriko Koshimizu, Yudai Kuchiishi, Ryota Sekiguchi, Liu Wang, Ryusuke Sudo, Masato Endo, Yann Guiguen, Manfred Schartl, John H. Postlethwait, Takashi Sakamoto

**Affiliations:** 1 Department of Marine Biosciences, Tokyo University of Marine Science and Technology, Tokyo, Japan; 2 Department of Human Genetics, Yokohama City University, Graduate School of Medicine, Yokohama, Japan; 3 INRAE, LPGP, Rennes, France; 4 University of Wuerzburg, Developmental Biochemistry, Biocenter, Würzburg, Germany; 5 The Xiphophorus Genetic Stock Center, Department of Chemistry and Biochemistry, Texas State University, San Marcos, Texas, United States of America; 6 Institute of Neuroscience, University of Oregon, Eugene, Oregon, United States of America; Fred Hutchinson Cancer Research Center, UNITED STATES

## Abstract

Whole-genome duplication and genome compaction are thought to have played important roles in teleost fish evolution. Ayu (or sweetfish), *Plecoglossus altivelis*, belongs to the superorder Stomiati, order Osmeriformes. Stomiati is phylogenetically classified as sister taxa of Neoteleostei. Thus, ayu holds an important position in the fish tree of life. Although ayu is economically important for the food industry and recreational fishing in Japan, few genomic resources are available for this species. To address this problem, we produced a draft genome sequence of ayu by whole-genome shotgun sequencing and constructed linkage maps using a genotyping-by-sequencing approach. Syntenic analyses of ayu and other teleost fish provided information about chromosomal rearrangements during the divergence of Stomiati, Protacanthopterygii and Neoteleostei. The size of the ayu genome indicates that genome compaction occurred after the divergence of the family Osmeridae. Ayu has an XX/XY sex-determination system for which we identified sex-associated loci by a genome-wide association study by genotyping-by-sequencing and whole-genome resequencing using wild populations. Genome-wide association mapping using wild ayu populations revealed three sex-linked scaffolds (total, 2.03 Mb). Comparison of whole-genome resequencing mapping coverage between males and females identified male-specific regions in sex-linked scaffolds. A duplicate copy of the anti-Müllerian hormone type-II receptor gene (*amhr2bY*) was found within these male-specific regions, distinct from the autosomal copy of *amhr2*. Expression of the Y-linked *amhr2* gene was male-specific in *sox9b*-positive somatic cells surrounding germ cells in undifferentiated gonads, whereas autosomal *amhr2* transcripts were detected in somatic cells in sexually undifferentiated gonads of both genetic males and females. Loss-of-function mutation for *amhr2bY* induced male to female sex reversal. Taken together with the known role of *Amh* and *Amhr2* in sex differentiation, these results indicate that the paralog of *amhr2* on the ayu Y chromosome determines genetic sex, and the male-specific amh-amhr2 pathway is critical for testicular differentiation in ayu.

## Author summary

Ayu (or sweetfish), *Plecoglossus altivelis*, is widely distributed in East Asia. Ayu belongs to the superorder Stomiati and the order Osmeriformes. Stomiati is phylogenetically classified as sister group of Neoteleostei, the largest clade of bony fish including medaka, tuna and cod. The divergence of Protacanthopterygii (salmon and pike) and the common ancestor of Stomiati and Neoteleostei is estimated to have occurred approximately 190 million years ago. Thus, ayu holds an important position in the fish tree of life. We sequenced the ayu genome and constructed linkage maps using a genotyping-by-sequencing approach. Comparative analyses of ayu, medaka and northern pike revealed chromosomal rearrangements in the ayu lineage after the divergence of ayu and northern pike. Association mapping revealed a duplicate copy of the anti-Müllerian hormone type-II receptor gene (*amhr2bY*) located within a male-specific region. Y-linked *amhr2* expression was male-specific in supporting cells in undifferentiated gonads, whereas autosomal *amhr2* transcripts were detected in somatic cells of sexually undifferentiated gonads in both. Loss-of-function mutation for *amhr2bY* induced male-to-female sex reversal. Taken together, these results indicate that the paralog of *amhr2* on the Y chromosome determines genetic sex. Our findings support the hypothesis that the male-specific amh-amhr2 pathway is critical for gonadal differentiation in ayu.

## Introduction

Gonadal sex determination, the process that initiates the development of the testis or ovary from the gonadal primordium, is a universal feature in vertebrate development. The convergence of gonad morphology and gene function during gonadal sex differentiation among vertebrates indicates that there is a basic system derived from a common ancestor that has been conserved throughout vertebrate evolution [1,2]. In contrast, sex-determining genes are highly diverse among vertebrates, especially among teleost fishes [3]. For example, *dmrt1bY/dmy*, *gsdfY*, and *sox3* have all been identified as sex-determining genes in *Oryzias* fishes [4–7]; and the immune-related gene *sdY* determines sex in salmonids [8]. In *Seriola* fishes, sex is most likely determined by female-specific synthesis of estrogens by hsd17b1, encoded by a gene located on the W chromosome [9]. The Y-linked anti-Müllerian hormone gene (*amh*) is the sex-determining gene in Patagonian pejerrey, northern pike and threespine stickleback [10–12], and a missense SNP in the anti-Müllerian hormone type-II receptor gene (*amhr2*) is critical for sex determination in tiger pufferfish [13]. In yellow perch, the Y-linked duplicate of *amhr2* is a strong candidate for the sex-determining gene [14]. In general, sex-determination genes seem to evolve frequently from genes that function in gonadal sex differentiation pathways, although there are exceptions, like in salmonids [11].

Ayu (or sweetfish), *Plecoglossus altivelis*, is widely distributed in East Asia, especially in Japan, Korea, Taiwan and China [15–17]. Ayu belongs to the order Osmeriformes and the family Osmeridae. This fish has a 1-year lifespan and a diadromous migration status, i.e. it migrates between fresh water and the sea. There are some similarities in life cycle and morphology between ayu and salmon. Previously, Osmeriformes, the order to which ayu belongs, was phylogenetically classified into the superorder Protacanthopterygii, which contains salmon and pike [18]. More recently, however, Osmeriformes has been phylogenetically classified into the superorder Stomiati [19–22], which is sister taxa of Neoteleostei (S1 Fig). The divergence of Protacanthopterygii and the common ancestor of Stomiati and Neoteleostei is estimated to have occurred approximately 190 million years ago, and the divergence of Stomiati and Neoteleostei is estimated to have occurred approximately 180 million years ago [19,21,22]. The common ancestor of Osmeridae and Salmonidae diverged before the salmonid-specific whole-genome duplication (SaGD) event. Thus, ayu holds an important position in the fish tree of life. Comparative whole-genome analyses of ayu and other teleost fish can help to reveal aspects of chromosomal evolution in teleost fish.

Ayu are economically important for the food industry and recreational fishing in Japan. Aquaculture production of ayu was approximately 5,000 tons in 2017, the second largest inland aquaculture production in Japan [23]. In Japan, female ayu are more valuable than males as food, because mature fish with eggs are considered delicious. Thus, a genetic method to identify sex early in ayu development would have economic significance. In ayu, genetic sex is determined by an XX/XY system, and sex-linked AFLP markers have been developed [24–26]. However, little is known about the genomic region at the sex-determining locus in ayu.

In this study, we constructed a reference genome sequence for ayu using a whole-genome shotgun sequencing method from a one male individual and constructed genetic linkage maps using genotyping-by-sequencing (GBS)-derived single nucleotide polymorphisms (SNPs). The ayu genome sequence was analyzed to determine syntenic relationships with other fishes and to provide new insights into teleost genome evolution. We also identified sex-associated loci by genome-wide association studies using wild ayu populations. A strong candidate for the ayu sex-determining gene, a paralog of *amhr2*, was found to be expressed in a male-specific manner in supporting cells surrounding germ cells in morphologically undifferentiated gonads. These findings provide genetic evidence that the paralog of *amhr2* on the Y chromosome is the sex-determining gene of ayu. We provide the first report for the sex-determining gene of species belong to the superorder Stomiati.

## Results

### Construction of ayu reference genome

Whole-genome shotgun sequencing was carried out using one male ayu individual. Sequencing coverage was approximately 270× for short reads and 17× for long reads (S1 Table). The final genome assembly yielded 4,035 scaffolds longer than 1,000 bp. The longest scaffold was 16.8 Mb with an N50 scaffold length of 4.3 Mb. The total size of the assembly was 449.1 Mb. The genome size of ayu was estimated at approximately 420 Mb by a nuclear DNA staining method using red blood cells (S2A Fig). Thus, the total size of the assembly was relatively consistent with the estimated genome size of ayu. The genome assembly has been deposited in the DDBJ database under the accession number BNHK01000001-BNHK01004035.

To construct genetic linkage maps by pseudo-testcross mapping, GBS sequence data was obtained from an F_1_ full-sib family that included 90 siblings in addition to the two parents. Sequencing coverage was approximately 3.2× for parents and 0.6× for siblings. On average, 98.9% of GBS reads were mapped onto the ayu draft genome sequence. Variant calling identified a total of 413,386 raw SNPs, which were filtered using criteria described in the Materials and methods. A total of 5,113 filtered SNPs were used to construct the genetic linkage maps in Lep-map3 [27].

A total of 3,670 segregating SNPs were successfully classified into 28 linkage groups (LGs), which is the haploid chromosome number of ayu (S2 Table) [28]. The female-specific map contained 2,262 SNPs with a total genetic distance of 2774.10 centi Morgans (cM) and an average marker interval of 1.23 cM (S2 Table and S3 Fig). The male-specific map contained 1,394 SNPs with a total genetic distance of 2782.43 cM and an average marker interval of 2.00 cM (S2 Table and S4 Fig). The sex-averaged map contained 3,097 SNPs with a total genetic distance of 2683.18 cM and an average marker interval of 0.87 cM (S2 Table and S5 Fig). The sequences flanking all SNPs mapped to linkage maps are listed in S3 Table. Scaffolds of the ayu genome assembly were anchored to the genetic linkage maps using ALLMAPS [29]. In total, 90.7% of the genome sequences (408.3 Mb) were anchored to linkage maps, and 72.4% of them were oriented on the chromosome. Additionally, 9.3% of the genome sequences (42.0 Mb) were unplaced. Assembly completeness was evaluated by BUSCO v3.0.1 [30]. The actinopterygii_odb9 (4,584 BUSCOs) was 94.0% complete (90.1% unique and 3.9% duplicated), 2.7% fragmented, and 3.3% missing. Augustus v3.3.3 predicted 32,283 coding sequences (S4 Table) [31]. Repetitive elements in the ayu genome accounted for 56.78 Mb (12.61% of the ayu genome assembly) using the fugu repeat database and 65.15 Mb (14.47%) using the zebrafish database as predicted by RepeatMasker v4.0.5 (S2B and S2C Fig). RepeatMasker with the same parameters predicted fewer transposable elements in the ayu genome than in the genomes of rainbow trout, Atlantic salmon, northern pike and medaka.

### Conserved synteny analysis

**Fig 1:**
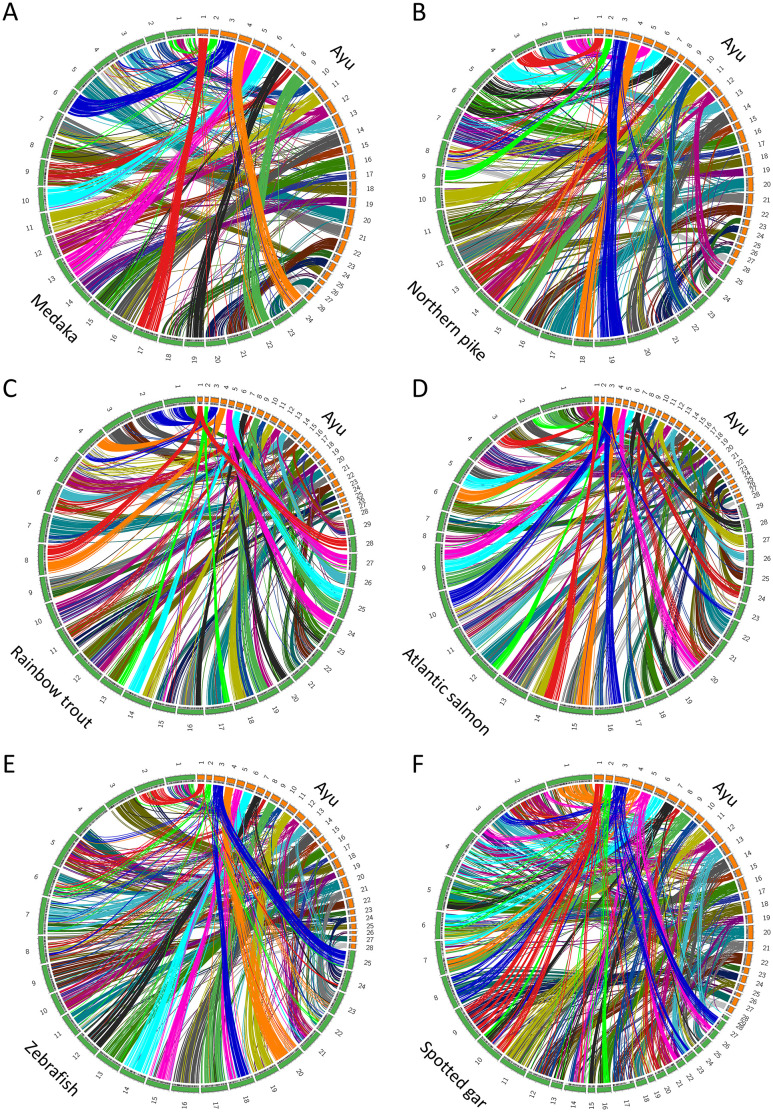
Synteny analysis between ayu genome and those of other teleost fishes. Circos plots between genomes of ayu and (A) medaka, (B) northern pike, (C) rainbow trout, (D) Atlantic salmon, (E) zebrafish and (F) spotted gar. Colored lines link pairs of orthologous genes.

To identify traces of chromosomal rearrangement in teleosts, especially Stomiati and Protacanthopterygii, we compared the ayu genome with those of other fishes. Summary for phylogenetic classification of species using conserved synteny analysis is shown in S1 Fig. Medaka (*Oryzias latipes*, Ola) belongs to the superorder Neoteleostei and the order Beloniformes. Medaka preserved its ancestral karyotype after the Teleost-specific whole-genome duplication (TGD) event [32]. The divergence of the common ancestor of ayu and medaka was estimated to have occurred approximately 180 million years ago [19,21,22]. The haploid chromosome number of medaka is 24 [32]. Synteny analysis between ayu LGs and medaka chromosomes (chr) revealed large blocks of orthologous genes with an almost one-to-one correspondence (Fig 1A and S6A and S7A Figs). However, medaka chr Ola1 corresponded to the ayu LGs Pal2 and Pal12; Ola3 corresponded to Pal10 and Pal13; Ola7 corresponded to Pal11 and Pal21; Ola9 corresponded to Pal8 and Pal25; Ola12 corresponded to Pal12 and Pal15; Ola14 corresponded to Pal17 and Pal19; and Ola20 corresponded to Pal24 and Pal26. Four chromosome fissions and fusion of a pair of chromosomes were detected as major chromosomal rearrangements.

Northern pike (*Esox lucius*, Elu) belongs to the superorder Protacanthopterygii and the order Esociformes and is phylogenetically classified as sister taxa of salmonids [19,21,22]. The divergence of the common ancestor of Protacanthopterygii and Stomiati was estimated to have occurred approximately 190 million years ago [19,21,22]. The lineages of Esociformes and Salmoniformes diverged at 75 million years ago before the SaGD event [11,19,21,22]. The haploid chromosome number of northern pike is 25 [11]. Synteny analysis between ayu and northern pike revealed large blocks of orthologous genes with an almost one-to-one correspondence (Fig 1B and S6B and S7B Figs). However, the ayu LG Pal12 corresponded to Elu14 and Elu25; Pal8 and Pal25 corresponded to Elu13; Pal12 and Pal15 corresponded to Elu14; and Pal17 and Pal19 corresponded to Elu7. As major chromosomal rearrangements, one chromosome fission and three fusions were detected.

The common ancestor of Ayu and salmonids diverged before the SaGD event [19,21,22]. The haploid chromosome number of rainbow trout and Atlantic salmon is typically 29 [33]. As expected, the syntenic blocks of ayu LGs and rainbow trout (*Onchorynchus mykiss*, Omy) chromosomes mainly showed a one-to-two correspondence. However, the ayu LG Pal12 corresponded to Omy5, Omy10, Omy12 and Omy19; Pal17 and Pal19 corresponded to Omy10, Omy12, and Omy29; and Pal26 corresponded to Omy11 (Fig 1C, S6B and S7C Figs). Similarly, ayu LGs and Atlantic salmon (*Salmo salar*, Ssa) chromosomes had a roughly one-to-two correspondence, although ayu Pal12 corresponded to Atlantic salmon chr Ssa1, Ssa4, Ssa8, and Ssa13; Pal17 and Pal19 corresponded to Ssa4, Ssa11, and Ssa13; and Pal26 corresponded to Ssa29 (Fig 1D, S6B and S7D Figs).

Zebrafish (*Danio rerio*, Dre) belongs to the order Cypriniformes. The ayu and zebrafish lineages are estimated to have diverged approximately 250 million years ago [19,21,22]. The TGD event occurred before the divergence of the zebrafish and medaka lineages [34–36]. The haploid chromosome number of zebrafish is 25 [37]. The ayu LGs Pal12, Pal16, Pal17, Pal19 and Pal20 were aligned to multiple chromosomes of the zebrafish genome, suggesting that these chromosome differences were due to translocations mostly in the zebrafish lineage. The ayu LGs Pal3, Pal5, Pal8, Pal9, Pal15, Pal21, Pal25 and Pal28 showed one-to-two syntenic relationships with zebrafish chromosomes. Other ayu LGs showed roughly one-to-one syntenic relationships with zebrafish chromosomes. Half of zebrafish Dre4 did not show any syntenic relationship with the ayu genome, as expected from its high content of repetitive elements, its heterochromatic nature, and its lack of recombination (Fig 1E and S7E Fig) [38,39]. Low similarity to Dre4 has also been reported in a comparison between Zebrafish and common carp [40].

Spotted gar (*Lepisosteus oculatus*, Loc) belongs to Holostei, the sister group of teleosts that diverged from teleosts before the TGD event [41,42]. The divergence of the common ancestor of ayu and spotted gar was estimated to have occurred approximately 300 million years ago [19,21,22]. The spotted gar genome is conserved from a vertebrate ancestor [43]. The haploid chromosome number of spotted gar is 29 [43]. Fifteen spotted gar LGs showed one-to-two syntenic relationships with ayu LGs. Orthologous blocks of spotted gar LGs Loc1–7 and Loc10 were detected in multiple ayu LGs. Orthologous chromosomes of spotted gar small LGs Loc28 and Loc29 were not detected in the ayu genome (Fig 1F and S7F Fig).

### Genome-wide association mapping to identify sex-determining locus

To explore the sex-determining locus of ayu, male and female ayu from wild populations were genotyped by GBS and whole-genome resequencing. Ayu were captured from wild populations in the Edogawa River and the Tama River. Both rivers run into Tokyo bay, and their estuaries are approximately 20 km apart as the crow flies (S8 Fig). Thus, we expected small genetic differences between the two populations because ayu is a diadromous fish. GBS data were obtained from 24 males and 24 females captured from the wild population in the Edogawa River. The mean sequencing coverage was approximately 0.7x. Whole-genome resequencing data were obtained from 10 males and nine females captured from the wild population in the Tama River. The mean sequencing coverage was approximately 12x (S5 Table). On average, 99.1% of resequencing data mapped onto the ayu reference genome sequence. We detected 2,238,473 raw SNPs by variant calling using data from both the Edogawa River population and the Tama River population. After filtering data from 34 males and 33 females genotyped by GBS and resequencing, 25,118 high-quality bi-allelic SNPs were retained (S9A Fig). The SNP density tended to be higher towards the center of chromosomes. The mean linkage disequilibrium between SNP pairs measured using r^2^ across the whole genome was estimated at 0.0224 (S.D. = 0.0440) (S6 Table and S10 Fig). The LG Pal11 had the highest average linkage disequilibrium (r^2^ = 0.0595). The r^2^ value of Pal11 was significantly higher than those of other genomic regions (Mann-Whitney U test. P = 2.2 × 10^−16^). Using these SNPs, the pairwise Fst between the Edogawa River population and the Tama River population was estimated to be 0.0198, suggesting that population differentiation between the two populations was small.

To reveal the population structure of ayu, we conducted principal component analyses (PCA). On the basis of k-means clustering of PCA scores, the Edogawa River population and the Tama River population were separated on PC1 (S11A and S11D Fig). Individuals within each population were further separated on PC2 (S11J Fig), suggesting that there is evidence of structure within the populations. The phenotypic sexes of the Edogawa River population were well separated on PC4, but those of the Tama River population were not as well separated (S11B, S11E, S11H, S11K and S11N Fig). PC3, PC5, and PC6 could not explain the genetic variances of the populations or phenotypic sexes (S11B and S11C Fig).

**Fig 2:**
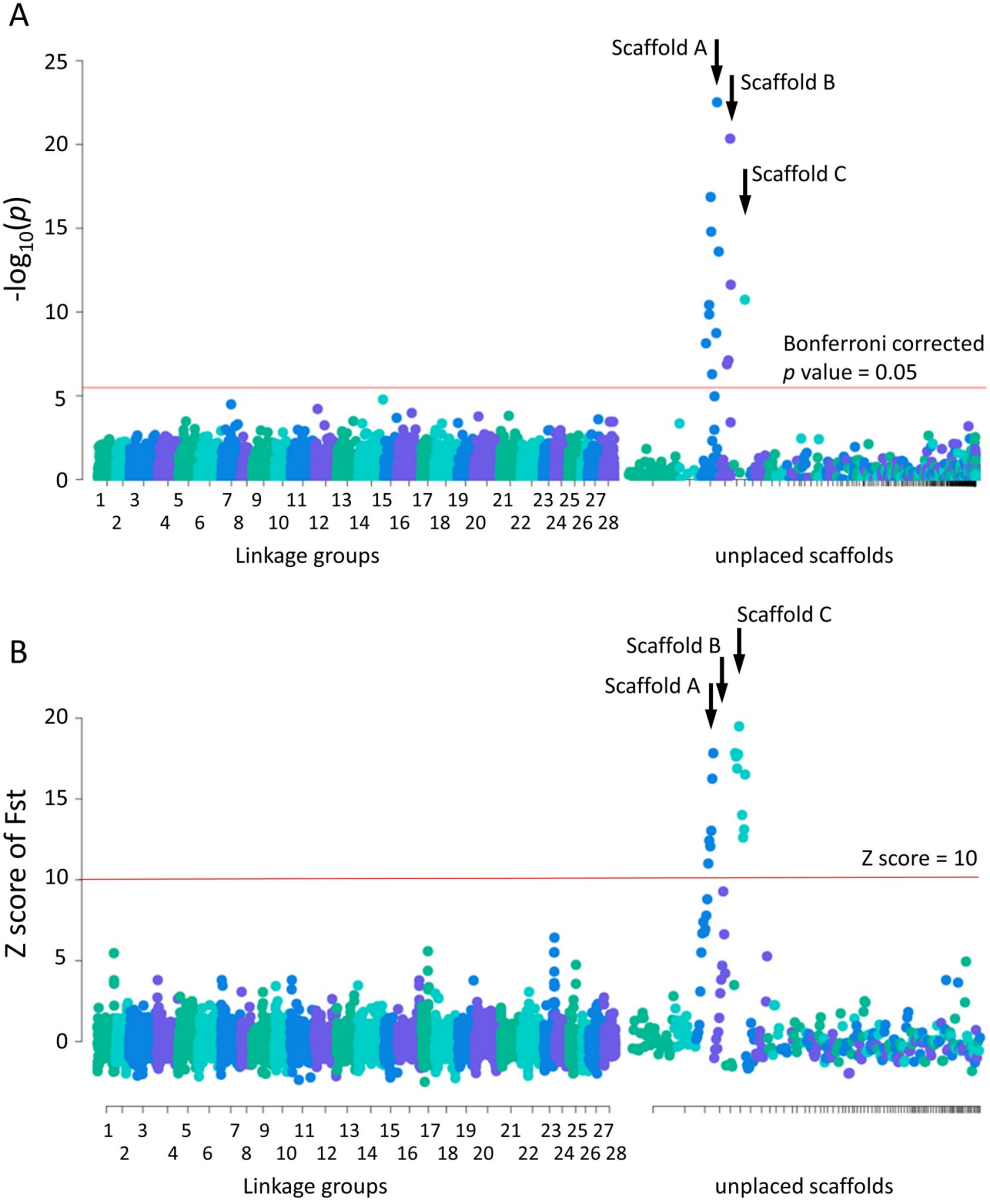
Genome-wide association analysis of sex-associated locus. (A) Manhattan plots for genome-wide association mapping of sex in ayu population. Male-associated SNPs were detected in three scaffolds. X-axis: linkage groups and scaffolds. Y-axis: −log_10_ (*p*-value) based on association test. Red line indicates genome-wide significance threshold (Bonferroni corrected *p*-value = 0.05). (B) Genomic scan for evidence of genetic divergence between males and females using SNPs detected by whole-genome resequencing. Each dot represents Z scores of Fst values calculated for a 100-kb windows in 50-kb steps along the genome. Scaffolds in which male-associated SNPs were located by genome-wide association study also had the highest Fst values.

A genome-wide association scan with the “1-dom-ref” model of GWASpoly [44] detected 15 SNPs with significant sex differences at the genome-wide level. This analysis was conducted using data from 34 males and 33 females from the Edogawa River and Tama River populations. Ten SNPs were located on unanchored scaffold A (GenBank accession number BNHK01000104), four SNPs were located on unanchored scaffold B (BNHK01000131), and one SNP was located on unanchored scaffold C (BNHK01000134) (Fig 2A and S12 Fig). One SNP at position 849,395 on scaffold A showed the most significant association with sex (*p* = 5.18 × 10^−27^). The lengths of the sex-linked scaffolds were 869.3 kb (scaffold A), 596.3 kb (scaffold B) and 568.8 kb (scaffold C).

To confirm that the loci detected by the genome-wide association study were sex-linked, we searched for evidence of genetic divergence between males and females using Fst as an index with 444,151 SNPs detected by whole-genome resequencing of 10 males and nine females in the Tama River population (S9B Fig). Genomic regions with high genetic differentiation (Z score of Fst > 10) between sexes were detected in scaffolds A and C (Fig 2B). The highest Fst was for 200,001–300,000 bp in scaffold C (Weir and Cockerham weighted Fst = 0.47, Z score of Fst = 19.49). The mean DNA nucleotide diversity index π in scaffold C was estimated to be 0.000213, which was higher than mean of whole genome 0.000343, suggesting that mutations accumulated in scaffold C (Welch’s *t* test *p* = 0.003).

To validate genome-wide association scan for sex determining locus of ayu, we performed linkage analysis of genetic sex using ayu F_1_ full-sib families captured from a wild population in the Nagaragawa River. Mapping families were generated from artificial fertilization of two pair of ayu and the siblings were mixed and reared until the phenotypic sex could be identified. GRAS-Di (Genotyping by Random Amplicon Sequencing-Direct) sequence data was obtained from two F1 full-sib families that included 88 siblings (44 females and 44 males) and two pair of parents. Sequencing coverage was approximately 5.2x for parents and 2.6× for siblings. In F_1_ siblings, 30 females and 31 males were assigned to family 1 and 14 females and 13 males were assigned to family 2 by SNP-based parentage assignment. The male-specific map of family 1 identified 33 linkage groups and contained 1,978 SNPs with a total genetic distance of 1398.30 cM and an average marker interval of 0.71 cM (S7 Table and S13 Fig and S1 Text). Simple interval mapping for genetic sex identified a significant peak on linkage group PalSex (LOD score 18.36) (S14 Fig). The 1.5 LOD support interval was from 50.0 to 54.2 cM. Position of the sex associated linkage group in the genome assembly was shown in S14B Fig. The male-specific map of family 2 identified 34 linkage groups and contained 1,309 SNPs with a total genetic distance of 962.93 cM and an average marker interval of 0.74 cM (S8 Table and S15 Fig). Simple interval mapping for genetic sex identified a significant peak on linkage group PalSex2 (LOD score 8.12) (S16 Fig). Position of the sex associated linkage group in the genome assembly was shown in S16B Fig. Compared to family 1, sex associated linkage group was divided into two linkage groups in family 2 (referred to PalSex and PalSex2). The 1.5 LOD support interval was from 8.0 to 14.0 cM. SNPs located in Scaffold A, Scaffold B and Scaffold C that were detected as sex-links scaffold by GWAS using the Edogawa River and the Tama River population were also associated with genetic sex by linkage analysis using the ayu derived from the Nagaragawa River population.

### Identification of ayu sex-determining gene candidates

**Fig 3:**
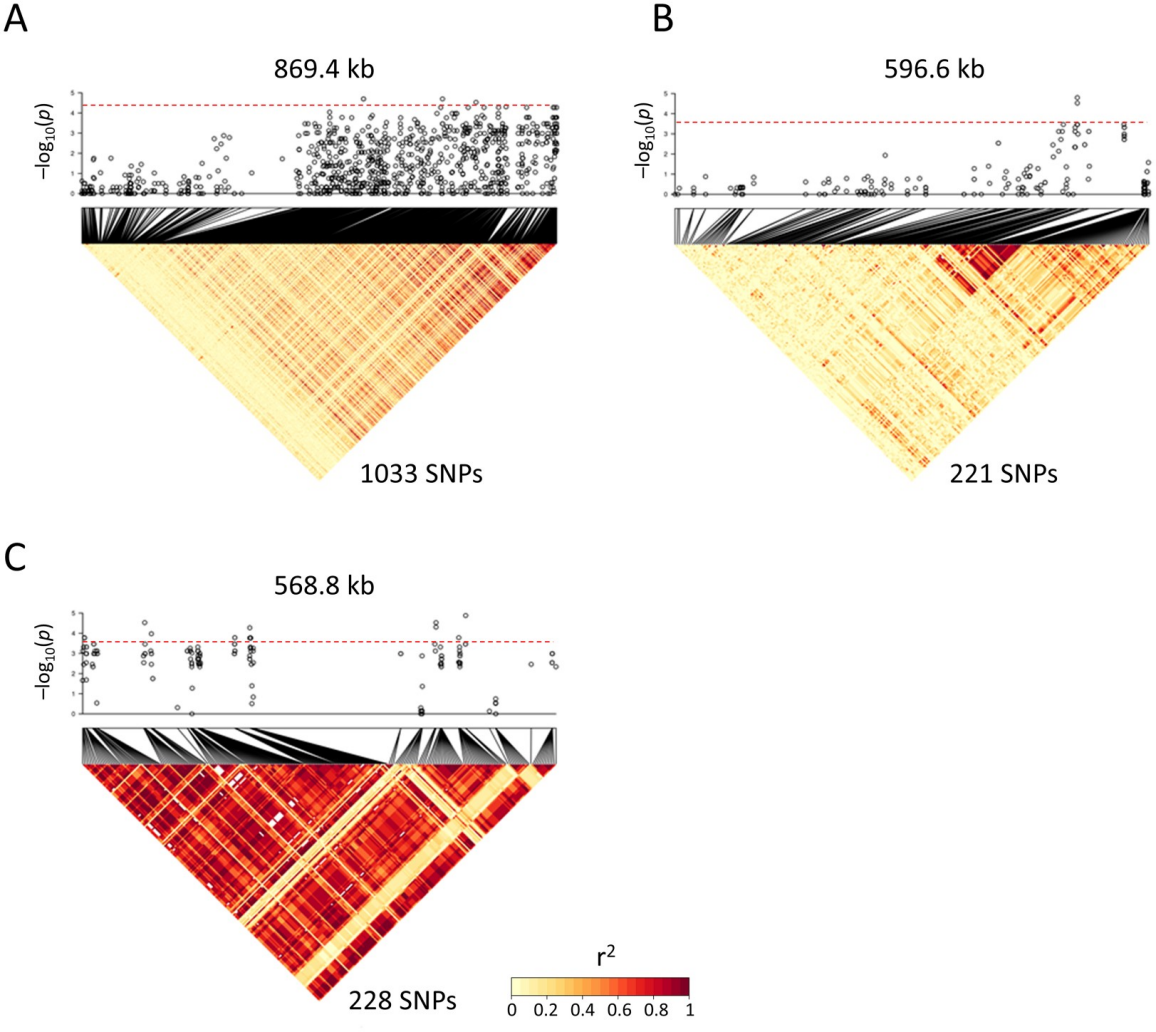
Linkage disequilibrium analysis of sex-associated scaffolds. Figure shows heat maps of pairwise linkage disequilibrium (r^2^) between SNPs (lower column) and scaffold-specific high-resolution association test (upper column) within sex-associated scaffolds using SNPs genotyped by resequencing using ten males and nine females. (A) Scaffold A, (B) scaffold B and (C) scaffold C. Very strong linkage disequilibrium blocks were detected in scaffold C (C). X-axis indicates physical position of scaffolds. The numbers of SNPs in the figure indicate that the total number of SNPs using calculation for pairwise r^2^ value and scaffold-specific association test. Red line indicates scaffold-wide significance threshold (Bonferroni-corrected *p*-value = 0.05). Color of each SNP reflects pairwise r^2^ value: red indicates higher values, yellow indicates lower values.

To explore ayu sex-determining gene candidates, we analyzed the three sex-linked scaffolds in detail using linkage disequilibrium analysis and scaffold-wide association tests. In general, the sex-linked SNPs showed strong linkage disequilibrium [9]. The SNP intervals detected from the GBS data were too wide for detailed analyses, so we used SNPs with a minor allele frequency >20% detected by whole-genome resequencing of 10 males and nine females from the Tama River population (S9 Fig). We detected 1,033, 221 and 228 SNPs in the sex-linked scaffolds A, B and C, respectively. In scaffold A with 1,033 SNPs, *p*-values for association tests between SNP genotype and sex tended to be higher toward the 3′ end of the scaffold (Fig 3A). Four SNPs were associated at a scaffold-wide level of significance (Bonferroni-corrected p-value < 0.05; *p* < 4.8 × 10^−5^). Similarly, in scaffold B with 221 SNPs, associations between SNP genotypes and sex were detected at the 3′ end of the scaffold (Fig 3B). Two SNPs were associated at a scaffold-wide level of significance (*p* < 2.3 × 10^−4^). Associations between phenotypic sex and blocks with strong linkage disequilibrium were detected throughout scaffold C using 228 SNPs (Fig 3C). Seventeen SNPs were significantly associated with sex across scaffold C (*p* < 2.2 × 10^−4^). In scaffold C, 10.02% of sequences were predicted to be transposable elements by RepeatMasker by comparison with the fugu repeat database, whereas 1.70% and 2.25% of sequences were predicted as transposable elements in scaffold A and scaffold B, respectively (mean across the whole genome, 1.14%).

**Fig 4:**
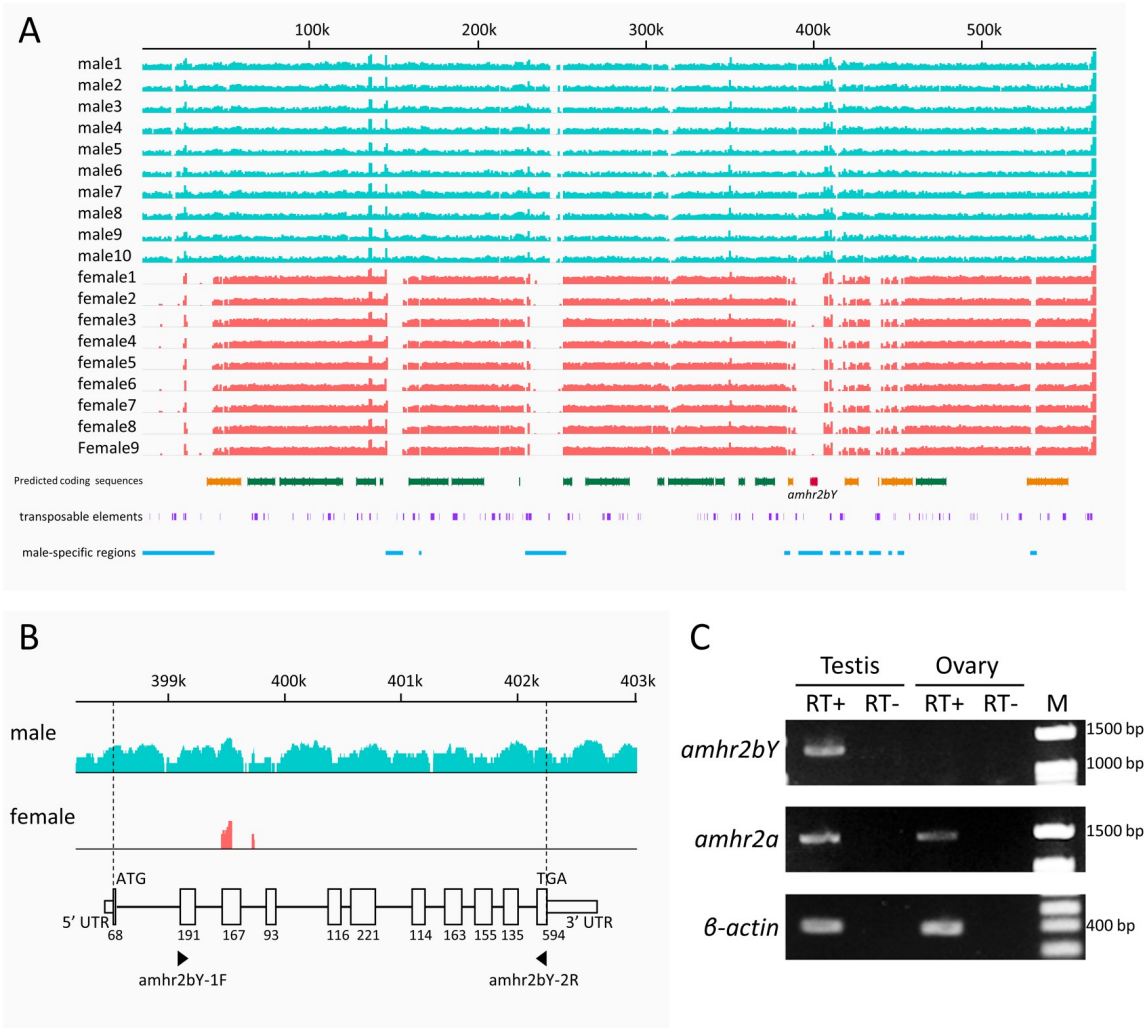
Comparison of mapping coverage of whole-genome resequencing between males and females in sex-linked scaffolds. (A) Comparison of sequence depth between males and females in sex-associated scaffold C. Y-axis indicates log-scaled sequence depth in whole-genome resequencing analysis; X-axis indicates physical position along the scaffold. Green boxes indicate predicted coding sequences. Red boxes indicate *amhr2bY*. Orange boxes indicate Y-specific candidate coding sequences. Purple boxes indicate putative transposable elements. Blue lines indicate putative male-specific regions. (B) Magnification of region from 399–403 kb in (A) and schematic of gene structure of candidate sex-determining gene *amhr2bY*. Numbers refer to exon sizes. Arrowheads indicate locations of PCR primers for RT-PCR used in C. (C) Expression analysis of *amhr2bY* and autosomal *amhr2a* mRNA in testes and ovaries of immature individuals by RT-PCR. RT+: Reverse transcription reaction with reverse transcriptase. RT-, RT reaction without reverse transcriptase. M: size marker.

In ayu, sex-reversal experiments indicate that genetic sex is determined by an XX/XY system [24]. To identify Y chromosome-specific regions in sex-linked scaffolds, we compared mapping coverage of whole-genome resequencing between males and females in the Tama River population. In scaffold A, Y chromosome-specific regions of resequencing reads were mapped for males (XY) but not females (XX), and were detected at approximately 775–783, 805–812, 834–839, and 858–869 kb (S17A Fig, blue line). In scaffold B, Y chromosome-specific regions were detected at approximately 559–565 kb (S17B Fig, blue line). In scaffold C, Y chromosome-specific regions were detected at approximately 0–52, 146–158, 228–251, and 384–454 kb (Fig 4A, blue line). These putative Y chromosome-specific regions contained 23 putative coding sequences. Aside from transposon-related coding sequences, 10 coding sequences were identified as candidates for the ayu sex-determining gene: *pgta*, encoding geranylgeranyl transferase type-2 subunit alpha; *eaa5a*, encoding excitatory amino acid transporter 5a; *amhr2*, encoding anti-Muellerian hormone type-2 receptor; *eaa5b*, encoding excitatory amino acid transporter 5b; *gtd2a*, encoding general transcription factor II-I repeat domain-containing protein 2A; *sgta*, encoding small glutamine-rich tetratricopeptide repeat-containing protein alpha; *thop1*, encoding thimet oligopeptidase; *syde2*, encoding rho GTPase-activating protein SYDE2; *wdr63*, encoding WD repeat-containing protein 63; and *gels* encoding gelsolin (S9 Table). Neither the sex-determining gene of salmonids, *sdY*, nor its parent gene, *irf9*, were located in this region. This was expected, because these genes are also absent from the genome of northern pike [8,11].

We ruled out nine of the putative candidate sex determining genes using the following lines of evidence. Genomic PCR analyses using DNA extracted from four males and four females suggested that *gtd2a* and *wdr63* were male specific; *eaa5a*, *sgta* and *thop1* had some Y-specific insertions and deletions in intron regions; and *pgta*, *eaa5b*, *syde2* and *gels* were not male specific (S18 Fig). In RT-PCR analyses of differentiated testes and ovaries, *eaa5b* mRNA was detected in testes but not ovaries, *gtd2a* and *wdr63* mRNAs were not detected in gonads, and mRNAs of the other six candidate genes were detected in both testes and ovaries (S19 Fig). The gonads could not be dissected from ayu at the early sex differentiation stage, because the body size was too small. We focused on the predicted coding sequences showing similarity to *amhr2* as a strong candidate for the ayu sex-determining gene, because *amh* signaling is critical for sex differentiation among vertebrates and *amhr2* is known to be the sex-determining gene in fugu and yellow perch [2,10–14,45].

Reference genome sequence was constructed from one XY male ayu individual. Thus, sex-linked scaffolds were probably a chimera of Y chromosome and X chromosome. To confirm sequence of sex-linked scaffold around strong candidate for the ayu sex-determining gene, we screened and sequenced a 66 kb BAC (Bacterial Artificial Chromosome) clone carrying sex-linked *amhr2*. Comparison of mapping coverage of whole-genome resequencing between males and females in the BAC clone sequence reveled that sex-linked *amhr2* was certainly male-specific (S1 Text and S20A Fig). We then examined the BAC clone carrying sex-linked *amhr2* by linkage disequilibrium analysis and scaffold-wide association tests by whole-genome resequencing of 10 males and nine females from the Tama River population. Associations between phenotypic sex and blocks with strong linkage disequilibrium were detected throughout sex-linked BAC clone using 240 SNPs with a minor allele frequency > 20% (S20B Fig). Eleven SNPs were significantly associated with sex in the BAC clone (*p* < 2.1 × 10^−4^). In addition, the order of predicted genes around Y-linked *amhr2* was the same between scaffold C and BAC clone (S20C Fig). Thus, we considered that whole genome sequences were reliable and carried out the subsequent analysis. Further studies will need to obtain accurate sequence of Y chromosome and X chromosome such as whole genome shotgun sequence of XX individual and YY individual.

### Molecular cloning of candidate sex-determining gene

We obtained the cDNA fragment of the Y-linked *amhr2* by RT-PCR using primers designed based on the predicted coding sequence in male-specific regions of scaffold C. Then, 5′ and 3′ rapid amplification of cDNA ends (RACE) yielded a 2017-bp full-length cDNA composed of 11 exons encoding 494 amino acids (GenBank accession number LC512011; Fig 4B). The *amhr2Y* gene was located at 398–403 kb in a male-specific region of scaffold C. Next, PCR analyses of the 12 males and 12 females used in the association study confirmed that *amhr2Y* is Y chromosome-specific (S21 Fig). In addition to the sex-linked *amhr2Y*, we blasted the cDNA sequence of *amhr2Y* against the ayu genome sequence and found an autosomal *amhr2* located at 5.09 Mb in LG Pal26. The cDNA fragment of autosomal *amhr2* was obtained by RT-PCR using primers designed based on the predicted coding sequence in LG Pal26. Subsequently, the full-length cDNA of autosomal *amhr2* was cloned by 5′ and 3′ RACE. The full-length cDNA of autosomal *amhr2* was 2,298 bp long, and encoded 509 amino acids (GenBank accession number LC512012). The autosomal *amhr2* was named *amhr2a* and the Y-linked *amhr2* was named *amhr2bY*. In RT-PCR analyses, *amhr2bY* mRNA was detected in testes but not ovaries of immature individuals (5 months post fertilization). Transcripts of *amhr2a* were detected in both testes and ovaries (Fig 4C).

In a molecular phylogenetic analysis using the maximum-likelihood method, the Amhr2bY and Amhr2a in ayu clustered with Amhr2 of other teleosts and were most closely related to those in Northern Pike, Rainbow trout, and Atlantic salmon. These results indicated that the duplication giving rise to Amhr2bY was an ayu-specific event (S22 Fig). The deduced amino acid sequences of Amhr2bY and Amhr2a shared 84% similarity. According to homology with human AMHR2 (Protein Data Bank ID: Q16671) [46], activin type I and II receptor domains (S23 Fig, blue box), transmembrane domains (S23 Fig, green box) and catalytic domains of serine/threonine kinases, bone morphogenetic protein, (S23 Fig, red box) were well conserved between Amhr2bY and Amhr2a. Multiple alignment of the ayu Amhr2bY and Amhr2a sequences with those of other AMHR2 family members in teleosts revealed that 17 out of 19 amino acids that are evolutionarily conserved in the ATP binding site in the catalytic domain of serine/threonine kinases in the BMPR2/AMHR2 family (as annotated in the NCBI conserved domain database) [47] were conserved in ayu (S23 Fig, white arrowheads). In the ATP binding site, the 267^th^ amino acid in Amhr2bY was glutamine (Q, a polar amino acid), while that at the same position in Amhr2 family members in other teleosts, including ayu Amhr2a, was a hydrophobic amino acid (S23 Fig, red arrowhead and S24A Fig). In addition, Amhr2bY contained many specific amino acid changes (S23 Fig, blue arrows). Amhr2a contained a specific 15-amino acid insertion in the serine/threonine kinase domain (S23 Fig, blue line). This 15-amino acid insertion did not exist in Amhr2bY and Amhr2 of all other species. This insertion was confirmed by RT-PCR using RNA extracted from different individuals from those used to clone the full-length cDNA (S24C Fig). The 15-amino acid insertion in Amr2a was located in a relatively unconserved region. In the structure predicted by SWISS-MODEL, the 15-amino acid insertion was located outside the active region of Amhr2a (S24B Fig) [48].

### Genomic comparison of *amhr2bY* and autosomal *amhr2a*

To obtain insight into the Y-specific duplication of *amhr2*, we compared genomic regions located in and around *amhr2bY* on scaffold C and autosomal *amhr2a* on LG Pal26. Pairwise genome alignments of the 30-kb genomic sequence around *amhr2bY* and *amhr2a* revealed that approximately 10 kb of the 5′ region immediately upstream and 10 kb of the 3′ region immediately downstream of the genes were dissimilar (S25 Fig). Prediction of transcription factor binding sites detected 12,619 binding sites for 566 transcription factors in the 5′ intergenic region between *amhr2bY* and its adjacent gene (putative promoter region, 7,323 bp) (S10 Table). Comparisons of the regulatory regions of *amhr2s* among ayu, northern pike and medaka, revealed 26 transcription factors that regulated only *amhr2bY* (S26 Fig).

**Fig 5:**
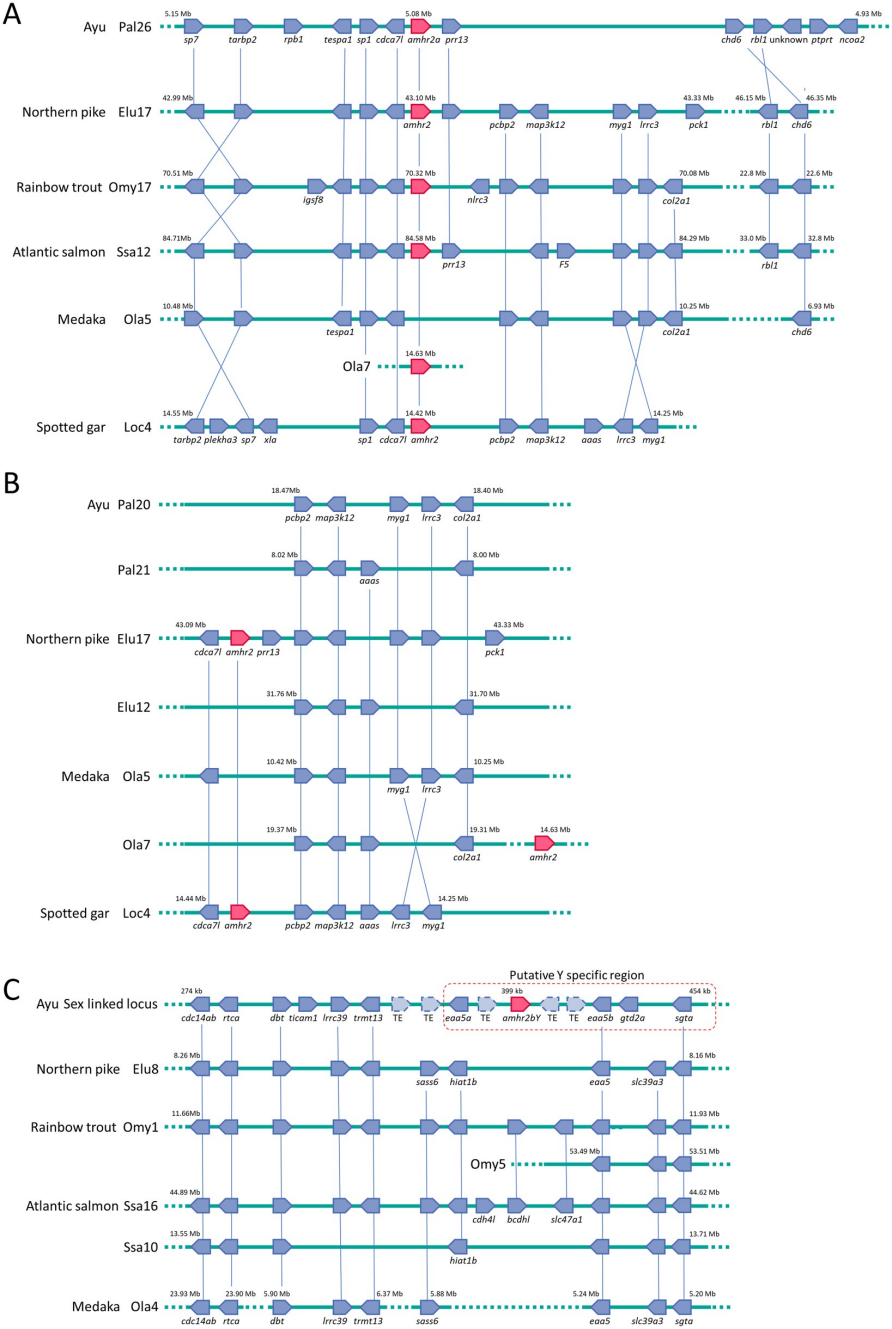
Summary of conserved syntenic relationships of *amhr2*s between ayu and other fishes. (A) Conserved syntenic relationships of autosomal *amhr2a*. The region from autosomal *amhr2a* to *sp7* on linkage group Pal26 in ayu showed conserved syntenic relationships to chromosome Elu17 in northern pike, Omy17 in rainbow trout, Ssa12 in Atlantic salmon, Ola5 excluding *amhr2* in medaka and Loc4 in spotted gar. The region including *pcbp2* to *col2a1* was not detected in Pal26 in ayu. No conserved syntenic relationships were detected between Pal26 and medaka Ola7, which contains *amhr2*. (B) Traces of teleost-specific whole-genome duplication and ayu-specific translocation of region containing *amhr2a*. The region containing *pcbp2* to *col2a1*, the adjacent syntenic block to the region containing *amhr2*, was located in linkage group Pal20 and Pal21 in ayu. These regions showed conserved syntenic relationships to chromosome Elu12 and Elu17 in northern pike, Ola5 and Ola7 in medaka and Loc4 in spotted gar. (C) Conserved syntenic relationships of putative sex-determining locus of ayu. Syntenic blocks surrounding *amhr2bY* on sex-linked scaffolds (scaffold C) were evolutionarily conserved with Elu8 in northern pike, Omy1 and Omy5 in rainbow trout, Ssa10 and Ssa16 in Atlantic salmon and Ola4 in medaka. Region containing *amhr2bY* in ayu did not contain three of ten genes conserved among northern pike, rainbow trout and Atlantic salmon. TE indicates putative transposable element. *Sp7*: transcription factor Sp7, *tarbp2*: RISC-loading complex subunit tarbp2, *tespa1*: thymocyte expressed, positive selection associated 1, *rpb1*: putative DNA-directed RNA polymerase II subunit RPB1-like isoform, *sp1*: transcription factor Sp1, *cdca7l*: cell division cycle-associated protein 7-like, *chd6*: chromodomain-helicase-DNA-binding protein 6-like, *rbl1*: retinoblastoma-like protein 1 isoform X3, *ptprt*: receptor-type tyrosine-protein phosphatase T, *ncoa2*: nuclear receptor coactivator 2, *prr13*: proline-rich 13, *pcbp2*: poly(rC) binding protein 2, *map3k12*: mitogen-activated protein kinase kinase kinase 12, *myg1*: melanocyte proliferating gene 1, *lrrc3*: leucine-rich repeat-containing protein 3, *pck1*: phosphoenolpyruvate carboxykinase, *nlrc3*: NLR family CARD domain-containing protein 3-like, *igsf8*: immunoglobulin superfamily member 8-like, *col2a1*: collagen type II alpha 1 chain, *F5*: coagulation factor V-like, *plekha3*: pleckstrin homology domain-containing family A member 3-like, *xla*: hypothetical protein XELAEV_18036955mg, *aaas*: adracalin, *cdc14ab*: cell division cycle 14Ab, *dbt*: lipoamide acyltransferase component of branched-chain alpha-keto acid, *ticam1*: TIR domain-containing adapter molecule 1-like, *eaa5a*: excitatory amino acid transporter 5a, *eaa5b*: excitatory amino acid transporter 5b, *gtd2a*: general transcription factor II-I repeat domain-containing protein 2A, *sgta*: small glutamine-rich tetratricopeptide repeat-containing protein alpha, *rtca*: RNA 3’-terminal phosphate cyclase, *lrrc39*: leucine rich repeat containing 39, *trmt13*: tRNA methyltransferase 13 homolog, *sass6*: SAS-6 centriolar assembly protein, *hiat1b*: hippocampus abundant transcript 1b, *slc39Aa*: solute carrier family 39 member 3 (Zinc transporter, zip3), *cdh4l*: cadherin4-like, *bcdhl*: B-cadherin-like and Slc47a1; solute carrier family 47 member 1 (multidrug and toxin extrusion protein 1, mate1).

To obtain traces of Y-specific duplication of *amhr2*, we compared conserved synteny around *amhr2bY* and *amhr2a* in ayu with those of other fishes. Syntenic blocks including *amhr2a* on LG Pal26 were evolutionarily conserved with chromosome Elu17 in northern pike, Omy17 in rainbow trout, Ssa12 in Atlantic salmon, Ola5 excluding *amhr2* in medaka and Loc4 in spotted gar (Fig 5A). The syntenic block including *pcbp2* to *col2a1* adjacent to the conserved syntenic block containing *amhr2a* was not detected in Pal26 in ayu. No conserved syntenic relationships were detected between Pal26 and Ola7 (containing *amhr2*) in medaka. Conserved synteny analyses using whole genomes suggested that the orthologous chromosome of Elu17, Omy17, Ssa12, Ola5 and Loc4 was Pal20, not Pal26, in ayu (Fig 1 and S7 Fig). In medaka, *amhr2* located on chromosome Ola7 was not located in the syntenic block conserved between fish on Ola5. In medaka, comparative gene mapping revealed that Ola5 and Ola7 are ohnologs of proto-chromosome 3 in the common ancestor of teleost fish before the TGD event [49]. The orthologous chromosome of Ola5 was Pal20 in ayu and that of Ola7 was Pal21 (Fig 1A and S7A Fig). As expected, the conserved syntenic block including *pcbp2* to *col2a1* that was not detected in Pal26 was located on Pal20 and Pal21 in ayu (Fig 5B). These regions also showed conserved syntenic relationships with Elu17 and Elu12 in northern pike and Loc4 in spotted gar.

Syntenic blocks surrounding *amhr2bY* on sex-linked scaffolds were evolutionarily conserved with Elu8 in northern pike, Omy1 and Omy5 in rainbow trout, Ssa10 and Ssa16 in Atlantic salmon and Ola4 in medaka (Fig 5C). Conserved synteny analysis using whole genomes revealed that Elu8, Omy1, Omy5, Ssa10, Ssa16 and Ola4 were orthologous chromosomes of ayu Pal11 and Pal28 (Fig 1 and S7 Fig). The *amhr2bY*-containing region in the ayu genome did not contain three out of 10 genes conserved among northern pike, rainbow trout and Atlantic salmon. Instead of these conserved genes, *amhr2bY*, *ticam1* (encoding TIR domain-containing adapter molecule 1-like), an extra copy of *eaa5*, *gtd2a*, and putative transportable element-related coding sequences were located in the *amhrb2bY*-containing region in the ayu genome.

### Expression of *amhr2bY* during early gonadal differentiation

The Y-linked sex-determining gene functions as the first trigger of differentiation from sexually undifferentiated gonads to testes. If *amhr2bY* indeed determines the genetic sex of ayu, it should be expressed in sexually undifferentiated XY gonads during early sex differentiation. To investigate the expression of *amhr2bY* and *amhr2a* in sexually undifferentiated gonads, we first histologically observed the gonadal development of ayu under our rearing conditions after artificial fertilization. The gonad is located in the body cavity between the coelomic epithelium and the gut (S27 Fig, dotted line and S1 Text). At 39 days post fertilization (total body length: approximately 8 mm), no gonadal primordia were observed in future gonadal regions in both genetic males and females (S27A and S27B Fig). At 2 months post fertilization (mpf) (total body length: approximately 20 mm), no morphological differences between genetic males and females were observed (S27C and S27D Fig). In general, meiosis is one of the earliest ovarian differentiation features [50] as it is initiated earlier in teleost female gonads than in male gonads. Oocyte meiosis in female gonads was not observed at 2 mpf. At 3.5 mpf (total body length: approximately 40 mm), spermatogenesis had not initiated in male gonads (S27E Fig). Oogenesis proceeded in female gonads, and the oocytes entered the diplotene stage at 3.5 mpf (S27F Fig). At 7 mpf (total body length approximately 100 mm and body weight approximately 12 g), spermatogenesis had begun in male gonads (S27G Fig) and ovarian follicles had developed to the vitellogenic stage (S27H Fig). Thus, the sex differentiation period in ayu was between 2 and 3.5 mpf under our rearing conditions.

To monitor the transcript levels of the Y-linked *vs*. the autosomal *amhr2*, we had to distinguish the two, despite the high sequence similarity between *amhr2bY* and *amhr2a* (S23 and S28 Figs). An RNA probe for an *amhr2bY*-containing ORF detected both *amhr2bY* and *amhr2a* (S29A–S29D and S29I Fig). RNA probes against the 3′ untranslated region (UTR) of *amhr2bY* and *amhr2a* mRNA distinguished between these genes in *in situ* hybridization analyses (S28 and S29 Figs and S1 Text). The *amhr2bY* probe detected transcripts specifically in undifferentiated gonads of genetic males at 2 mpf (S29E and S29F Fig). Autosomal *amhr2a* mRNA was detected in both XY and XX undifferentiated gonads at 2 mpf (S29G and S29H Fig).

**Fig 6:**
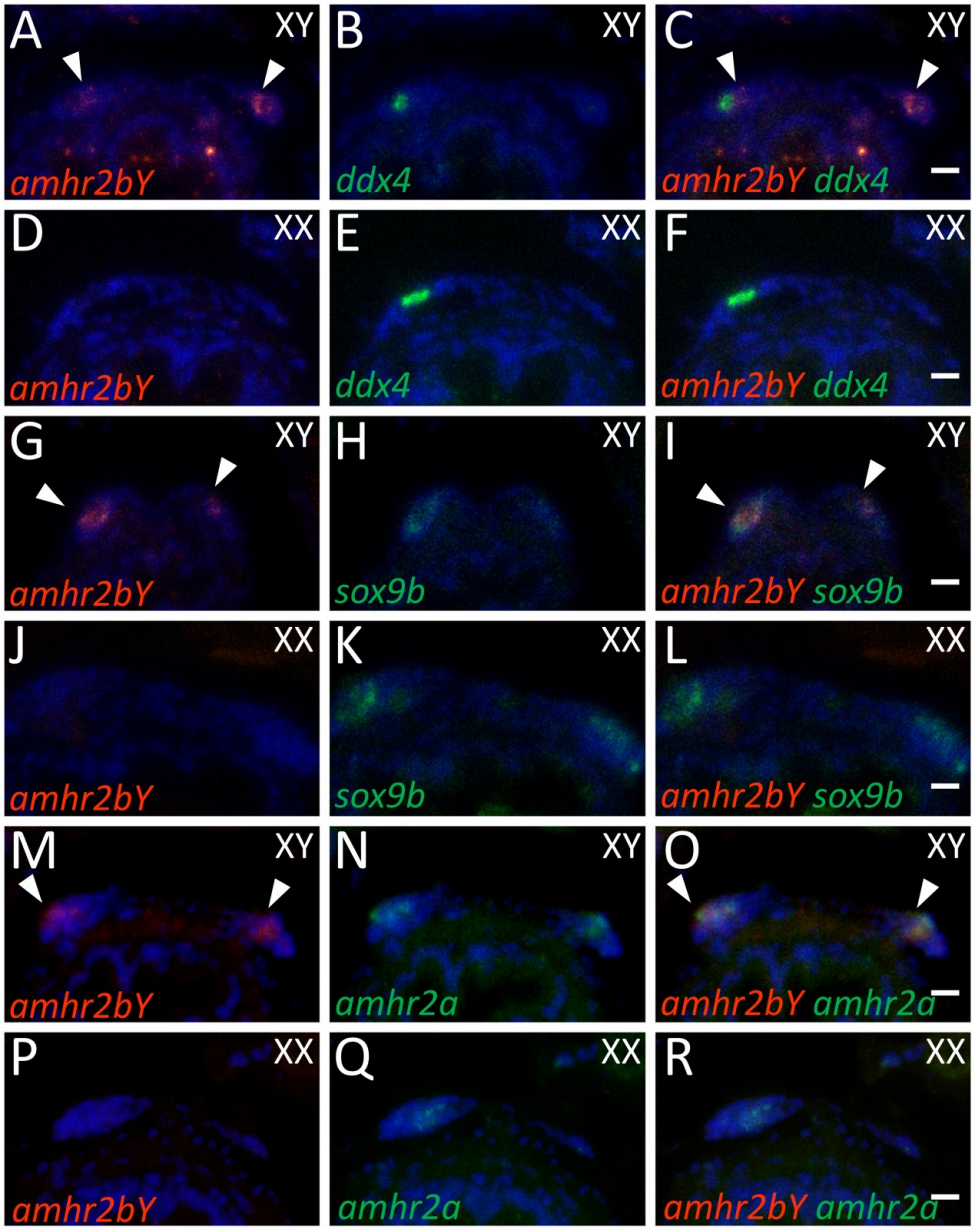
Expression of *amhr2bY* in morphologically undifferentiated gonads in ayu. (A–F) Two-color section *in situ* hybridization showing *amhr2bY* (A and D, red) and germ cell marker *ddx4* (B and E, green) in XY (A–C) and XX (D–F) gonads at 2 months post fertilization (mpf). (G–L) Two-color section *in situ* hybridization showing *amhr2bY* (G and J, red) and *sox9b* (H and K, green) in sexually undifferentiated somatic cells in XY (G–I) and XX (J–L) gonads at 2 mpf. (M-R) Two-color section *in situ* hybridization showing *amhr2bY* (M and P, red) and autosomal *amhr2a* (N and Q, green) in XY (M–O) and XX (P–R) gonads at 2 mpf. *amhr2bY* mRNA specifically accumulated in somatic cells surrounding germ cells in morphologically undifferentiated gonads of genetic males. Autosomal *amhr2a* mRNA was expressed in somatic cells in morphologically undifferentiated gonads of both genetic males and females. Transcripts of *amhr2bY* and *amhr2a* co-localized in somatic cells. Arrowheads indicate *amhr2Y*-positive signals. Scale bar: 10 μm.

**Fig 7:**
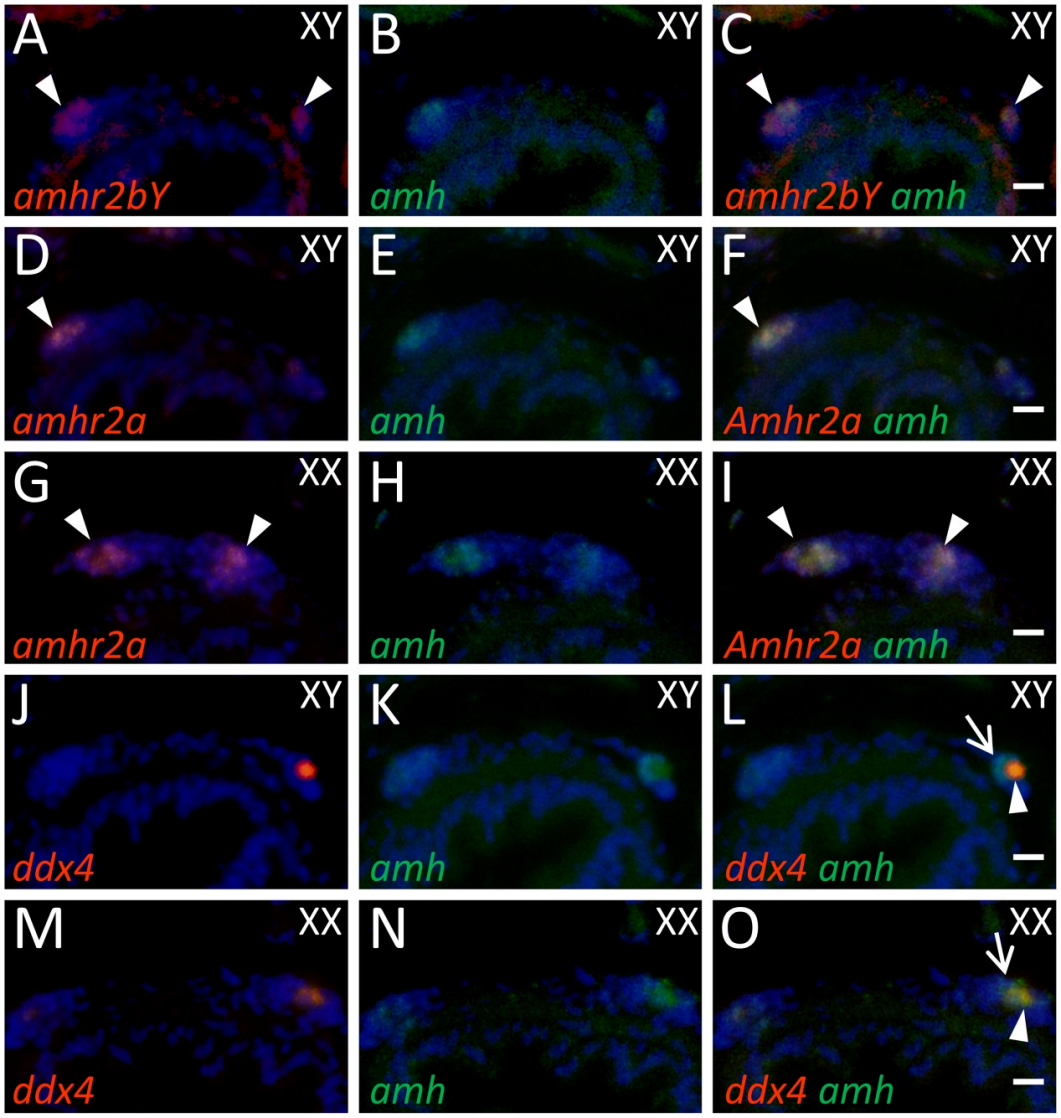
Expression of *amhr2s* and *amh* (encoding putative ligand of *Amhr2*) in morphologically undifferentiated gonads. (A–C) Two-color section *in situ* hybridization showing *amhr2bY* (A, red) and *amh* (B, green) transcripts in XY gonads at 2 months post fertilization (mpf). (D–I) Two-color section *in situ* hybridization showing *amhr2a* (D and G, red) and *amh* (E and H, green) transcripts in XY (D–F) and XX (G–I) gonads at 2 mpf. (J–O) Two-color section *in situ* hybridization showing germ cell marker *ddx4* (J and M, red) and *amh* (K and N, green) transcripts in XY (J–L) and XX (M–O) gonads at 2 mpf. Panels show that both *amhr2* and *amh* co-localize in somatic cells in morphologically undifferentiated gonads. *amh* transcripts are also present in *ddx4*-positive germ cells. Arrowheads in A–I indicate *amhr2bY*- and autosomal *amhr2a*-positive signals. Arrowheads in L and O indicate germ cells co-expressing *amh* and *ddx4*. Arrows in L and O indicate *amh*-positive somatic cells. Scale bar: 10 μm.

To determine the cell type of *amhr2bY*-expressing cells, we compared the expression patterns of *amhr2bY* and marker genes for gonadal differentiation. The germ-cell marker gene *ddx4* (DEAD-box helicase 4, sometimes called *vasa*) is essential for development and differentiation of germ cells in many teleosts [51,52]. We detected *amhr2bY* mRNA in somatic cells surrounding *ddx4*-positive germ cells in morphologically undifferentiated gonads of genetic males, but not females, at 2 mpf (Fig 6C and 6F). *sox9b* (sometimes called *sox9a2*) is expressed in undifferentiated supporting cells surrounding germline stem cells in both males and females and is critical for maintaining sexually undifferentiated germline stem cells in medaka [53,54]. We detected *sox9* binding sites in the putative regulatory regions of *amhr2bY* and *amhr2a* (S10 Table and S26 Fig). In ayu, *sox9b* mRNA was detected in somatic cells of morphologically undifferentiated gonads of both genetic males and females at 2 mpf (Fig 6H and 6K). Transcripts of *amhr2bY* and *sox9b* co-localized in somatic cells of morphologically undifferentiated male gonads at 2 mpf (Fig 6I and 6L). Both *amhr2bY* and autosomal *amhr2a* mRNAs co-localized in somatic cells of morphologically undifferentiated male gonads at 2 mpf (Fig 6O and 6R). Transcripts representing *amh*, the putative ligand of *amhr2*, were detected in both somatic cells and *ddx4*-positive germ cells of morphologically undifferentiated gonads in both sexes (Fig 7J–7O). Transcripts of both *amhr2bY* and *amh* were detected in the same somatic cells in male gonads at 2 mpf (Fig 7A–7C). Transcripts of *amhr2a* and *amh* were colocalized in somatic cells in both males and females at 2 mpf (Fig 7D–7I).

### Loss-of-function analysis of *amhr2bY*

**Fig 8:**
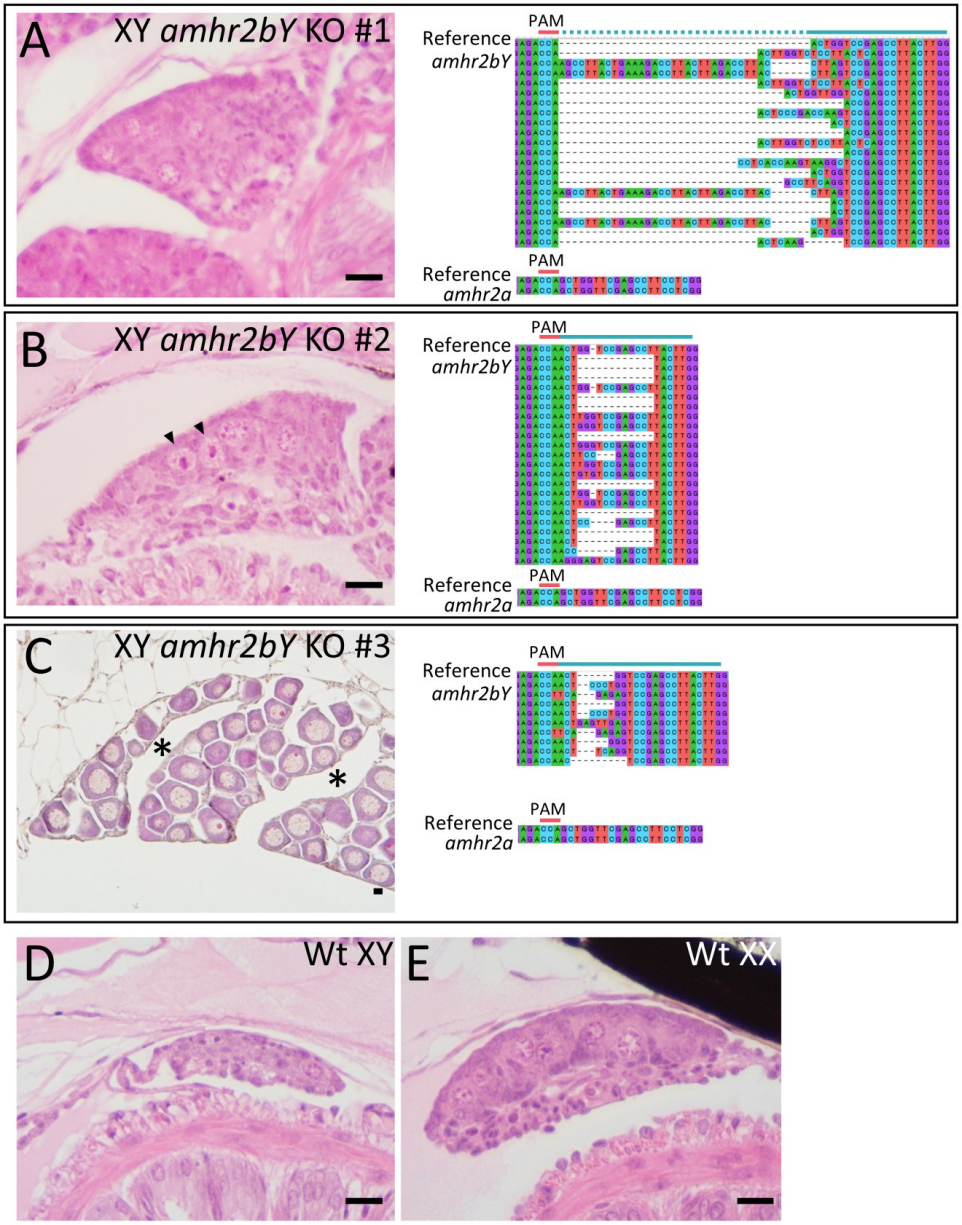
Loss of function analysis for *amhr2bY* of ayu in G0 generation using CRISPR/Cas9 system. (A and B) The gonad of *amhr2bY-*knockout positive XY individuals at 3 mpf. (C) The gonad of *amhr2bY*-knockout positive XY individuals at 6 mpf. The gonad of *amhr2bY*-knockout positive XY ayu individuals were differentiated to female gonad. Tissue sections were stained with hematoxylin–eosin. (A) -5 bp to +32 bp insertion / deletion were detected in XY individual #1, leading to a frame shift. (B) -11 bp to +1 bp insertion / deletion were detected in XY individual #2, leading to a frame shift. (C) +1 bp, +3 bp, +4 bp, +6 bp and -3 bp insertion / deletion were detected in XY individual #3, leading to a frame shift. Targeted sequences are highlighted in the green line. PAM sequence is in red. Off-target mutations in *amhr2a* was not detected. Gonad of *amhr2bY-*knockout negative individuals in XY (D) and XX (E). Number of germ cells in XX gonads were larger than XY. Arrowheads in B indicate germ cells during mitosis. Asterisk in C indicates ovarian cavity. Scale bar: 10 μm.

To obtain direct evidence that Y-linked *amhr2bY* is the sex determining gene of ayu, we examined the gonadal development of the loss-of-function mutants for *amhr2bY* using CRISPR/Cas9 system in G0 generation. CRISPR-mediated *amhr2bY* mutations led to a frame shift (Fig 8A–8C). In general, primordial germ cells in female gonad proliferate by mitosis and then begin meiotic development during early gonadal differentiation [50]. On the other hand, primordial germ cells enter mitotic arrest in male gonad during this developmental period [15]. In ayu, number of germ cells in female gonads was also larger than males, so that the size of female gonads was bigger than males during early gonadal development (Fig 8D and 8E). In *amhr2bY*-knockout XY individuals, gonads were differentiated to female-like gonad at 3 mpf (Fig 8A and 8B). At 6 mpf, gonads of *amhr2bY*-knockout XY individuals, were differentiated to ovaries (Fig 8C). Diplotene stage oocytes and ovarian cavity were observed. A total of 160 injected individuals were analyzed. 81 individuals were genetically male (XY). Mutations by CRISPR/Cas9 were detected in 31 individuals of them. Seven individuals with mutation were induced to male-to-female sex reversal. Mutations by CRISPR/Cas9 were only induced in *amhr2bY* and off-target mutations in *amhr2a* were not detected.

### Development of PCR-based genotyping method for genetic sex identification

In Japan, female ayu are more economically important than males as food. Therefore, there is a need for genetic sex identification of ayu in aquaculture. Previously developed AFLP markers were for locations outside the sex-determining scaffolds and were not completely linked to the sex phenotype. We developed a PCR-based genotyping method for genetic sex identification using *amhr2bY* and *amhr2a* in ayu. Two bands corresponding to *amhr2bY* and autosomal *amhr2a* were amplified from genetic males, and one band of autosomal *amhr2a* was amplified from genetic females (S30 Fig). To confirm the accuracy of genotyping method by *amhr2bY*, we genotyped ayu from various populations and analyzed the correlation between genetic sex and phenotypic sex. A total of 387 males and 324 females were genotyped, and genetic sex and phenotypic sex matched in more than 99% of ayu individuals (S11 Table). Three XY female individuals and one XX male individual were observed, suggesting that sex reversal by environmental factors occurred in the ayu populations. Our genotyping method can be applied in ayu aquaculture for all-female production. In ayu, XX males can be produced by artificial sex hormone treatment [55]. All-female population can produce by mating XX females and XX males. Thus, this method will contribute to the development of the aquaculture industry.

## Discussion

### Ayu genome evolution

In this study, we obtained a draft genome sequence for ayu and analyzed syntenic relationships between ayu and other teleost fish. Ayu belongs to the superorder Stomiati and the order Osmeriformes, and Stomiati is phylogenetically classified as sister group of the superorder Neoteleostei [19–22]. The ayu genome size was estimated to be approximately 420 Mb by genome assembly and nuclear DNA staining. Genome size was estimated approximately 800 Mb in medaka and approximately 900 Mb in northern pike [32,56]. Our results indicated that genome compaction has occurred in the ayu lineage. In our annotation of the ayu genome, the density of predicted genes was 71.7 coding sequences/Mb, higher than that in medaka (32.2 genes/Mb) and northern pike (26.5 genes/Mb). This suggests that the ayu genome has compacted *via* reductions in the lengths of intergenic and intronic sequences, as occurred in the fugu genome [57]. Rainbow smelt (*Osmerus mordax*), a closely related species that also belongs to the order Osmeriformes and the family Osmeridae, has an estimated genome size of 690 Mb [58]. These results suggest that ayu genome compaction occurred after the divergence of the common ancestor of ayu and rainbow smelt. Of course, it is also possible that genome compaction occurred in a common ancestor and then the size of the rainbow smelt genome increased. Therefore, comparative genome analyses with other smelt fish in the order Osmeriformes will be useful to shed light on genome compaction in ayu.

The genetic linkage map of ayu contained 28 large LGs. The karyotype of the common teleost ancestor after TGD was 24, and medaka has retained this ancestral karyotype [32]. Comparative genomic analyses of medaka and ayu showed that 19 LG pairs retained one-to-one relationships, but four chromosome fissions and fusion of a pair of chromosomes have occurred as major chromosomal rearrangements. Thus, fission and fusion of ancestral chromosomes may have contributed to chromosomal rearrangements in ayu (Fig 1A and S6A and S7A Figs). Northern pike is phylogenetically classified as sister group of salmonids. The common ancestor of northern pike and Salmonidae diverged before the SaGD event [11,19,21,22]. Conserved synteny analysis of ayu and northern pike showed that 22 LG pairs retain one-to-one relationships with some minor translocations. In addition, one ayu to two northern pike relationships were detected as major chromosomal rearrangements (Fig 1B, S6B and S7B Figs). Ayu LG Pal8 and Pal25 corresponded to northern pike Elu13, Pal12 and Pal15 corresponded to Elu14, and Pal17 and Pal19 corresponded to Elu7. These three one-to-two relationships were also detected in the comparison between ayu and medaka. These results suggest that these chromosome fissions are ayu-lineage specific and occurred after the divergence of ayu and northern pike. In comparative genomic analyses of ayu and salmonids, 23 LG pairs between ayu and rainbow trout showed the expected one-to-two relationships and 22 LG pairs between ayu and Atlantic salmon also showed one-to-two relationships (Fig 1C and 1D and S7C and S7D Fig). However, some LG pairs showed one ayu-to-multiple salmonid chromosome relationships, indicative of fission and translocation of ancestral chromosomes. Thus, comparative genomic analyses among ayu, medaka and Protacanthopterygii indicate that the features of the ayu genome differ from those of Protacanthopterygii genomes. Ayu-specific chromosomal rearrangements are considered to reflect the phylogenic position of Stomiati, which is sister taxa of Neoteleostei. European smelt, *Osmerus eperlanus* belongs to the order Osmeriformes, and its karyotype is 2n = 56, the same as that of ayu [59]. Synteny analyses using other smelt fish are important to determine whether the chromosome rearrangements found in ayu are ayu- specific or common features in the superorder Stomiati. Previously, Osmeriformes was phylogenetically classified into superorder Protacanthopterygii. Ayu and salmonid have much in common in their external morphology such as adipose fin and life cycle such as migration between fresh water and sea and adaptation for cold water. However, the haploid chromosome number of ayu is 28, northern pike is 25 and Atlantic salmon and rainbow trout are 29. Few studies have investigated the genome evolution from a common ancestor to Stomiati and Protacanthopterygii to explain the difference of karyotype of these species. We provided first evidence that Stomiati-specific chromosomal rearrangements.

### Association mapping to identify ayu sex-determining locus

We conducted a genome-wide association study using wild ayu populations to detect sex-associated SNPs with genome-wide significance. These SNPs belonged to three scaffolds that were not anchored to linkage maps. Of the three sex-linked scaffolds, scaffold C showed the most significant association with sex in a scaffold-wide association test. We found *amhr2* located at positions 398–403 kb in male-specific regions of scaffold C. Another candidate, *eaa5b* (excitatory amino acid transporter 5b, as known as *slc1a7b* (solute carrier family 1 member 7b)), was also located in a Y-specific region around *amhr2* on a male-linked scaffold and was expressed in testes but not in ovaries. *eaa5* encodes a sodium-dependent glutamate transporter that is mainly expressed in axon terminals of rod and cone photoreceptors in the retina in mammals [60,61]. There are no previous reports that *eaa5* plays a role in gonadal sex determination/differentiation. Among the other candidate coding sequences, none have been reported to be involved in gonadal sex determination/differentiation. Thus, we concluded that *amhr2* on the Y chromosome is a strong candidate for the ayu sex-determining gene.

### Duplication of *amhr2* in ayu

We cloned two *amhr2* genes from ayu. *amhr2bY* was located at the male-specific sex-determining locus and was expressed in the testis, while *amhr2a* was located on autosomal LG Pal26, and was expressed in both the testis and ovary. A pairwise alignment of *amhr2Y* and autosomal *amhr2* with 5′ and 3′ putative regulatory regions and prediction of transcription factor binding sites in 5′ upstream regions revealed similarities only in the exon regions. No similarities were detected in the upstream, downstream and intron regions.

Conserved synteny analysis revealed that *amhr2* contained a genomic region derived from proto-chromosome 3 of the common ancestor of teleost fish before the TGD event. Proto-chromosome 3 duplicated and became the orthologous chromosome Ola5 in medaka, Pal20 in ayu, Elu17 in northern pike, Omy17 and Omy7 in rainbow trout, and Ssa12 and Ssa22 in Atlantic salmon and Ola7, Pal21, Elu12, Omy9, Omy16, Ssa13 and Ssa15 in the TGD event. It is considered that the common ancestor of medaka and ayu after the TGD event had two *amhr2* genes. The medaka lineage retained the orthologous chromosome Ola7 containing *amhr2* and lost Ola5. Ayu and the Protacanthopterygii lineage retained the orthologous chromosome Ola5 containing *amhr2*. Moreover, the *amhr2-*containing region was translocated from Pal20 to Pal26 in the ayu lineage. This translocation was not detected in northern pike and Salmonidae, suggesting that translocation of the *amhr2a*-containing region in ayu occurred after the divergence between the ayu and the Salmoniformes/Esociformes lineage.

Syntenic blocks surrounding *amhr2bY* on the sex-linked scaffold were evolutionarily conserved with Elu8, Omy1, Omy5, Ssa10, Ssa16 and Ola4. These were orthologs of ayu LGs Pal11 and Pal28. Pal11 had the highest linkage disequilibrium among the LGs in ayu, suggesting that sex-linked scaffolds may be mapped to Pal11. The highest linkage disequilibrium in Pal11 may reflect recombination suppression between the X and Y chromosomes. The region surrounding *amhr2bY* in ayu was evolutionarily conserved with regions in northern pike, rainbow trout, Atlantic salmon and medaka, while *amhr2bY* was detected only in ayu and not in other teleost fish. In addition, comparisons of the regulatory regions of *amhr2bY* and *amhr2a* could not detect similarity. More putative transposable elements were detected around *amhr2bY* than the average number detected across the whole genome (2-sample test for equality of proportions *p* < 0.001). These results raise the hypothesis that *amhr2bY* duplicated by itself. Of course, there are a possibility that *amhr2bY* duplicated with some surrounding genes and these surrounding genes have been lost. *Amhr2bY* obtained its function as a sex-determining gene as a result of autosomal *amhr2a* duplication and translocation. It is considered that transposable elements around the paralog of *amhr2* on the Y chromosome triggered translocation. It is assumed that *amhr2bY* translocated with some of the surrounding region containing existing gonad-specific regulatory elements to a new position during the *amhr2* duplication-transposition event, because *amhr2bY* was expressed in testicular supporting cells. One hypothesis is that the Y copy of *amhr2* acquired novel regulation at its new location. The *amhr2* duplication-transposition event likely occurred after divergence from the common ancestor of ayu, northern pike and Salmonidae. This type of evolution of a sex-determining gene by gene duplication-transposition has also been reported for other teleosts, such as medaka, Patagonian pejerrey, northern pike, yellow perch and threespine stickleback [4,5,10–12,14]. Our results raise the question as to whether translocation of the *amhr2a*-containing region from Pal20 to Pal26 and the duplication-transposition event of *amhr2a* to the Y-specific region were independent or linked events. Conserved syntenic analyses and association mapping to detect sex-linked loci in other smelt fish in the order Osmeriformes may resolve this question.

### Models for the mechanism of sex determination in ayu

Müllerian-inhibiting hormone, or AMH, was first identified in mammals [62,63]. AMH belongs to the transforming growth factor beta (TGF-β) superfamily and induces degeneration of Müllerian ducts, which are female reproductive ducts of tetrapods, in male embryos during early gonadal development. AMH transmits signals by binding to AMHR2, a type II transmembrane receptor for members of the TGF-ß family [64].

In teleosts, *amh*/*TGF-β* signaling is also critical for sex determination, even though fish do not have Müllerian ducts [64]. In Patagonian pejerrey, northern pike and threespine stickleback, the sex-determining gene is *amh* on the Y chromosome [10,11,12]. In *Oryzias luzonensis*, another TGF-β, *gsdfY* (gonadal soma-derived factor on the Y chromosome) is the sex-determining gene [6]. The *gsdf* gene is a critical factor for male sex differentiation in many teleosts [65] and is one of the direct downstream targets of the sex-determining gene *dmy* in medaka [66]. In yellow perch, the Y-linked duplicate of *amhr2* is a strong candidate for the sex-determining gene. In addition, the Y-linked *amhr2* has a specific deletion in its extracellular domain, suggesting that Y-linked *amhr2* and autosomal *amhr2* have different functions [14]. In fugu, *amhr2* was located on both Y chromosome and X chromosome and expressed in somatic cells of undifferentiated gonads in both sexes. Genetic sex is determined by an *amhr2* paralog containing a missense SNP in its kinase domain that reduces its signaling activity [13]. Thus, we concluded that *amhr2bY* was more likely to be the sex-determining gene than the nine other genes located in regions containing strongly sex-linked SNPs.

Expression analyses indicated that *amhr2bY* mRNA expression was male-specific in somatic cells sounding germ cells in morphologically undifferentiated gonads, indicating that *amhr2bY* was expressed in supporting cells. In other teleosts, sex-determining genes are expressed in supporting cells of undifferentiated gonads [4,6,10]. In mammals, the Y-linked sex-determining gene *Sry* induces testis development by directing sexually undifferentiated supporting cells to develop as Sertoli cells, which then direct all other gonadal cell types, including germ cells, to secondarily initiate a male differentiation pathway [67,68]. Thus, the expression pattern of *amhr2bY* in ayu corresponded well with those of testis-determining genes in other vertebrates. Furthermore, loss-of-function mutation of *amhr2bY* induced male to female sex reversal, indicating that *amhr2bY* is critical for testicular development in ayu. Our results indicate that Y-linked *amhr2bY* is the sex-determining gene in ayu, and male-specific expression of *amhr2bY* determines the genetic sex of ayu.

**Fig 9:**
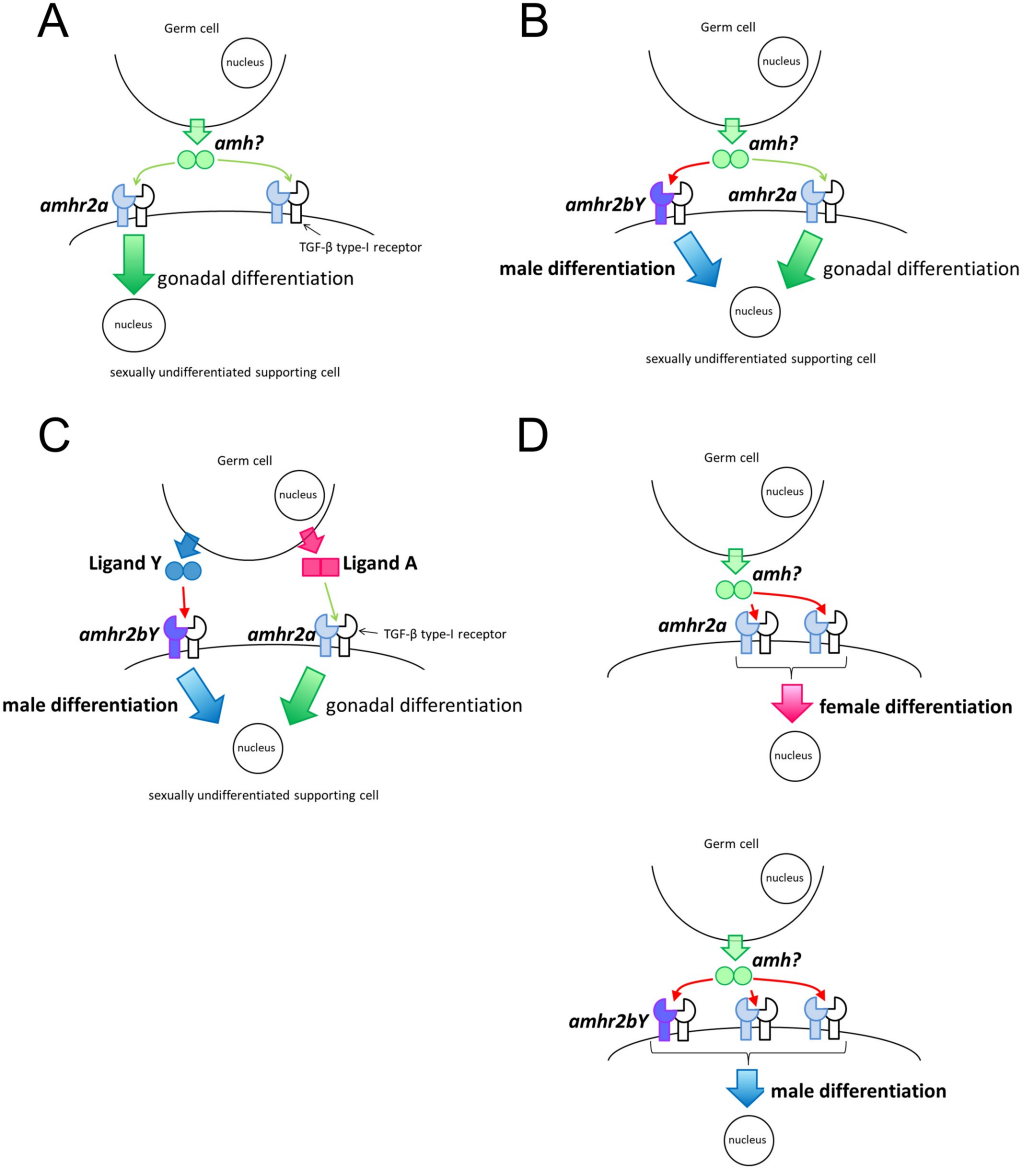
Summary diagram of gonadal sex determination in ayu. (A) Autosomal Amhr2a is located in supporting cells surrounding germ cells in sexually undifferentiated gonads. Putative ligand Amh is secreted from germ cells and supporting cells. amh/amhr2 signaling plays a role in germ cell–supporting cell interaction. (B) Hypothesis that Amhr2bY and Amhr2a have different signaling pathways. The same ligand binds to both Amhr2bY and Amhr2a. Amhr2bY initiates testicular development via a male-specific signaling pathway in sexually undifferentiated supporting cells surrounding germ cells. (C) Hypothesis that different ligands bind to Amhr2bY and Amhr2a. Amhr2bY binds to amhr2bY-specific ligand (ligand Y). Amhr2bY is activated by a specific ligand and initiates testicular differentiation. Amhr2a binds to amhr2a-specific ligand (ligand A) and functions in both male and female gonadal development. (D) Hypothesis that gene dosage of Amhr2s determines genetic sex in ayu.

In medaka, *amhr2* signaling plays an important role in germ cell–supporting cell interactions during early gonadal differentiation [69]. In ayu, transcripts of *amh*, the putative ligand of *amhr2bY* and autosomal *amhr2a*, were detected in both supporting cells and germ cells in morphologically undifferentiated gonads of both males and females. Autosomal *amhr2a* transcripts were detected in somatic cells of sexually undifferentiated gonads in both genetic males and females, suggesting that *amh-amhr2* signaling functions in germ cell–supporting cell interactions and supporting cell–supporting cell interactions during early gonadal development, regardless of sex (Fig 9A).

In ayu, *amhr2bY* was expressed in autosomal *amhr2a*-expressing cells. Several models can be considered to explain how *amhr2bY* determines the genetic sex, when *amhr2bY* and *amhr2a* are expressed in the same cells simultaneously during early gonadal development. In the evolutionarily conserved ATP-binding site in the catalytic domain of serine/threonine kinases in the BMPR2 and AMHR2 family, the 267^th^ amino acid in Amhr2bY is glutamine (a polar amino acid), while hydrophobic amino acids are located at the same position in Amhr2 proteins of other teleosts, including Amhr2a. Four contiguous amino acids including Q267 (265–268^th^ amino acids) are evolutionarily conserved as the ATP-binding site in BMPR2 and AMHR2 family kinases in vertebrates. In human, a missense mutation at H282Q (equivalent to amino acid 268 in ayu Amhr2bY) is associated with the persistent Müllerian duct syndrome, which is characterized by the lack of regression of Müllerian derivatives in externally phenotypic males [70]. Thus, the kinase activity of Amhr2bY may differ from those of Amhr2a and Amhr2s of other teleosts. If both receptor genes bind to the same ligand, then the downstream signaling pathway may differ between Amhr2bY and Amhr2a, because Amhr2bY has a mutation in the kinase domain. Our results suggest that *amhr2bY* determines the genetic sex and initiates testicular development *via* a male-specific *amhr2* signaling pathway in sexually undifferentiated supporting cells (Fig 9B). However, the extracellular domain of Amhr2bY contains some specific amino acid changes, such as S44 (glycine to serine), I102 (polar to hydrophobic) and N131 (hydrophobic to polar). This raises the possibility that Amhr2bY and Amhr2a bind to different ligands. In this model, Amhr2bY binds to an *amhr2bY*-specific ligand. Then, Amhr2bY and the specific ligand complex transmit the male differentiation signal and initiate testicular development. Amhr2a binds to the *amhr2a*-specific ligand and functions in both male and female gonads (Fig 9C). Another hypothesis is that the gene dosage of *Amhr2s* is critical for sex determination in ayu, if Amhr2bY and Amhr2a bind to the same ligand and have exactly the same function. In that case, the 2X gene dosage of *amhr2* would induce ovarian differentiation, whereas the 3X gene dosage (2 autosomes + Y chromosome) would induce testicular differentiation (Fig 9D). Further studies are required to investigate the details of the signaling pathway(s) *via* Amhr2bY and autosomal Amhr2a during early gonad differentiation to reveal the molecular mechanisms of sex determination in ayu. While amh/TGF-β signaling is critical for sex determination in various teleost fish, little is known about the difference in function between Y-linked *amhr2* and autosomal *amhr2*. Based on genetic evidence and expression patterns, we presented the first models of sex determination by duplicated *amhr2*.

In summary, we sequenced the genome of ayu (*P*. *altivelis*) as a model species in the superorder Stomiati. Conserved synteny analysis between ayu, Neoteleostei and Protacanthopterygii indicated that the ayu genome has undergone specific chromosomal rearrangements, which appear to have occurred after the divergence of ayu and the Protacanthopterygii lineage. Genome-wide association mapping using wild populations identified sex-linked scaffolds and a duplicate copy of *amhr2* located in one sex-linked scaffold. Y-linked *amhr2* expression was male-specific in supporting cells surrounding the germ cells of undifferentiated gonads. Loss-of-function analysis for *amhr2bY* indicated that *amhr2bY* is critical for gonadal sex determination ayu. These results indicate that *amhr2* on the Y chromosome is the sex-determining gene of ayu.

## Materials and methods

### Ethics statement

All animal experiments were carried out with the approval of the Institutional Animal Care and Use Committee of the Tokyo University of Marine Science and Technology (No. 92, H30-13).

### Ayu sources

The draft genome sequence of ayu was obtained from one male individual captured from a wild population in the Ota River, Hiroshima, Japan (34°21′39″N 132°24′20″E). A single F_1_ full-sib family (90 siblings and both parents) reared at Tokyo University of Marine Science and Technology derived from the Ota River population was used to construct the ayu genetic linkage maps. In total, 24 males and 24 females captured from a wild population in the Edogawa River (Matsudo city, Chiba, Japan, 35°46’36"N 139°53’28"E) were used for GBS analysis to map sex-determining loci. Ten males and nine females captured from a wild population in the Tama River (Ota ward, Tokyo, Japan, 35°33’48"N 139°40’43"E) were used for resequencing to map sex-determining loci. To confirm sex-linked scaffolds detected by GWAS, two F_1_ full-sib family (88 siblings, two male parents and two female parents) were used for linkage analysis using GRAS-Di analysis. Parents of these families were captured from a wild population in the Nagaragawa River (Kakamigahara city, Gifu, Japan, E136°51′N35°24′) and siblings were mixed and reared until the phenotypic sex could be determined. 161 males and 143 females from cultured strain derived from Ota River, 98 males and 119 females captured from a wild population in the Nagaragawa River, 43 males and 23 females captured from a wild population in the Tama River, 74 males and 31 females captured from a wild population in the Jinzukawa River (Toyama city, Toyama, Japan, E137°13′N36°42′) and 11 males and 8 females cultured fish originated from Biwa Lake (Shiga, Japan, E135°56’N35°07’) were used to develop PCR-based genotyping method for genetic sex. Phenotypic sex was identified based on gross morphology of gonads. The sampling locations were summarized in S8 Fig.

### Construction of reference genome sequence

The ayu genome size was estimated by the standard traditional nuclear DNA staining method with 4′,6-diamidino-2-phenylindole (DAPI) using red blood cells [71]. Tiger pufferfish (*Takifugu rubripes*), medaka (*O*. *latipes*) and zebrafish (*D*. *rerio*) were used as standards. Data are means of triplicate experiments for standards and duplicate experiments for ayu. Genomic DNA was extracted from the fin tissue of one male ayu using a standard phenol/chloroform protocol. Short-read DNA sequencing was carried out on the Illumina HiSeq2000 platform by Eurofins Genomics (Tokyo, Japan) with 300-bp, 3-kb, 8-kb, 20-kb, and 40-kb libraries using 100-bp paired-end sequencing. For long-read sequencing, genomic DNA was also sequenced on the PacBio RSII platform by Macrogen Japan (Kyoto, Japan). Sequencing data have been deposited in the DDBJ Sequence Read Archive (DRA) under the accession number DRA010912. Low-quality bases of raw Illumina short reads were removed using Trimmomatic v3.6 (shotgun library: ILLUMINACLIP:TruSeq3-PE.fa:2:30:10 LEADING:20 TRAILING:20 SLIDINGWINDOW:5:20 MINLEN:50; jumping library: LEADING:20 TRAILING:20 SLIDINGWINDOW:4:15 MINLEN:30) [72]. Then, trimmed short reads were *de novo* assembled using the Platanus v1.2.4 genome assembler with K-mer = 42 [73]. Short-read scaffolds were scaffolded using long reads by SSPACE-LongRead [74]. Gaps within the genome assembly were filled with short reads using GapFiller v1.10 [75] and with long reads using LR_Gapcloser v1.0 (http://www.fishbrowser.org/software/LR_Gapcloser). PacBio long reads were *de novo* assembled by Canu v1.4 and hybrid assembled with Platanus’s contigs by DBG2OLC [76,77]. The assemblies were merged with quickmerge [78]. Finally, short- and long-read assemblies were integrated by Metassembler v1.5 [79] and polished by Pilon v1.22 [80].

### Genetic linkage map construction

Genomic DNA was extracted from the caudal fins of the ayu F_1_ full-sib family (90 siblings and parents) using a DNeasy Blood and Tissue kit (Qiagen, Hilden, Germany) according to the manufacturer’s instructions. DNA libraries were prepared using the restriction enzyme ApeKI according to the GBS protocol [81]. For GBS libraries, 96 samples (90 siblings, triplicate of male and female parents) were sequenced per lane on the Illumina HiSeq4000 platform with 100-bp paired-end reads.

Library construction and sequencing were carried out by BGI (Shenzhen, China). Sequencing data have been deposited in the DRA under the accession number DRA010913. Low-quality reads were trimmed by Trimmomatic v3.6 [72]. Trimmed reads were mapped to the ayu draft genome sequence by bwa mem v0.7.12 [82]. Variant calling was performed in GATK HaplotypeCaller v4.0.5 according to GATK best practice recommendations [83]. Raw variants were filtered with VCFtools v0.1.16 (parameters:--minQ 100--minDP 5--maxDP 500 –min-meanDP 15--max-missing 0.95--remove-indels--maf 0.01--hwe 0.0001--thin 500) [84]. Then, SNPs with the segregation patterns AA × AB, BB × AB and AB × AB were identified.

Genetic linkage maps were constructed with the pseudo-testcross strategy using F_1_ full-sib family in Lep-Map3 [27,85]. Three genetic linkage (female-specific map that constructed female parent informative markers and both parents informative markers, male-specific map that constructed male parent informative markers and both parents informative markers, and consensus sex-averaged linkage map) maps were constructed. The Filtering2 module in Lep-map3 was used to filter out markers with significant segregation distortion (dataTolerance = 0.001). The LGs were separated using the SeparateChromosomes2 module with a logarithm of odds (LOD) threshold of 10 (lodLimit parameter) and a recombination rate of 0.03 (theta parameter). The JoinSingles2All module was then used to assign additional single SNPs to existing LGs using an LOD threshold of 3. The LGs were ordered using the OrderMarkers2 module with five iterations. Maternal and paternal linkage maps were constructed separately. The minimum number of markers per LG was set to 10. A sex-averaged linkage map was also obtained using Lep-map3 software. Linkage maps were confirmed by the position of SNP markers in the ayu genome sequence and conserved synteny relationship between other teleost genomes. Linkage map summaries were visualized using the R package LinkageMapView [86]. Then, the ayu reference genome was ordered and oriented based on genetic linkage maps using ALLMAPS [29]. The female linkage map was assigned a weight of 2, and the male map a weight of 1. Chimeric scaffolds that mapped to different LGs were split using ALLMAPS. The split threshold was set to at least two markers.

Genome assembly completeness was evaluated using BUSCO v3.0.1 with actinopterygii_odb9 [30]. Repetitive elements were detected in RepeatMasker v4.0.5 using fugu and zebrafish repeat databases (http://www.repeatmasker.org) [87]. Gene prediction was carried out in BRAKER v2.0.4 with Augustus v3.3.3 and GeneMark-ES v4.57 trained using RNAseq data from ayu [20,31,88–90]. Predicted genes were blasted against the UniProt database with an e-value < 10^−5^ and annotated with GO terms using Blast2GO v5 [91,92].

### Conserved synteny analysis

Conserved synteny analysis between ayu and the genomes of other teleosts, including Northern pike (*E*. *lucius*), rainbow trout (*O*. *mykiss*), Atlantic salmon (*S*. *salar*), medaka (*O*. *latipes*), zebrafish (*D*. *rerio*) and spotted gar (*L*. *oculatus*) were performed using Synima [93]. Orthologous genes were clustered by reciprocal best hits using predicted protein sequences. The minimum number of paired genes in a synteny block was set to 3. For conserved synteny analysis of the *amhr2bY*-containing region, putative orthologous gene pairs were complemented by blast searches manually. Syntenic relationships were visualized by Circos v0.69 and VGSC2 [94,95]. The northern pike, rainbow trout and Atlantic salmon genome sequences were obtained from the NCBI (accession numbers GCF_004634155, GCF_002163495 and GCF_000233375, respectively). The genome sequences of medaka, zebrafish and spotted gar were obtained from Ensembl Release 98 [96].

### Genome-wide association study to identify sex-determining locus

Genomic DNA was extracted from the caudal fins of 24 males and 24 females captured from the wild population in the Edogawa River using a DNeasy Blood and Tissue kit (Qiagen) according to the manufacturer’s instructions, and then used for GBS analysis [81]. For GBS libraries, 48 samples were sequenced per 1/2 lane on the Illumina HiSeq4000 platform with 100-bp paired-end reads. Sequencing data have been deposited in the DRA under the accession number DRA010914.

For whole-genome resequencing, genomic DNA was extracted from the caudal fins of 10 males and nine females captured from the wild population in the Tama River using a Blood and Cell Culture DNA Mini Kit (Qiagen) according to the manufacturer’s instructions and then sequenced on the Illumina HiSeq X platform with 150-bp paired-end reads at approximately 12× coverage. Library construction and sequencing were carried out by BGI (Shenzhen, China). Sequencing data have been deposited at the DRA under the accession number DRA010915. Low-quality reads were trimmed by Trimmomatic v3.6 [72]. Trimmed reads were mapped to the ayu reference genome sequence by bwa mem v0.7.12 [82]. For resequencing data, duplicate reads were marked using Picard tools v2.1.18 (http://broadinstitute.github.io/picard/). Mapping coverage was calculated by deepTools v3.1.3 [97] and visualized with Integrative Genomics Viewer v2.3.68 [98]. Variants between individuals from the Edogawa River and the Tama River were simultaneously called with GATK HaplotypeCaller v4.0.5 [83]. The SNPs located in repetitive sequences detected by RepeatMasker were eliminated from analyses.

For the genome-wide association scan, raw variants from GBS and resequencing data were filtered in VCFtools (criteria:--minQ 100,--minDP 5,--maxDP 500,–min-meanDP 8,--max-missing 0.75,--maf 0.05,--hwe 0.0001, and--thin 500) [84]. In total, 25,118 SNP markers were retained (S9A Fig). Pearson’s squared correlation coefficient (r^2^) values were calculated using plink v1.9 (parameters:--r2,--ld-window 50,--ld-window-kb 10000 and--ld-window-r2 0) to estimate the linkage disequilibrium between each pair of SNPs on each LG [99]. The PCA based on these markers was performed with plink v1.9. The PCA scores were clustered by the k-means clustering algorithm in the cluster package in R [100]. An association test for sex-linked loci was performed by GWASpoly with the “1-dom-ref” model (the simplex-dominant model for reference alleles) [44]. The genome-wide significance threshold was set to a Bonferroni-corrected *p*-value < 0.05 (3.26 × 10^−8^).

For the genomic scan of selective sweeps between males and females, raw variants detected by whole-genome resequencing were filtered using VCFtools (criteria:--minQ 100,--minDP 5,--maxDP 500,--max-missing-count 2,--maf 0.05, and--hwe 0.0001) [84]. In total, 444,151 SNPs were retained (S9B Fig). Weir and Cockerham-weighted *F*-statistics (Fst) and the DNA nucleotide diversity index (π) estimated 100-kb windows in 50-kb steps along the genome using VCFtools [84,101]. Scaffolds of < 50 kb and windows with < 10 SNPs were eliminated from analysis. Fst values were normalized by Z score transformation. The SNP density was visualized using the R package CMplot (https://github.com/YinLiLin/R-CMplot). Manhattan plots were created with the R package qqman [102].

### Linkage disequilibrium analysis of sex-determining locus

For linkage disequilibrium analysis of sex-associated scaffolds, pairwise r^2^ values between SNPs detected by whole-genome resequencing with minor allele frequencies > 20% were calculated and plotted with the R package LDheatmap [103]. Sex-associated scaffold association tests were performed using plink v1.9 with the allelic model using Fisher’s exact test. The scaffold-wide significance threshold was set to a Bonferroni-corrected *p*-value < 0.05.

### Molecular cloning and expression analysis of candidate sex-determining gene of ayu

Total RNA was extracted from testes and ovaries at approximately 12 mpf from ayu from the Tama River population using an RNeasy Mini kit (Qiagen). The extracted RNA was used for first-strand cDNA synthesis with an Omniscript RT kit (Qiagen) with oligo-dT primers. The cDNA fragments of ayu *amhr2bY* and *amhr2a* were amplified by RT-PCR using PrimeSTAR HS DNA Polymerase (Takara Bio Inc., Otsu, Japan), with amhr2bY-1F and amhr2bY-2R primers to amplify *amhr2bY* and amhr2a-1F and amhr2a-2R primers to amplify *amhr2a*. These primers were designed based on the predicted coding sequence of each gene (S12 Table). The PCR conditions were as follows: 1 min at 95°C, followed by 35 cycles of 10 s at 98°C, 5 s at 55°C and 2 min at 72°C, with final extension for 5 min at 72°C. The amplified fragments were cloned into a pGEM-T easy vector (Promega, Fitchburg, WI, USA) for sequencing of both strands on an Applied Biosystems 3730xl DNA Analyzer (Thermo Fisher Scientific, Waltham, MA, USA).

To obtain the full-length cDNA of the ayu candidate sex-determining gene, we carried out 5′ and 3′ RACE. SMART cDNA was synthesized using a SMART RACE cDNA Amplification kit (Takara Bio Inc.) according to the manufacturer’s instructions with the gene-specific primers amhr2bY-3R for 5′ RACE of *amhr2bY*, amhr2bY-5F for 3′ RACE of *amhr2bY*, amhr2a-3R for 5′ RACE of *amhr2a* and amhr2a-4F for 3′ RACE of *amhr2a* (S12 Table). The PCR amplification conditions were as follows: 1 min at 95°C; followed by five cycles of 10 s at 98°C and 2 min at 72°C; five cycles of 10 s at 98°C, 5 s at 70°C, and 2 min at 72°C; five cycles of 10 s at 98°C, 5 s at 68°C, and 2 min at 72°C; 28 cycles of 10 s at 98°C, 5 s at 65°C, and 2 min at 72°C; and final extension for 5 min at 72°C. For *amhr2bY*, nested PCR was performed using 1 μL of the product of the first PCR round, the gene-specific primers amhr2bY-4R and amhr2bY-6F, and the following cycling protocol: 1 min at 95°C, followed by 35 cycles of 10 s at 98°C, 5 s at 65°C, and 2 min at 72°C, with final extension for 5 min at 72°C (S12 Table). The 5′ and 3′ RACE products were cloned into a pGEM-T easy vector (Promega) for sequencing. The 15-amino acid insertion in *amhr2a* was confirmed by RT-PCR and sequencing with amhr2a-7F and amhr2a-8R using different individuals from those used to clone full-length cDNA (S12 Table). To confirm that *amhr2bY* is Y chromosome-specific, the *amhr2bY* genomic region was amplified with the primers amhr2bY-1F and amhr2bY-2R from 12 males and 12 females from the Tama River population. These individuals were different from the ten males and nine females from the Tama River used in the association study.

For expression analyses, testes and ovaries of immature individuals from cultured ayu derived from the Ota River (*n* = 4 of each sex) were dissected and fixed in RNAlater solution (Qiagen). Total RNA was extracted using an RNeasy Mini kit (Qiagen). First-strand cDNA was synthesized using an Ominiscript RT kit (Qiagen) and amplified by Takara Ex Taq (Takara Bio Inc.) using the primer sets amhr2bY-1F and 2R, and amhr2a-1F and 2R. The PCR conditions were as follows: 1 min at 95°C, followed by 35 cycles of 30 s at 95°C, 30 s at 55°C, and 2 min at 72°C. As an internal control, β-actin mRNA was amplified using the primer set bact-F and bact-R under the same PCR conditions mentioned above, but with 28 cycles (S12 Table). The conditions for genomic PCR and RT-PCR for other candidate coding sequences, including primer sequences and thermal cycling conditions, are summarized in S13 Table. The PCR products were sequenced using the same primers.

The protein structures of Amhr2bY and Amhr2a were predicted using the SWISS-MODEL homology-modelling server [48]. The crystal structure of the kinase domain of human BMPR2 (PDB ID; 3g2f.1.A [104]) was used as the template for homology modelling. Predicted models were visualized in UCSF Chimera v1.14 [105].

To compare genomic sequences of the putative regulatory regions for *amhr2bY* and *amhr2a*, we performed pairwise genome alignments of the 30-kb genomic sequence around *amhr2bY* and *amhr2a* (approximately 10 kb of the 5′ upstream region, the regions containing *amhr2bY* and *amhr2a*, and approximately 10 kb of the 3′ downstream region) using lastz v1.03.72 (https://github.com/lastz/lastz) [106]. Putative transcription factor binding sites in the intergenic regions between amhr2s and their adjacent genes were predicted by CiiiDER v0.9 using the JASPAR2020 CORE vertebrates non-redundant database [107,108].

### Molecular phylogenetic analysis

The deduced amino acid sequences of Amhr2bY and Amhr2a in ayu were aligned with amino acid sequences of Amhr2 proteins from other teleosts using Muscle in MEGA X [109]. Multiple alignments were visualized by Jalview with the ClustalX color scheme [110]. A phylogenetic tree of teleost AMHR2s was constructed in MEGA X using the maximum likelihood method. Bootstrap resampling was repeated 10,000 times. The names and GenBank accession numbers of analyzed sequences are shown in S14 Table.

### Development of PCR-based genotyping method for genetic sex identification

Genomic DNA was extracted from caudal fins using a DNeasy Blood and Tissue kit (Qiagen) according to the manufacturer’s instructions. Genomic DNA was amplified by Takara Ex Taq (Takara Bio Inc.). The primer set Ayu-sex-1F and Ayu-sex-2R was designed based on sequences of exon1 and exon2 (S12 Table). The primer set Ayu-sex-3F and Ayu-sex-4R was designed based on sequences of exon8 and exon9 (S12 Table). The intron length of *amhr2a* was longer than that of *amhr2bY* and these amplicons were able to be separated by agarose gel electrophoresis. The PCR conditions were as follows: 1 min at 95°C, followed by 30 cycles of 30 s at 95°C, 30 s at 55°C, and 1 min at 72°C, with a final extension of 5 min at 72°C. DNA fragments were separated by agarose gel electrophoresis using 1% (w/v) Agarose S (Nippon Gene, Toyama, Japan). Genetic males had two amplified bands at 872 bp and 1162 bp that were derived from *amhr2bY* and autosomal *amhr2a*, whereas genetic females had one amplified band at 1162 bp that was derived from *amhr2a* by Ayu-sex-1F and Ayu-sex-2R (S30A Fig). Likewise, genetic males had two amplified bands at 390 bp and 960 bp, whereas genetic females had one band at 960 bp amplified using Ayu-sex-3F and Ayu-sex-4R (S30B Fig). To confirm accuracy of genotyping method by *amhr2bY*, ayu from various population (cultured strain derived from Ota River, wild ayu in the Tama River, Nagaragawa Rive and Jinzukawa River and cultured fish originated from Biwa Lake) were genotyped using Ayu-sex-3F and Ayu-sex-4R.

### Expression analysis of candidate sex-determining gene by *in situ* hybridization

Ayu larvae at 2 mpf were fixed in 4% paraformaldehyde/1× PBS (Wako Pure Chemical Industries, Ltd, Osaka, Japan) at 4°C overnight. Genomic sex was identified by genomic PCR using the primer set Ayu-sex-1F and Ayu-sex-2R (S12 Table). Tissues were dehydrated and embedded in Paraplast Plus tissue embedding medium (McCormick Scientific, St. Louis, MO, USA), and then serial 5-μm sections were cut with a microtome. Sense and antisense digoxigenin (DIG)- and fluorescein isothiocyanate (FITC)-labeled cRNA probes were generated by *in vitro* transcription with a DIG RNA labeling kit (Roche Diagnostics, Mannheim, Germany). The amplified genes/genetic regions and their corresponding PCR primers were as follows: full-length *amhr2bY*, amhr2bY-7F and amhr2bY-8R; 3′ UTR of *amhr2bY*, amhr2bY-9F and amhr2bY-10R; 3′ UTR of *amhr2a*, amhr2a-9F and amhr2a-10R; *ddx4*, ddx4-F and ddx4-R; *sox9b*, sox9b-F and sox9b-R; and *amh*, amh-F and amh-R (S12 Table). The amplified cDNA fragment lengths of *ddx4*, *sox9b* and *amh* were 1,881 bp, 1,480 bp and 1,583 bp, respectively (GenBank accession numbers LC512013, LC512014 and LC512015). Tissue sections were deparaffinized, hydrated, and treated with 4 μg/ml proteinase K (Roche Diagnostics) at 37°C for 5 min, and then hybridized with sense or antisense RNA probes at 60°C for 24 h. Hybridization signals were detected using an alkaline phosphatase-conjugated anti-DIG antibody (Roche Diagnostics, 1/1,000 dilution) and NBT/BCIP (Roche Diagnostics) as chromogens. After staining, sections were washed in water and mounted in Glycergel mounting medium (Dako Inc., Via Real, Carpinteria, CA, USA). For two-color *in situ* hybridization, FITC- and DIG-labeled cRNA probes were detected with peroxidase-conjugated anti-DIG and -FITC antibodies (Roche Diagnostics, 1/500 dilution), respectively, and the TSA Plus Fluorescein/Cy3 system (PerkinElmer Co., Ltd, Waltham, MA, USA). Sections were counterstained with DAPI and mounted in Fluoromount (Diagnostic BioSystems Inc, Pleasanton, CA, USA). At least three individuals were prepared for each genetic sex.

### Loss-of-function analysis of *amhr2bY*

The sgRNA for *amhr2bY* was designed using CCTOP [111]. Forward 5’-TAGGAGTAAGGCTCGGACCAGT-3’ and reverse 5’-AAACACTGGTCCGAGCCTTACT-3’ oligo nucleotide for target sequences of *amhr2bY* were annealed and cloned into the BsaI site of the pDR274 vector (Addgene, Watertown, MA. catalog number: #42250). The sgRNA was transcribed using DraI-digested gRNA expression vectors as templates using the ScriptMAX Thermo T7 Transcription kit (TOYOBO). Then, sgRNA was treated DNase I and purified by RNeasy Mini Kit (Qiagen). The 150 ng of Cas9 Nuclease protein NLS (Nippon Gene, Tokyo, Japan) and 25 ng of sgRNA were microinjected into fertilized ayu one-cell eggs obtained from the cultured strain derived from the Ota River population using a microinjector BJ-110 (BEX, Tokyo, Japan). The Body of *amhr2bY-*targeted G0 ayu were fixed in Bouin’s solution and analyzed histology of developing gonads. The head and tail of *amhr2bY*-targeted G0 ayu were used to extract genomic DNA. Mutations of target site for CRISPR/Cas9 were identified by genomic PCR using the primer set amhr2bY-11F and amhr2bY-12R for *amhr2bY* and amhr2a-11F and amhr2a-12R for *amhr2a* (S12 Table). The PCR products were cloned into a pGEM-T easy vector (Promega) and analyzed by Sanger sequencing to identify individual editing patterns. At least 8 clones per individual were examined.

## Supporting information

S1 Fig Schematic figure for phylogenetic classification of species using conserved synteny analysis. TGD: Teleost-specific whole-genome duplication event. SaDG: Salmonid-specific whole-genome duplication even. Phylogenetic tree was illustrated to Hughes et al [22].
(JPG)

S2 Fig Characterization of ayu genome. (A) Estimation of ayu genome size by nuclear DNA staining. Calibration curve was constructed using nuclear DNA of tiger pufferfish, medaka and zebrafish. X-axis, fluorescence peaks; Y-axis, C-value (pg). (B) Comparison of predicted repetitive elements in ayu and other teleosts by RepeatMasker using fugu repeat databases. Repetitive elements accounted for 56.78 Mb (12.61% of the ayu genome assembly): 39.46 Mb (8.76%) were simple repeats, 12.05 Mb (2.67%) were low complexity repeats, 4.20 Mb (0.93%) were retroelements, and 0.96 Mb (0.21%) were DNA transposons. (C) Comparison of predicted repetitive elements in ayu and other teleosts by RepeatMasker using zebrafish repeat databases. Repetitive elements predicted for 65.15 Mb (14.47%): 29.60 Mb (6.57%) were simple repeats, 11.14 Mb (2.47%) were low complexity repeats, 8.65 Mb (1.92%) were retroelements, and 17.84 Mb (3.96%) were DNA transposons. Y-axis indicates length of repetitive elements.
(JPG)

S3 Fig Female linkage map of ayu. (JPG)

S4 Fig Male linkage map of ayu. (JPG)

S5 Fig Sex-averaged linkage map of ayu. (JPG)

S6 Fig Summary of orthologous chromosome segments. (A) Bar plot of pairwise syntenic blocks between medaka and ayu. (B) Bar plot of pairwise syntenic blocks between ayu, northern pike, rainbow trout and Atlantic salmon. Colors mark chromosomal location of conserved syntenic blocks. White indicates regions for which no information for syntenic blocks is available.
(JPG)

S7 Fig Synteny analysis between ayu genome and those of other teleost fishes. Oxford plot between ayu genome and (A) medaka, (B) northern pike, (C) rainbow trout, (D), Atlantic salmon, (E) zebrafish, and spotted gar (F). Numbers refer to pairs of orthologous genes clustered by reciprocal best hits algorithm. “Sex” in bottom row of each panel indicates sex-linked scaffolds detected by genome-wide association study, not only putative Y-specific regions.
(JPG)

S8 Fig Sampling locations of ayu. Map data was based on the Digital Map published by Geospatial Information Authority of Japan (https://maps.gsi.go.jp/).
(JPG)

S9 Fig Density of SNPs using genome-wide association scan. (A) Density of SNPs using genome-wide association scan in a 100-kb window size by genotyping-by-sequencing (GBS) and whole-genome resequencing. (B) Density of SNPs detected by whole-genome resequencing in a 100-kb window size.
(JPG)

S10 Fig Decay of linkage disequilibrium with physical distance in ayu wild population measured by r^2^ of SNPs using association mapping for sex-determining locus. X axis: physical distance between SNPs. Y axis: pairwise linkage disequilibrium (r^2^).
(JPG)

S11 Fig Principal component analysis (PCA) based on 25,118 SNP markers identified by genotyping-by-sequencing (GBS) and by whole-genome resequencing. (A) PCA scores plot pc1 versus pc2. (B) PCA scores plot pc3 versus pc4. (C) PCA scores plot pc5 versus pc6. (D–O) Subgroups with similar SNP distributions clustered by k-means clustering algorithm on pc1 versus pc2 (D, G, J and M), pc3 versus pc4 (E, H, K and N) and pc5 versus pc6 (F, I, L and O). Cluster number with *k* set to 2 (D–F), 3 (G–I), 4 (J–L) and 5 (M–O). Large markers indicate center of cluster. Edogawa River population based on GBS and Tama River population based on resequencing are separated on pc1. Edogawa River males and females are well separated on pc4, but Tama River sexes are not as well separated.
(JPG)

S12 Fig Quantile–quantile (QQ) plot of genome-wide association study for sex. X axis: expected −log10(*p*-values). Y axis: observed −log10(*p*-values).
(JPG)

S13 Fig Male linkage map for linkage analysis for genetic sex using F1 cross of family 1 derived from Nagaragawa River. (JPG)

S14 Fig Confirmation of sex-linked locus detected by GWAS using genetic linkage analysis using mapping family 1 derived from Nagaragawa River population. Logarithm of odds (LOD) curves of simple interval mapping were shown. The significance thresholds for LOD scores were determined by 10,000 permutation tests using R/qtl package. Horizontal bar represent 1.5-LOD support intervals.
(JPG)

S15 Fig Male linkage map for linkage analysis for genetic sex using F1 cross of family 2 derived from Nagaragawa River. (JPG)

S16 Fig Confirmation of sex-linked locus detected by GWAS using genetic linkage analysis of mapping family 2 derived from Nagaragawa River population. LOD curves of simple interval mapping were shown. The significance thresholds for LOD scores were determined by 10,000 permutation tests using R/qtl software. Horizontal bar represent 1.5-LOD support intervals.
(JPG)

S17 Fig Comparison of mapping coverage of whole-genome resequencing between males and females in sex-linked scaffolds. (A) Comparison of sequence depth between males and females in sex-associated scaffold A. (B) Comparison of sequence depth between males and females in sex-associated scaffold B. Y-axis indicates log-scaled sequence depth by whole-genome resequencing analysis; X-axis indicates physical position of the scaffold. Green boxes indicate predicted coding sequences. Orange boxes indicate Y-specific candidate coding sequences. Purple boxes indicate transposable elements detected by RepeatMasker. Blue line indicates putative male-specific regions.
(JPG)

S18 Fig PCR amplification of genomic regions containing candidate coding sequences. Results of agarose gel electrophoresis for 4 males and 4 females. M: size marker. White arrowheads: amplicon of Y allele. Black arrowheads: amplicon of X allele.
(JPG)

S19 Fig Transcript levels of candidate genes in adult testes and ovaries as determined by RT-PCR. M: size marker. RT+: Reverse transcription reaction with reverse transcriptase. RT-, RT reaction without reverse transcriptase.
(JPG)

S20 Fig Validation for sequence of sex-linked scaffold around strong candidate for the ayu sex-determining gene by BAC clone. (A) Comparison of mapping coverage of whole-genome resequencing between males and females in BAC clone containing *amhr2bY*. Y-axis indicates log-scaled sequence depth by whole-genome resequencing analysis; X-axis indicates physical position of the scaffold. Green box indicate predicted coding sequences. Red box indicate Y-linked *amhr2bY*. Purple boxes indicate transposable elements detected by RepeatMasker. (B) Linkage disequilibrium analysis using BAC clone carrying *amhr2bY*. Figure shows heat maps of pairwise linkage disequilibrium (r^2^) between SNPs (lower column) and scaffold-specific high-resolution association test (upper column) using SNPs genotyped by resequencing using ten males and nine females. X-axis indicates physical position of scaffolds. The numbers of SNPs in the figure indicate that the total number of SNPs using calculation for pairwise r^2^ value and scaffold-specific association test. Red line indicates scaffold-wide significance threshold (Bonferroni-corrected p-value = 0.05). Color of each SNP reflects pairwise r2 value: red indicates higher values, yellow indicates lower values. (C) Comparison for order of predicted genes around *amhr2bY* between scaffold C and BAC clone carrying *amhr2bY*.
(JPG)

S21 Fig PCR amplification of genomic region containing *amhr2bY* in scaffold C. Results of agarose gel electrophoresis using primer set amhr2bY-1F and amhr2bY-2R for 12 males and 12 females from Tama River population. M: size marker, NC: negative control.
(JPG)

S22 Fig Unrooted molecular phylogenetic tree using maximum likelihood method of ayu candidate sex-determining gene *Amhr2bY* and *Amhr2* of other teleost fishes. Bootstrap percentages based on 1000 replicates are indicated at nodes.
(JPG)

S23 Fig Multiple alignment of deduced amino acid sequences of ayu candidate sex-determining gene Amhr2bY, ayu’s autosomal paralog Amhr2a and Amhr2s of other teleost fishes and human. Blue box indicates activin type I and II receptor domain (amino acids 53–104 in Amhr2bY and 54–105 in Amhr2a). Green box indicates transmembrane domain (amino acids 135–151 in Amhr2bY and 136–152 in Amhr2a). Red boxes indicate catalytic domain of the serine/threonine kinases, bone morphogenetic protein, and anti-Müllerian hormone type II receptors (amino acids 208–492 in Amhr2bY and 209–507 in Amhr2a). Arrowheads indicate amino acid residues in evolutionarily conserved ATP binding site in catalytic domain of serine/threonine kinases in BMPR2 and AMHR2 family. Red arrowhead indicates ayu Amhr2bY-specific amino acid change in ATP binding site. Blue arrowhead indicates ayu-specific amino acid change in ATP binding site. Blue arrows indicate Amhr2bY-specific amino acid changes. Green arrows and green line indicate amino acid changes associated with human persistent Müllerian duct syndrome [70]. Orange arrow indicates amino acid residues critical for amhr2 function, as identified in medaka mutant [69]. It was conserved between Amhr2Yb and Amhr2a of ayu and that of other teleosts. Black arrow indicates amino acid residue determining genetic sex of fugu [13]. This amino acid did not alter between Amhr2bY and Amhr2a. Blue line indicates Amhr2a-specific 15-amino acid insertion.
(JPG)

S24 Fig Predicted protein structures of Amhr2bY and Amhr2a. (A) Homology model of Amhr2bY (blue) with human BMPR2 as template (gray) generated using SWISS-MODEL. Red amino acid residues are Q267. (B) Homology model of Amhr2a (blue) with human BMPR2 as template (gray) generated using SWISS-MODEL. Red amino acid residues indicate 15-amino acid insertion. (C) Validation of 15-amino acid insertion in amhr2a by RT-PCR.
(JPG)

S25 Fig Comparison of 30-kb upstream and downstream genomic sequences between *amhr2bY* and autosomal *amhr2a*. Dot plots of pairwise alignment are shown. Only exon regions of *amhr2* showed similarities.
(JPG)

S26 Fig Comparison of predicted transcription factor binding sites in putative promoter regions of *amhr2bY* and autosomal *amhr2a*. (A) Comparison of predicted transcription factor binding sites for sex differentiation-related genes in intergenic region between *amhr2s* and adjacent gene in ayu, northern pike and medaka. Multiple binding sites were detected for some sex differentiation-related transcription factors, as follows: 32 sox9 binding sites, 18 sox3 binding sites, 20 nr5a1 (sf1) binding sites, five dmrt1 binding sites, six foxl2 binding sites and two esr2 (estrogen receptor β) binding sites. In the putative promoter region of autosomal amhr2a (1,327 bp), 2,361 binding sites for 418 transcription factors were detected, including three sox9 binding sites, two sox3 binding sites, two nr5a1 binding sites and one foxl2 binding site. (B) Venn diagram showing number of transcription factors with predicted binding sites in putative promoter regions of *amhr2bY*, *amhr2a*, northern pike *amhr2* and medaka *amhr2*. In total, 398 transcription factors had predicted binding sites in the promotor regions of both amhr2bY and amhr2a. Comparisons of the regulatory regions of amhr2s among ayu, northern pike and medaka, revealed 26 transcription factors that regulated only amhr2bY.
(JPG)

S27 Fig Histological analysis of ayu gonadal development. Tissue sections were stained with hematoxylin–eosin. Future gonadal region of ayu larvae at 39 days post fertilization in XY (A) and XX (B). Ayu gonads at 2 months post fertilization (mpf) XY (C), 2 mpf XX (D), 3.5 mpf XY (E), 3.5 mpf XX (F), 7 mpf XY (G), and 7 mpf XX (H). Arrowheads in C and D indicate germ cells. Asterisk in G indicates sperm. Dotted lines indicate gonad outline. Scale bar: 10 μm in A–E and 100 μm in F–H.
(JPG)

S28 Fig Pairwise alignment of full-length cDNA sequences of ayu *amhr2bY* and *amhr2a* obtained by 5′ and 3′ RACE using clustalW. Blue box indicates start codon, red box indicates stop codon. Blue line indicates RNA probe position for 3′ UTR of *amhr2bY*. Green line indicates RNA probe position for 3′ UTR of autosomal *amhr2a*. Asterisks indicate identical nucleotides. Red characters indicate the target site for guide RNA of CRISPR/Cas9. Underline indicates PAM sequence.
(JPG)

S29 Fig Expression of ayu *amhr2bY* and autosomal *amhr2a* in morphologically undifferentiated gonads. (A–D) *In situ* hybridization using antisense (A and B) and sense (C and D) probes of *amhr2bY*-containing coding region at 2 mpf XY (A and C) and 2 mpf XX (B and D). (E and F) *In situ* hybridization using probe for 3′ UTR of *amhr2bY* at 2 mpf XY (E) and 2 mpf XX (F). (G and H) *In situ* hybridization for 3′ UTR of *amhr2a* at 2 mpf XY (G) and 2 mpf XX (H). *amhr2bY* mRNA was specifically expressed in somatic cells of genetic males with undifferentiated gonads. Autosomal *amhr2a* mRNA was detected in both XY and XX undifferentiated gonads. Arrowheads indicate positive signals. Scale bar: 10 μm. (I) Validation of specificity of RNA probes by dot blot hybridization. RNA probe for *amhr2bY*-containing coding region detected both *amhr2bY* and autosomal *amhr2a* RNA, whereas probe derived from 3′ UTR of *amhr2bY* detected only *amhr2bY* RNA and not *amhr2a* RNA. Similarly, probe for 3′ UTR of *amhr2a* RNA only detected *amhr2a*.
(JPG)

S30 Fig Development of PCR-based genotyping method for genetic sex identification in ayu. (A) Agarose gel electrophoresis results using primer set Ayu-sex-1F and Ayu-sex-2R for 12 males and 12 females from Tama River population. (B) Agarose gel electrophoresis results using primer set Ayu-sex-3F and Ayu-sex-4R for 12 males and 12 females from Tama River population. Genetic males have two amplified bands derived from *amhr2bY* (arrowhead) and autosomal *amhr2a*; genetic females have one amplified band derived from autosomal *amhr2b* (arrow). M: size marker.
(JPG)

S1 Table Whole-genome sequencing results. (XLSX)

S2 Table The summary of the genetic linkage map constructed in ayu. (XLSX)

S3 Table Marker sequences of linkage maps. (XLSX)

S4 Table Summary for *de novo* gene prediction by Augustus. (XLSX)

S5 Table Whole-genome resequencing results. (XLSX)

S6 Table Estimation of linkage disequilibrium in ayu wild population by SNPs using association mapping for sex-determining locus. (XLSX)

S7 Table Summary of paternal linkage map for genetic sex of Nagaragawa River family 1. (XLSX)

S8 Table Summary of paternal linkage map for genetic sex of Nagaragawa River family 2. (XLSX)

S9 Table The candidates for sex determining gene located in putative male specific regions. (XLSX)

S10 Table Prediction of transcription factor binding sites in putative promoter regions of *amhr2bY*, *amhr2a*, northern pike *amhr2* and medaka *amhr2*. (XLSX)

S11 Table PCR-based genotyping for genetic sex identification by *amhr2bY* and *amhr2a* using various population of ayu. (XLSX)

S12 Table PCR primers used for cloning and expression analyses. (XLSX)

S13 Table PCR primers and conditions used to detect candidates for ayu sex-determining gene. (XLSX)

S14 Table GenBank accession numbers for sequences used in phylogenetic analysis. (XLSX)

S15 Table Marker sequences of linkage map for genetic sex of family 1 derived from Nagaragawa River population. (XLSX)

S16 Table Marker sequences of linkage map for genetic sex of family 2 derived from Nagaragawa River population. (XLSX)

S1 Text Supplemental Materials and Methods, References. (DOCX)
