# Supplementary figures and images for: A Y-linked anti-Müllerian hormone type-II receptor is the sex-determining gene in ayu, Plecoglossus altivelis
Source: PLoS Genet. 2021 Aug 26;17(8):e1009705. doi: 10.1371/journal.pgen.1009705 (PMC8389408; doi:10.1371/journal.pgen.1009705)

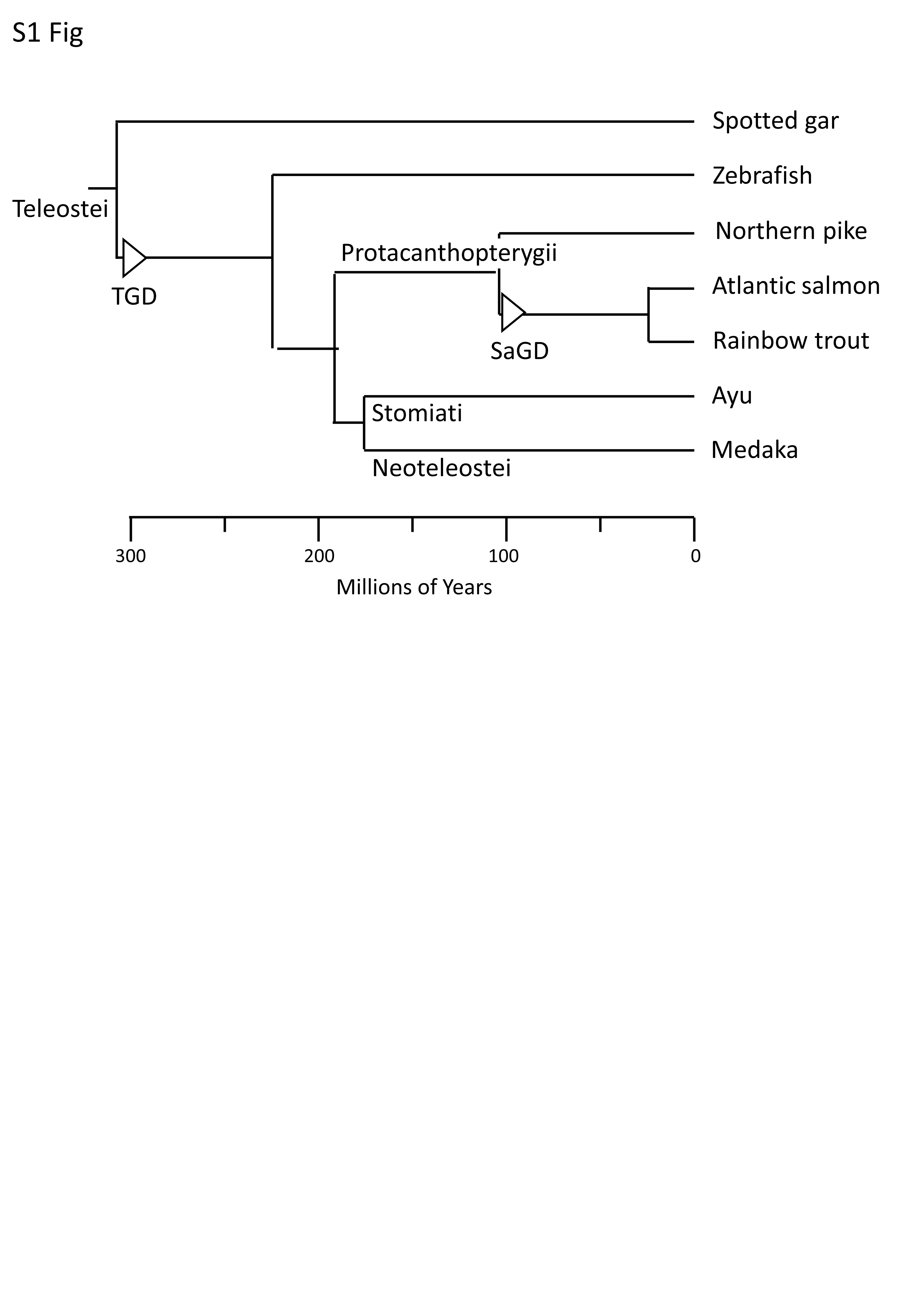

Supplement: S1 Fig — TGD: Teleost-specific whole-genome duplication event. SaDG: Salmonid-specific whole-genome duplication even. Phylogenetic tree was illustrated to Hughes et al [22]. (JPG) [file pgen.1009705.s001.JPG]

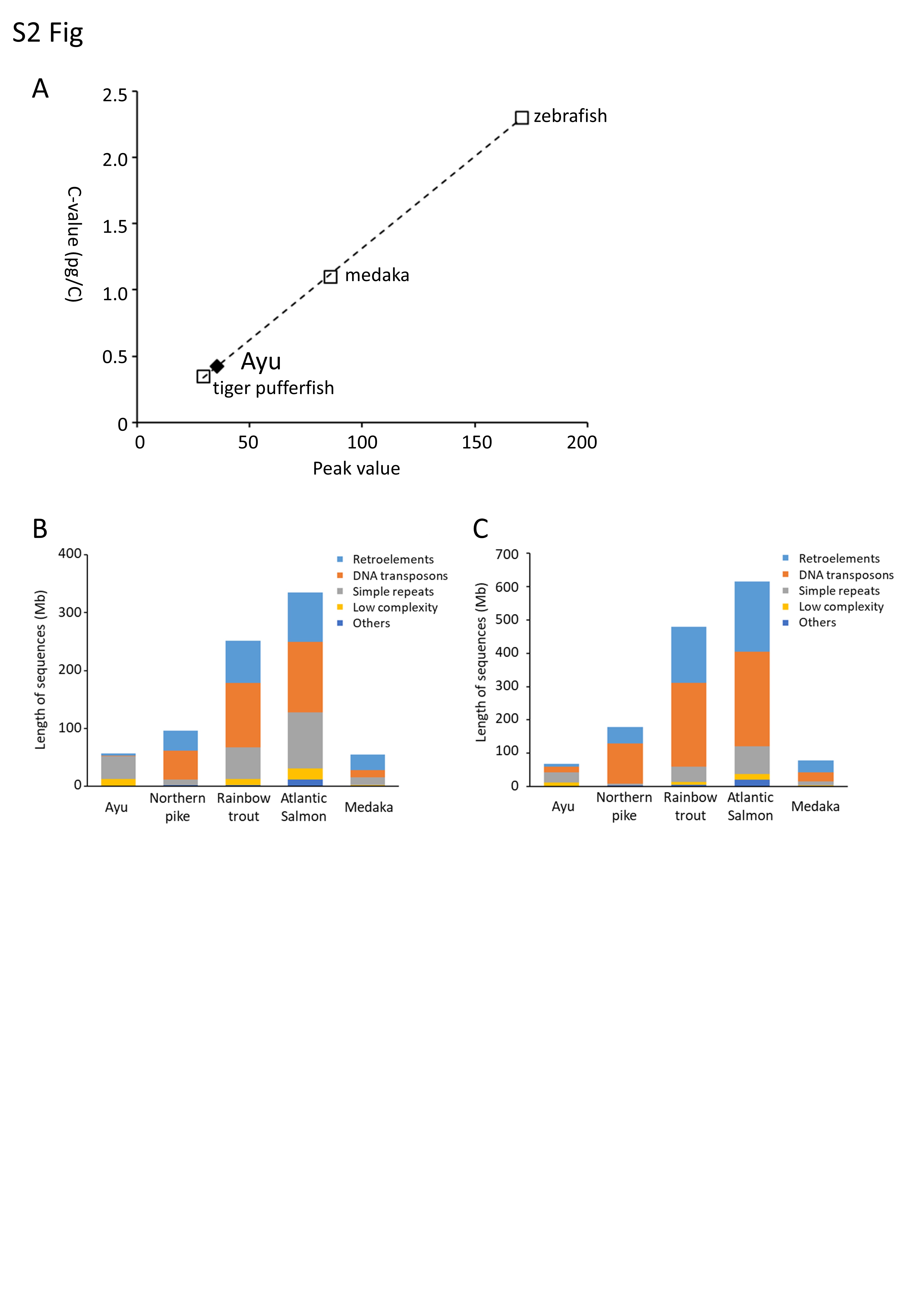

Supplement: S2 Fig — (A) Estimation of ayu genome size by nuclear DNA staining. Calibration curve was constructed using nuclear DNA of tiger pufferfish, medaka and zebrafish. X-axis, fluorescence peaks; Y-axis, C-value (pg). (B) Comparison of predicted repetitive elements in ayu and other teleosts by RepeatMasker using fugu repeat databases. Repetitive elements accounted for 56.78 Mb (12.61% of the ayu genome assembly): 39.46 Mb (8.76%) were simple repeats, 12.05 Mb (2.67%) were low complexity repeats, 4.20 Mb (0.93%) were retroelements, and 0.96 Mb (0.21%) were DNA transposons. (C) Comparison of predicted repetitive elements in ayu and other teleosts by RepeatMasker using zebrafish repeat databases. Repetitive elements predicted for 65.15 Mb (14.47%): 29.60 Mb (6.57%) were simple repeats, 11.14 Mb (2.47%) were low complexity repeats, 8.65 Mb (1.92%) were retroelements, and 17.84 Mb (3.96%) were DNA transposons. Y-axis indicates length of repetitive elements. (JPG) [file pgen.1009705.s002.JPG]

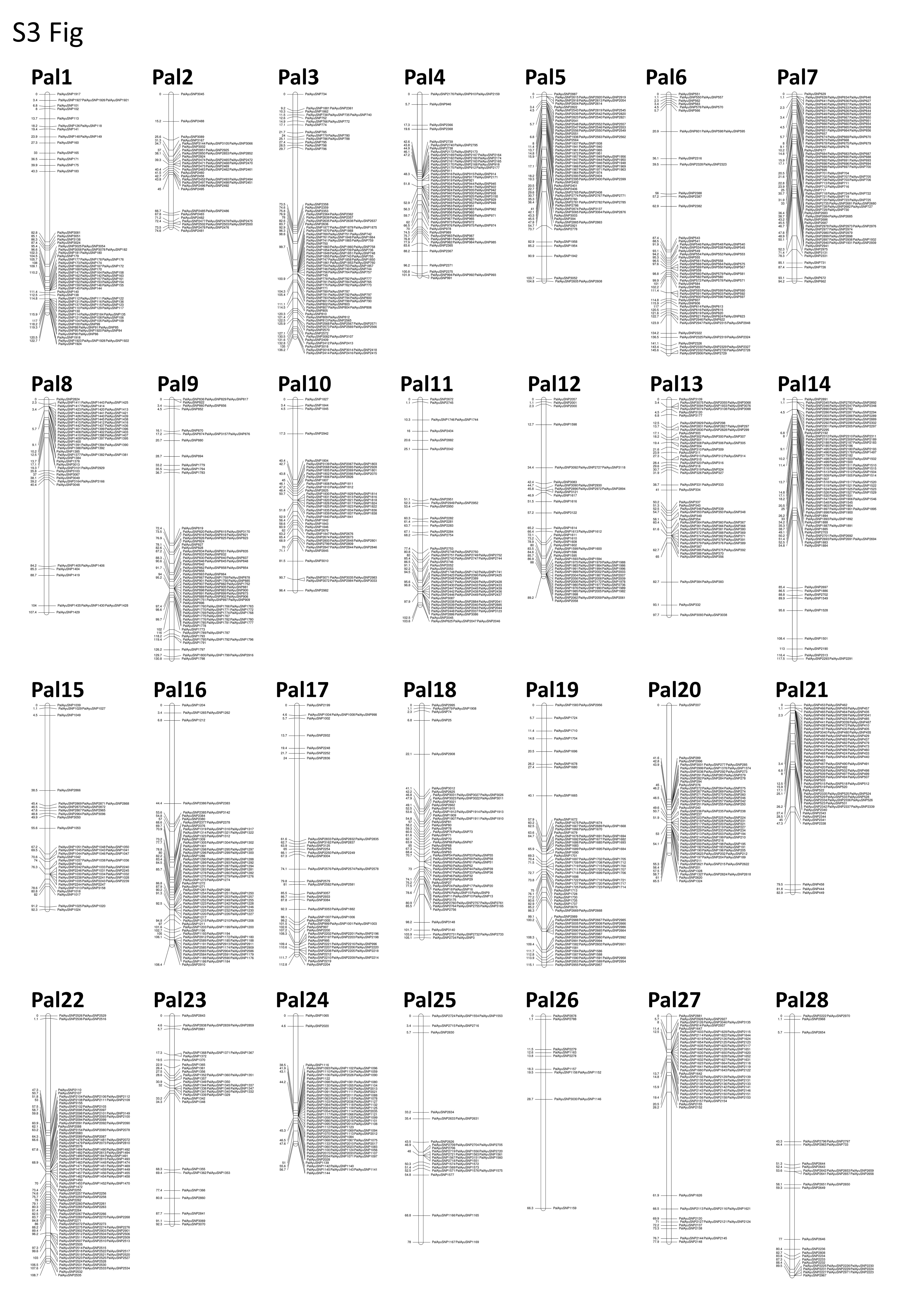

Supplement: S3 Fig — (JPG) [file pgen.1009705.s003.JPG]

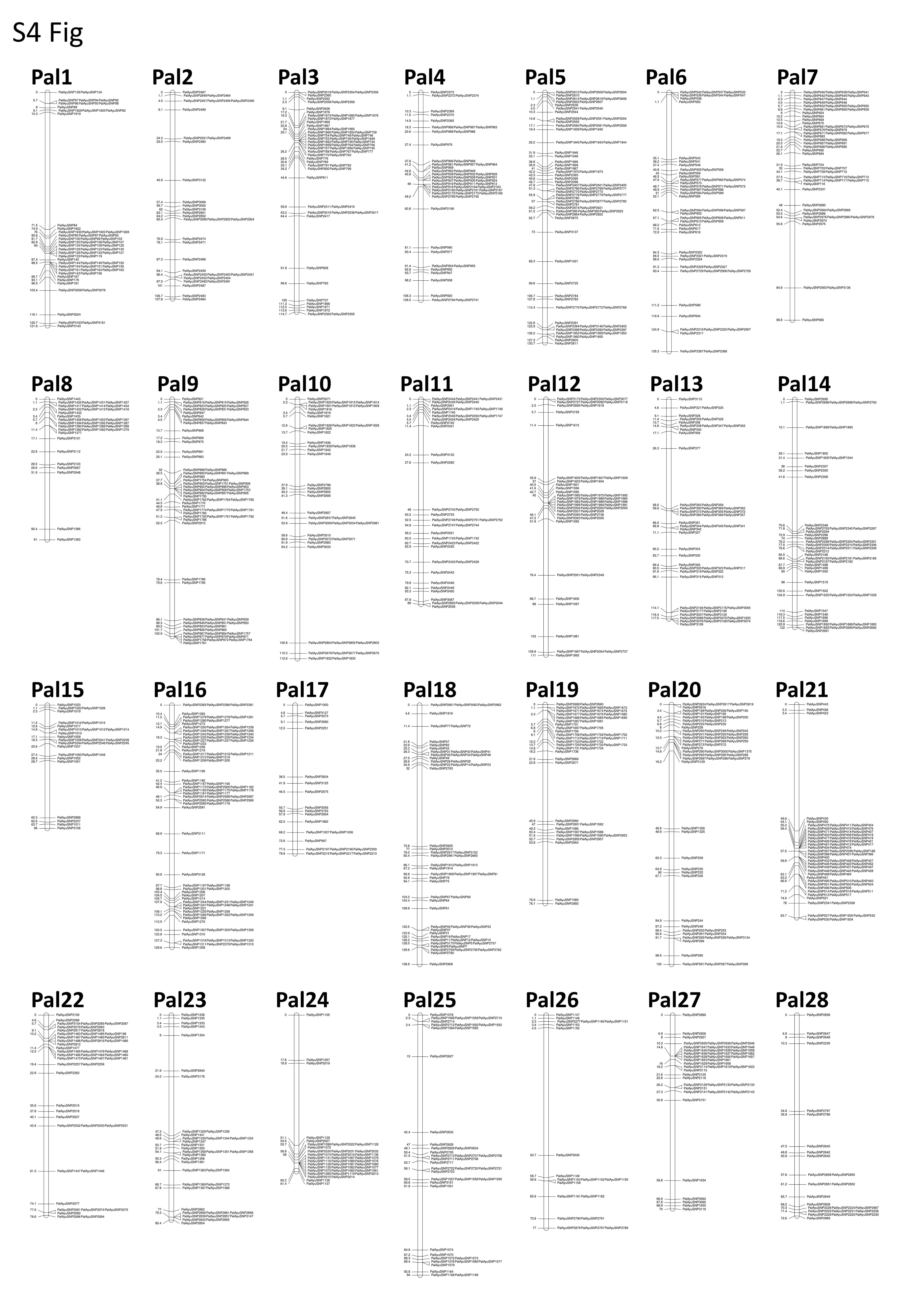

Supplement: S4 Fig — (JPG) [file pgen.1009705.s004.JPG]

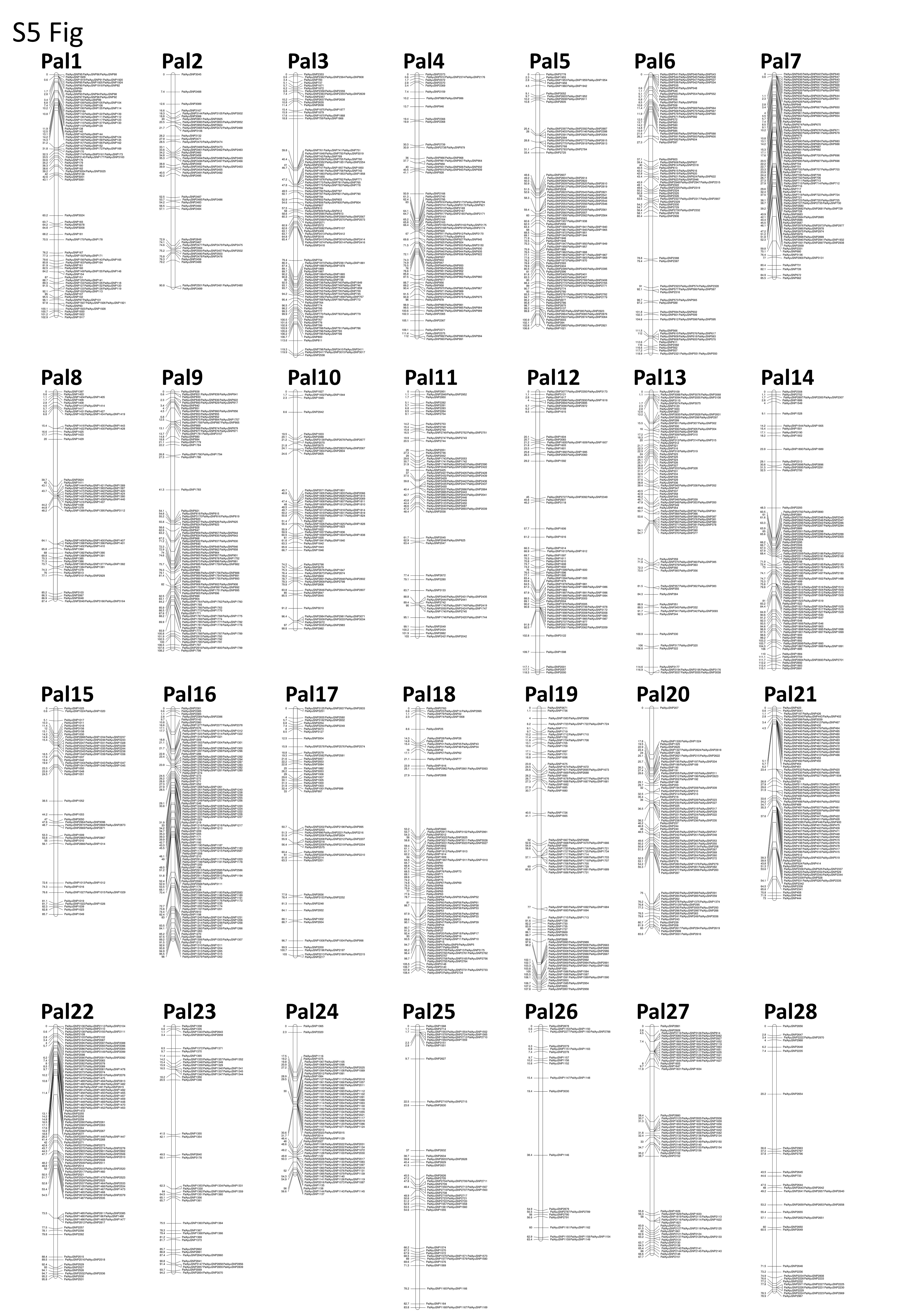

Supplement: S5 Fig — (JPG) [file pgen.1009705.s005.JPG]

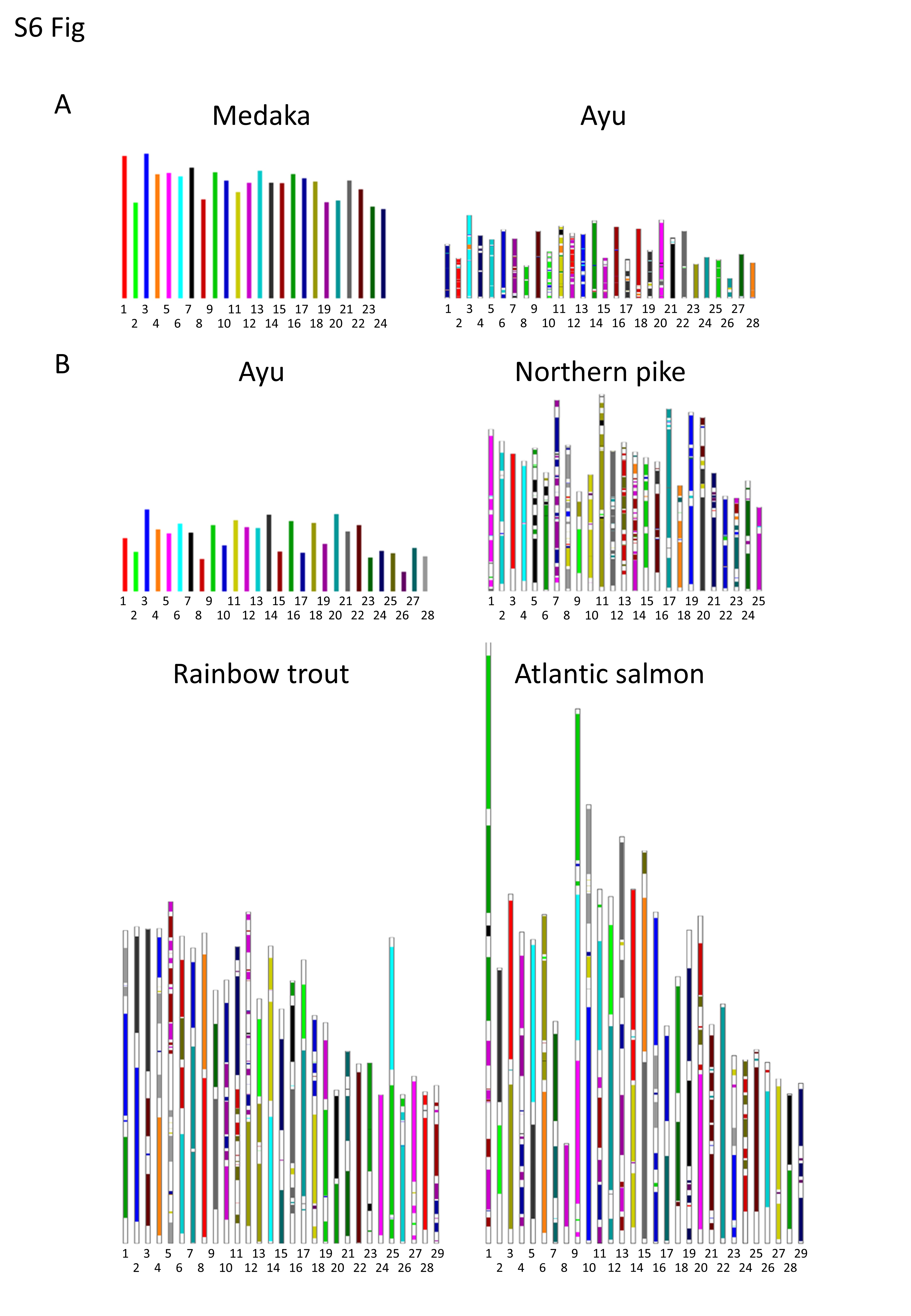

Supplement: S6 Fig — (A) Bar plot of pairwise syntenic blocks between medaka and ayu. (B) Bar plot of pairwise syntenic blocks between ayu, northern pike, rainbow trout and Atlantic salmon. Colors mark chromosomal location of conserved syntenic blocks. White indicates regions for which no information for syntenic blocks is available. (JPG) [file pgen.1009705.s006.JPG]

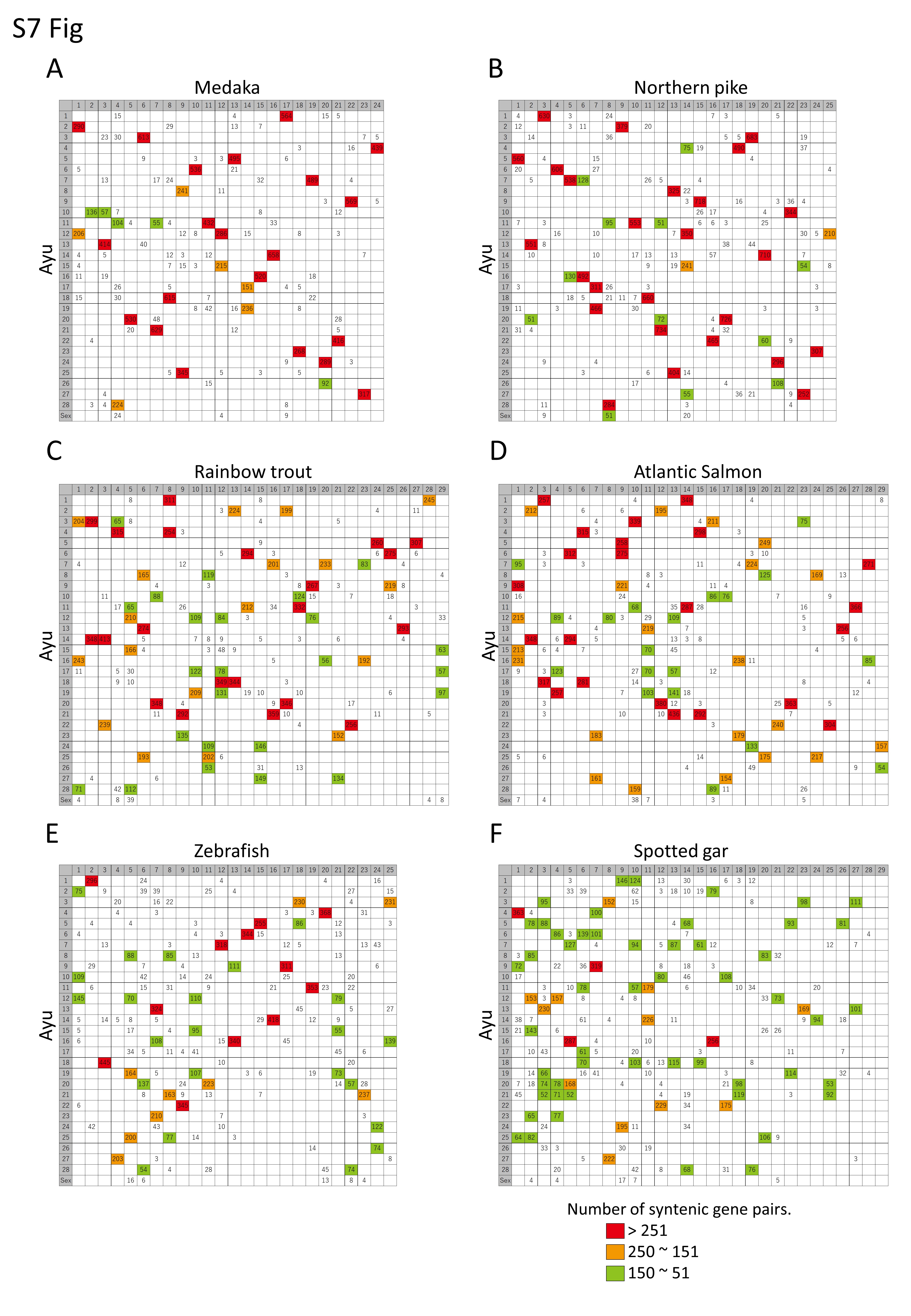

Supplement: S7 Fig — Oxford plot between ayu genome and (A) medaka, (B) northern pike, (C) rainbow trout, (D), Atlantic salmon, (E) zebrafish, and spotted gar (F). Numbers refer to pairs of orthologous genes clustered by reciprocal best hits algorithm. “Sex” in bottom row of each panel indicates sex-linked scaffolds detected by genome-wide association study, not only putative Y-specific regions. (JPG) [file pgen.1009705.s007.JPG]

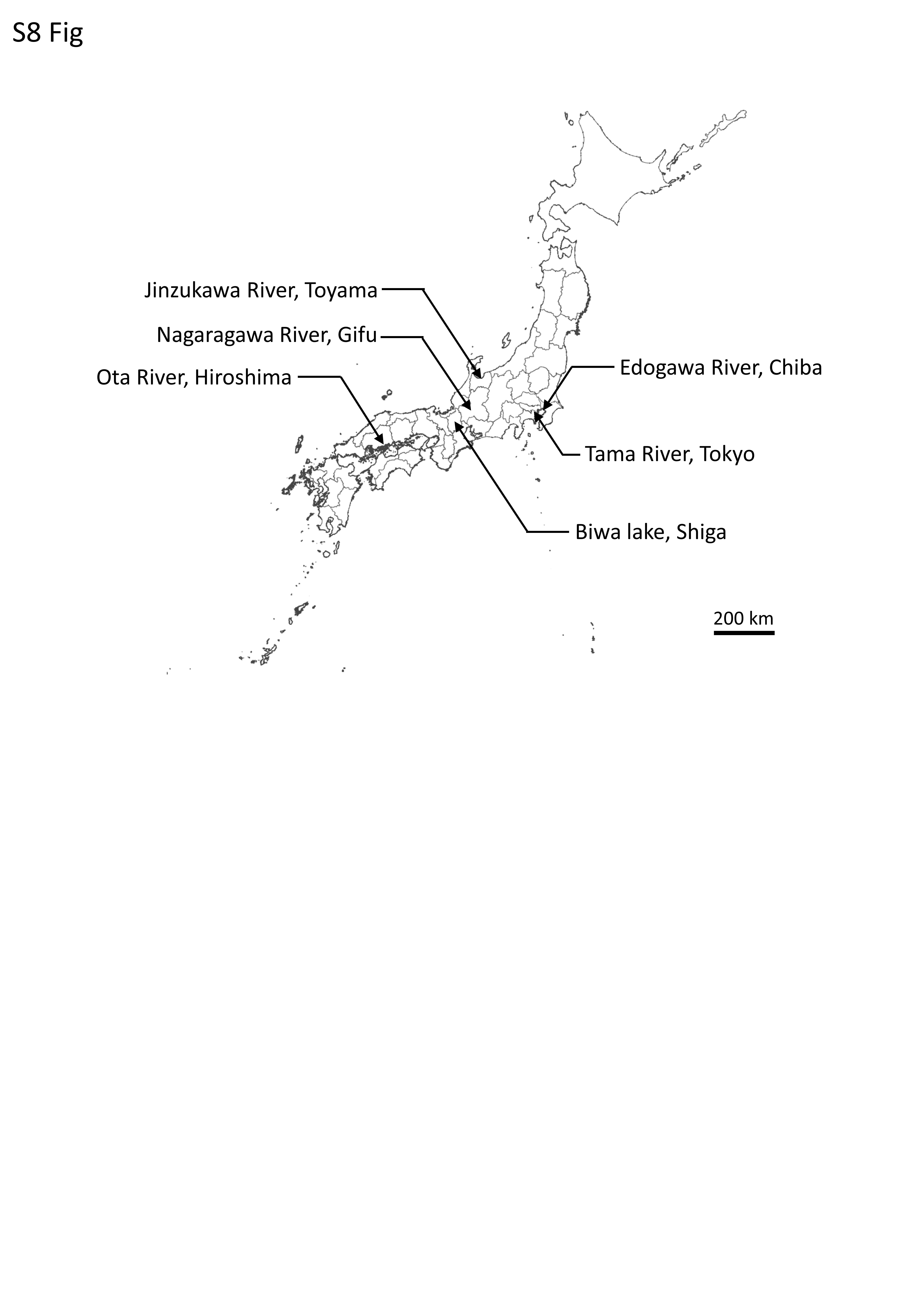

Supplement: S8 Fig — Map data was based on the Digital Map published by Geospatial Information Authority of Japan (https://maps.gsi.go.jp/). (JPG) [file pgen.1009705.s008.JPG]

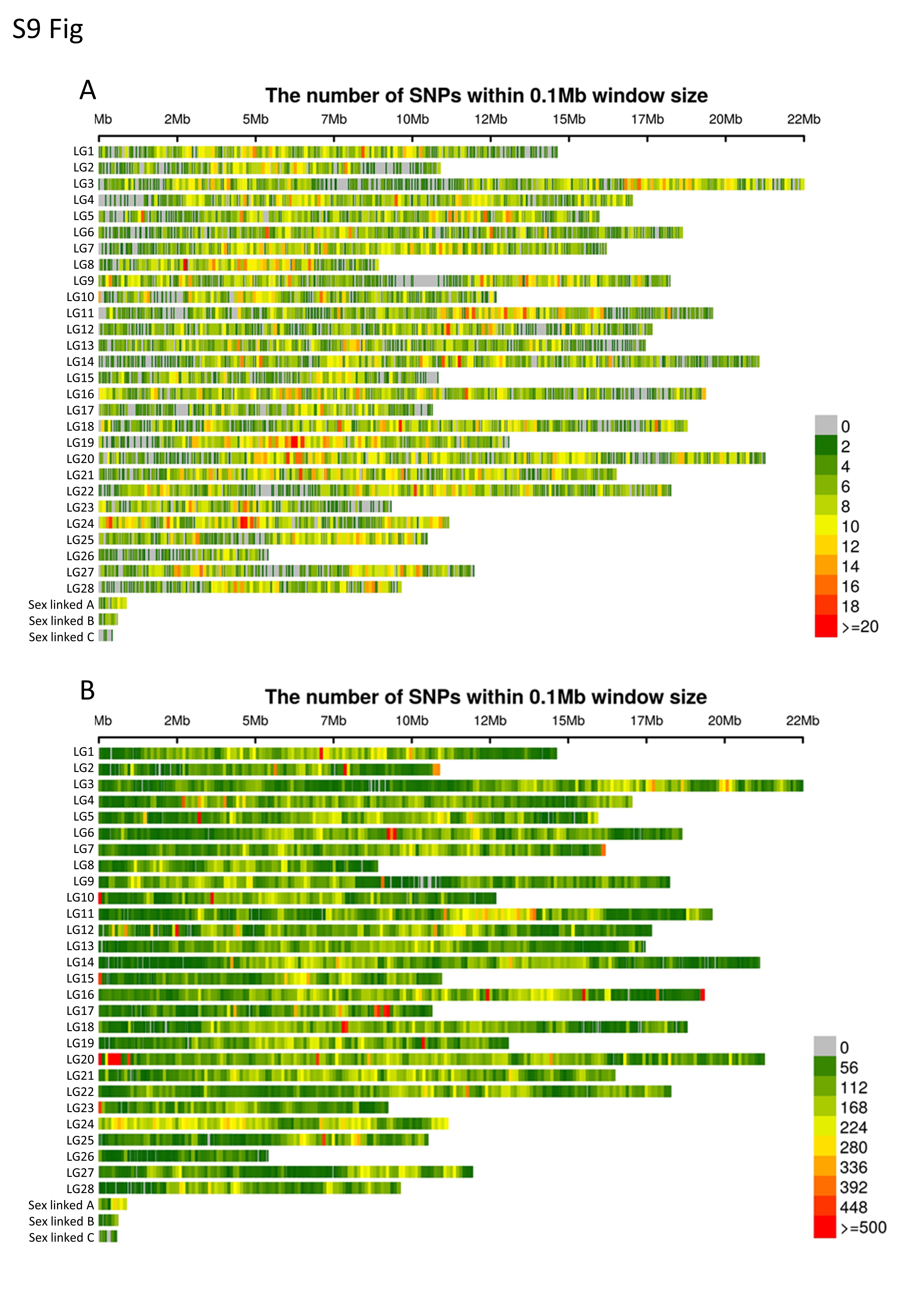

Supplement: S9 Fig — (A) Density of SNPs using genome-wide association scan in a 100-kb window size by genotyping-by-sequencing (GBS) and whole-genome resequencing. (B) Density of SNPs detected by whole-genome resequencing in a 100-kb window size. (JPG) [file pgen.1009705.s009.JPG]

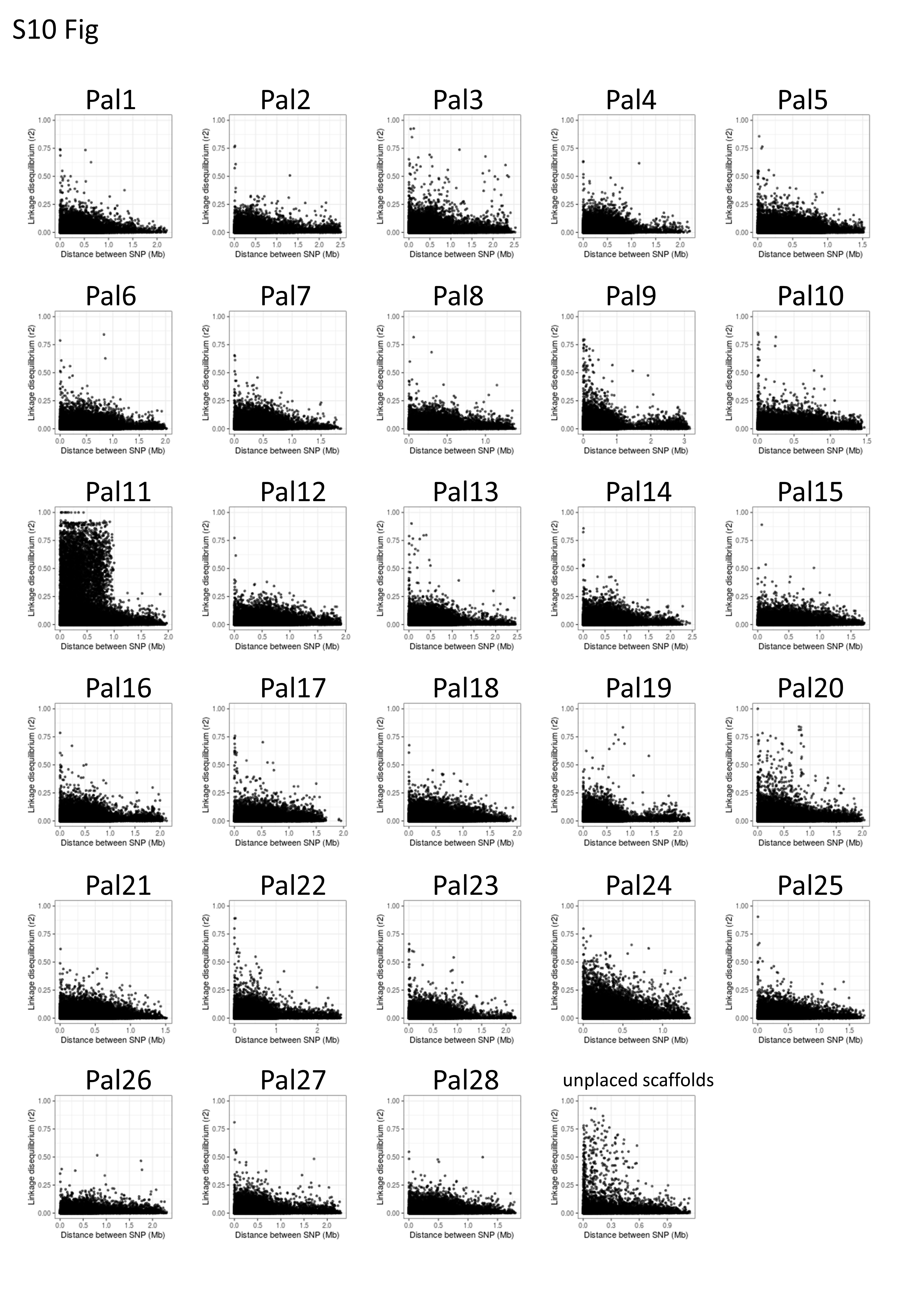

Supplement: S10 Fig — X axis: physical distance between SNPs. Y axis: pairwise linkage disequilibrium (r2). (JPG) [file pgen.1009705.s010.JPG]

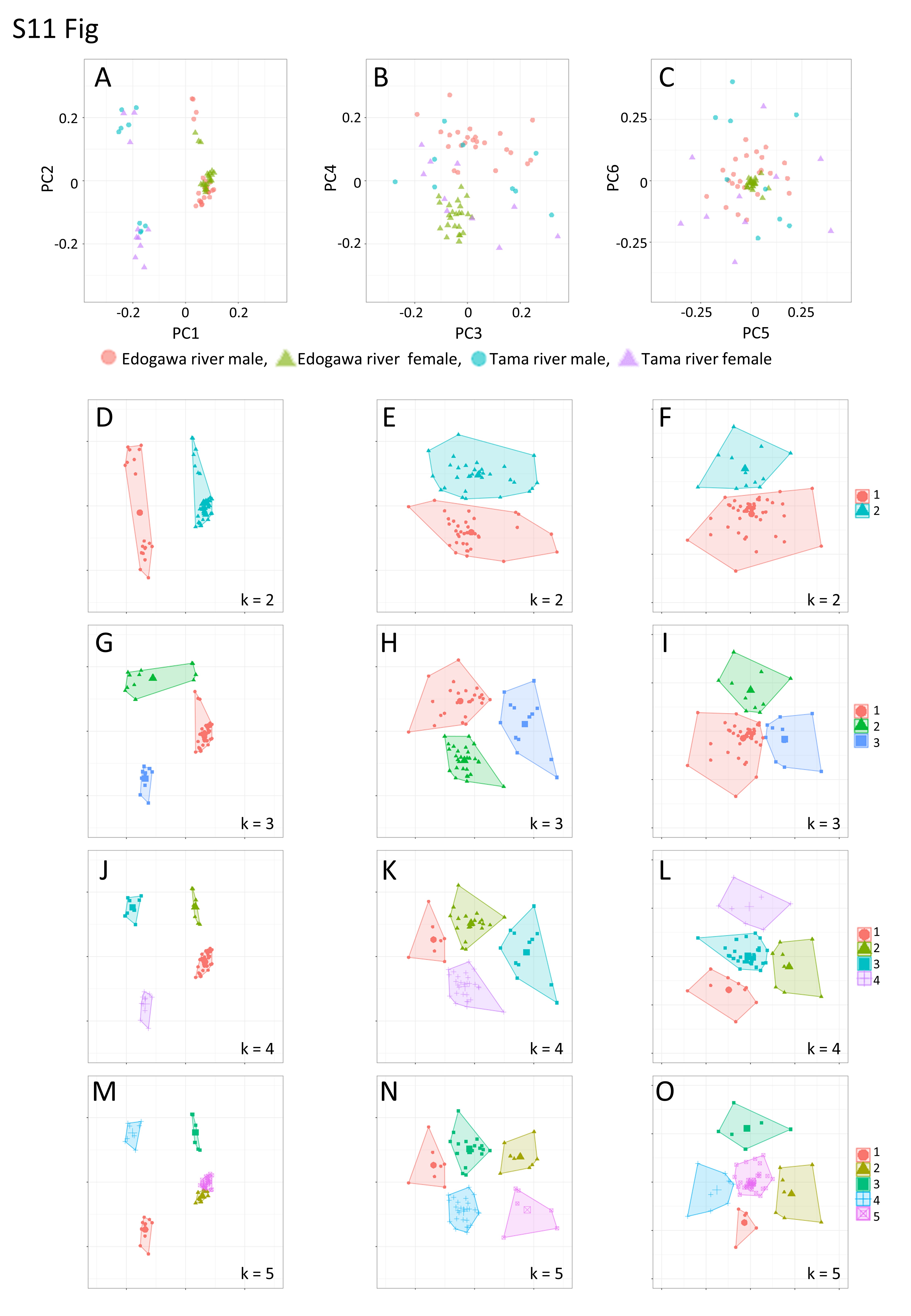

Supplement: S11 Fig — (A) PCA scores plot pc1 versus pc2. (B) PCA scores plot pc3 versus pc4. (C) PCA scores plot pc5 versus pc6. (D–O) Subgroups with similar SNP distributions clustered by k-means clustering algorithm on pc1 versus pc2 (D, G, J and M), pc3 versus pc4 (E, H, K and N) and pc5 versus pc6 (F, I, L and O). Cluster number with k set to 2 (D–F), 3 (G–I), 4 (J–L) and 5 (M–O). Large markers indicate center of cluster. Edogawa River population based on GBS and Tama River population based on resequencing are separated on pc1. Edogawa River males and females are well separated on pc4, but Tama River sexes are not as well separated. (JPG) [file pgen.1009705.s011.JPG]

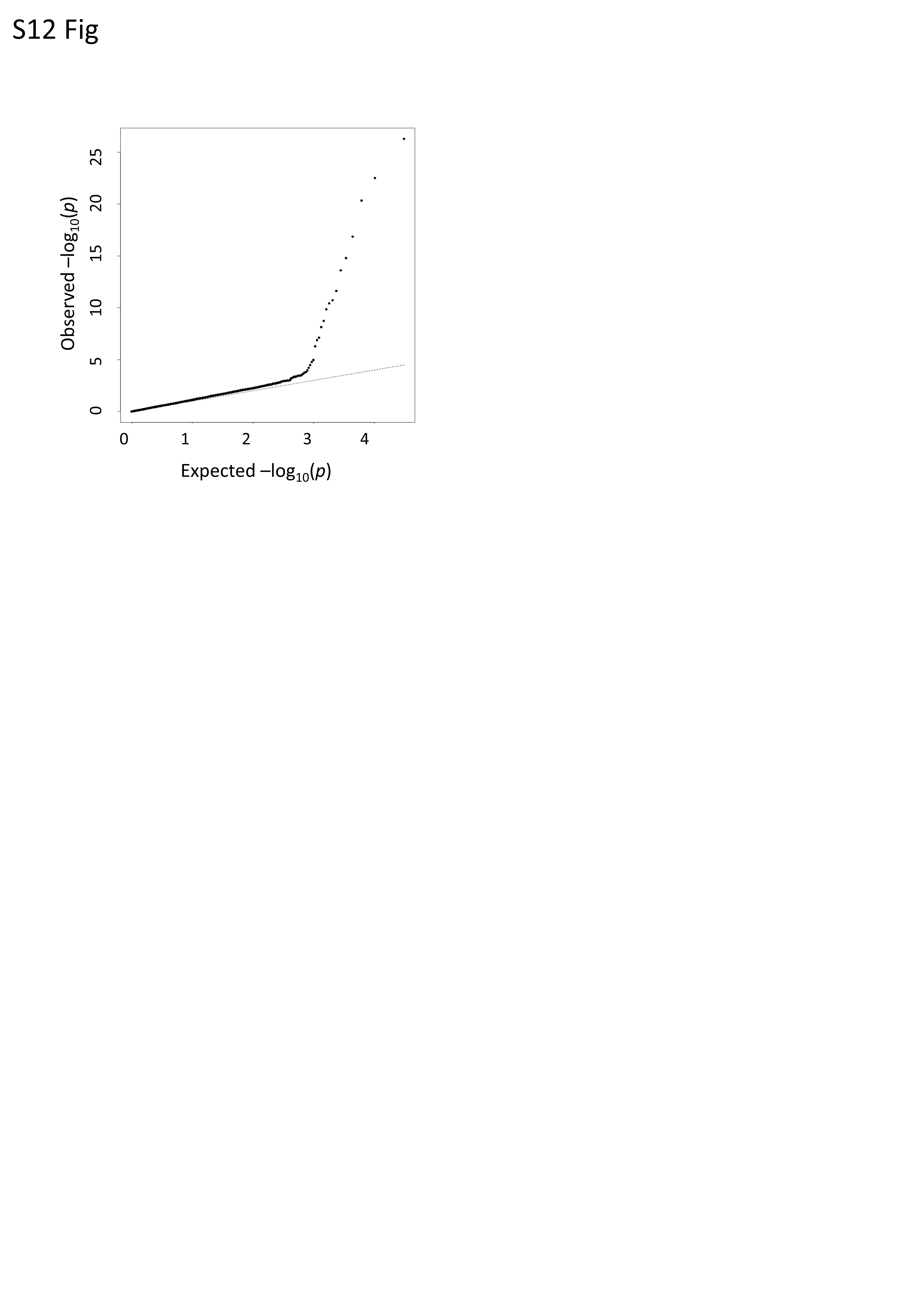

Supplement: S12 Fig — X axis: expected −log10(p-values). Y axis: observed −log10(p-values). (JPG) [file pgen.1009705.s012.JPG]

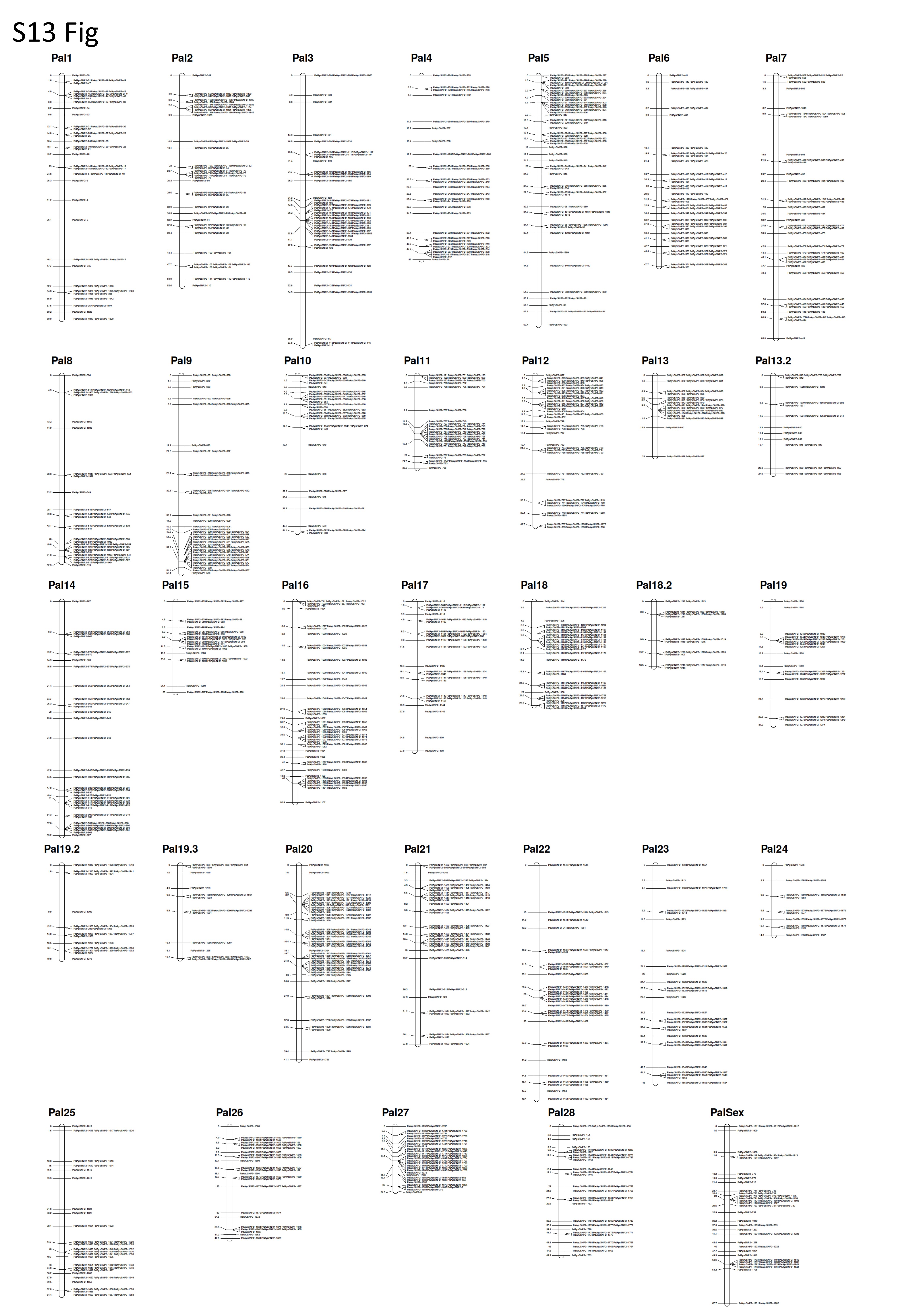

Supplement: S13 Fig — (JPG) [file pgen.1009705.s013.JPG]

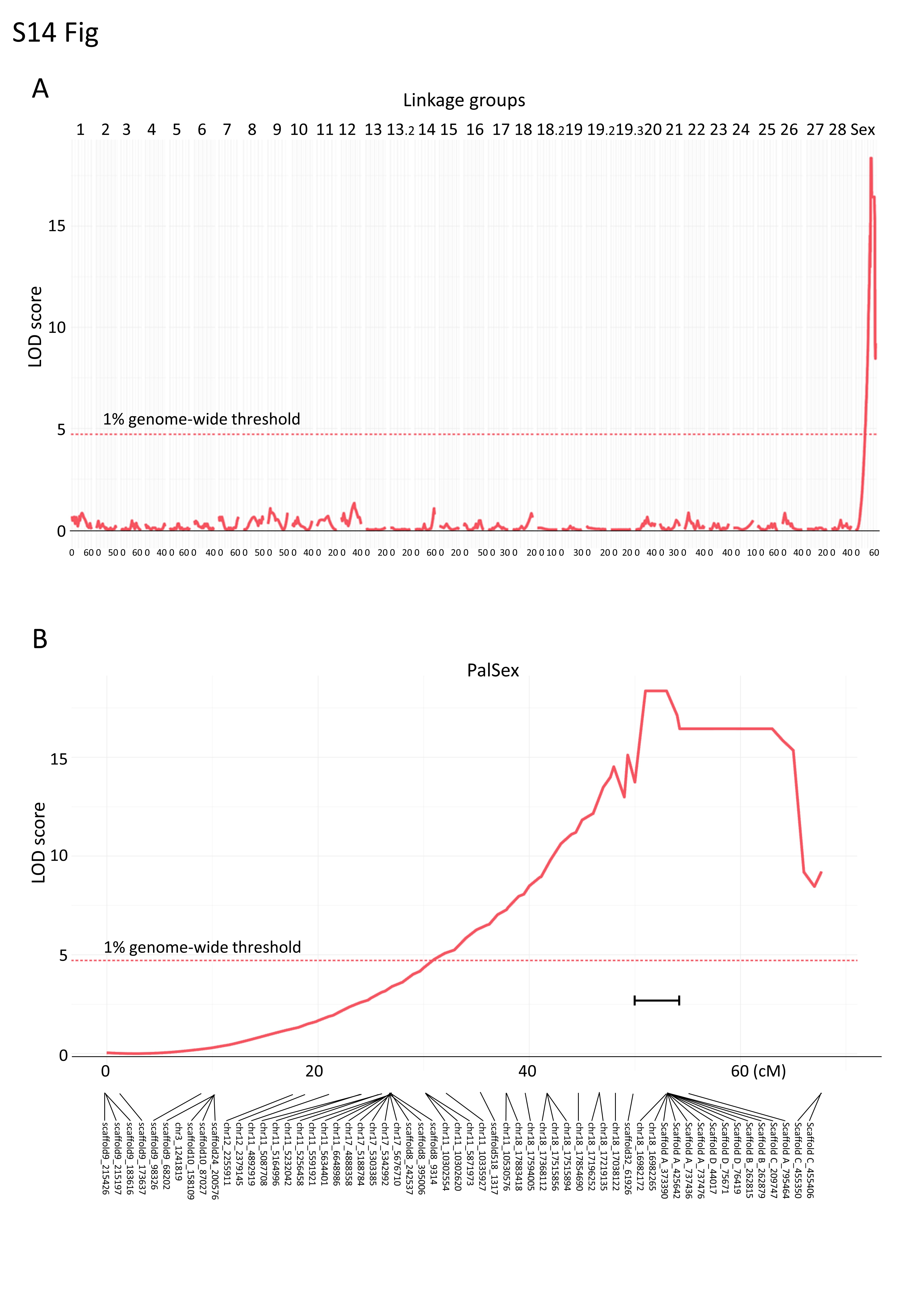

Supplement: S14 Fig — Logarithm of odds (LOD) curves of simple interval mapping were shown. The significance thresholds for LOD scores were determined by 10,000 permutation tests using R/qtl package. Horizontal bar represent 1.5-LOD support intervals. (JPG) [file pgen.1009705.s014.JPG]

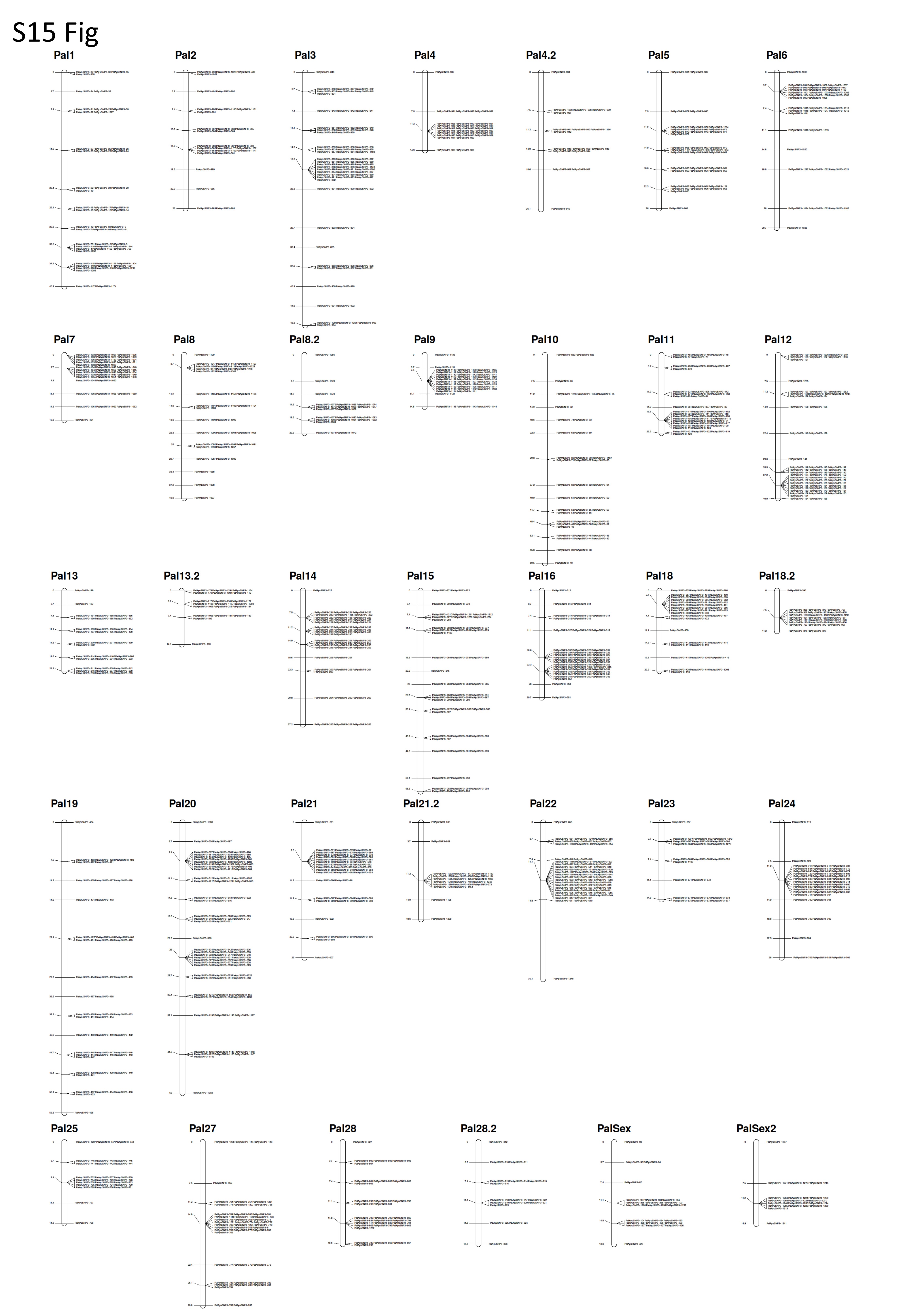

Supplement: S15 Fig — (JPG) [file pgen.1009705.s015.JPG]

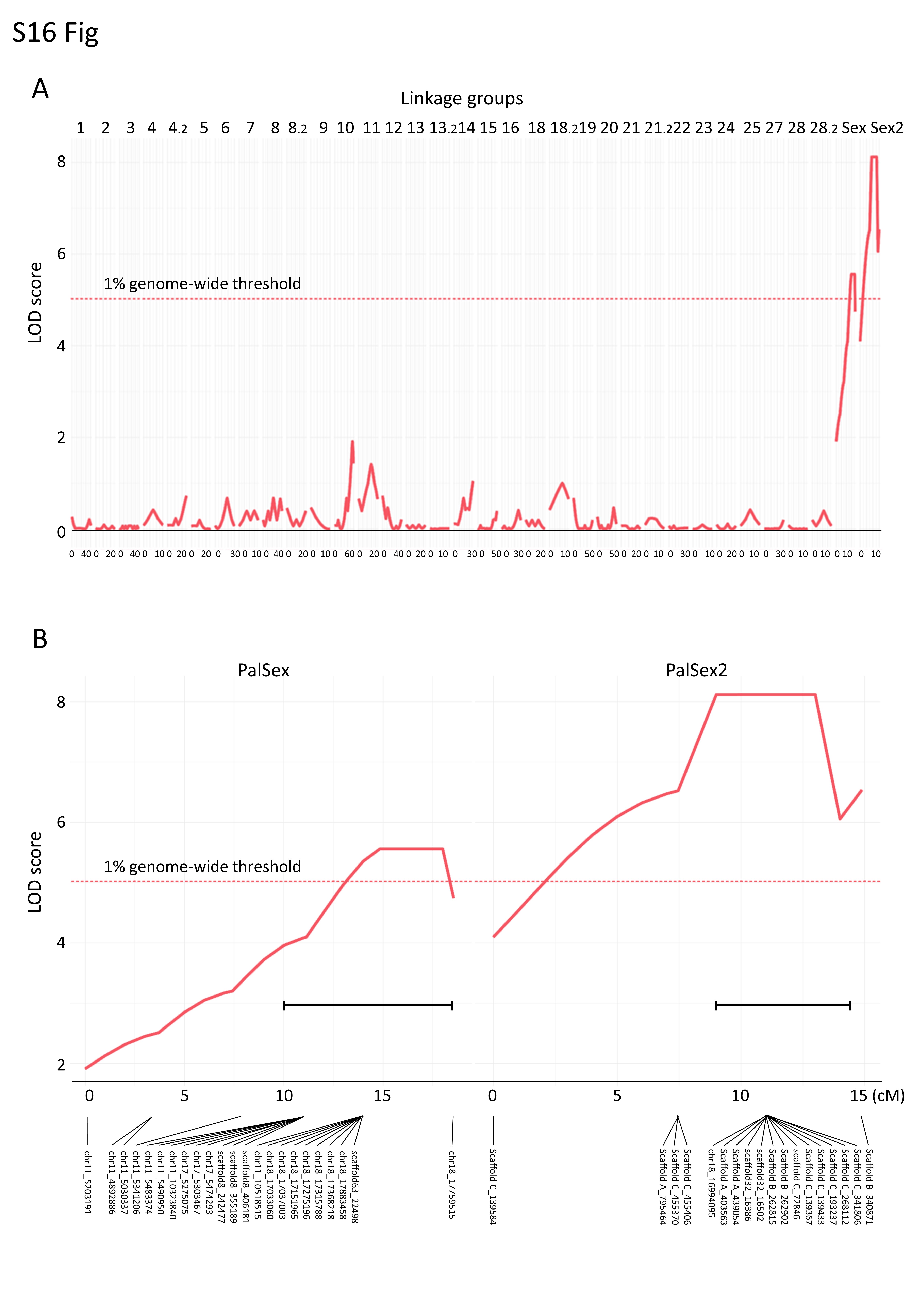

Supplement: S16 Fig — LOD curves of simple interval mapping were shown. The significance thresholds for LOD scores were determined by 10,000 permutation tests using R/qtl software. Horizontal bar represent 1.5-LOD support intervals. (JPG) [file pgen.1009705.s016.JPG]

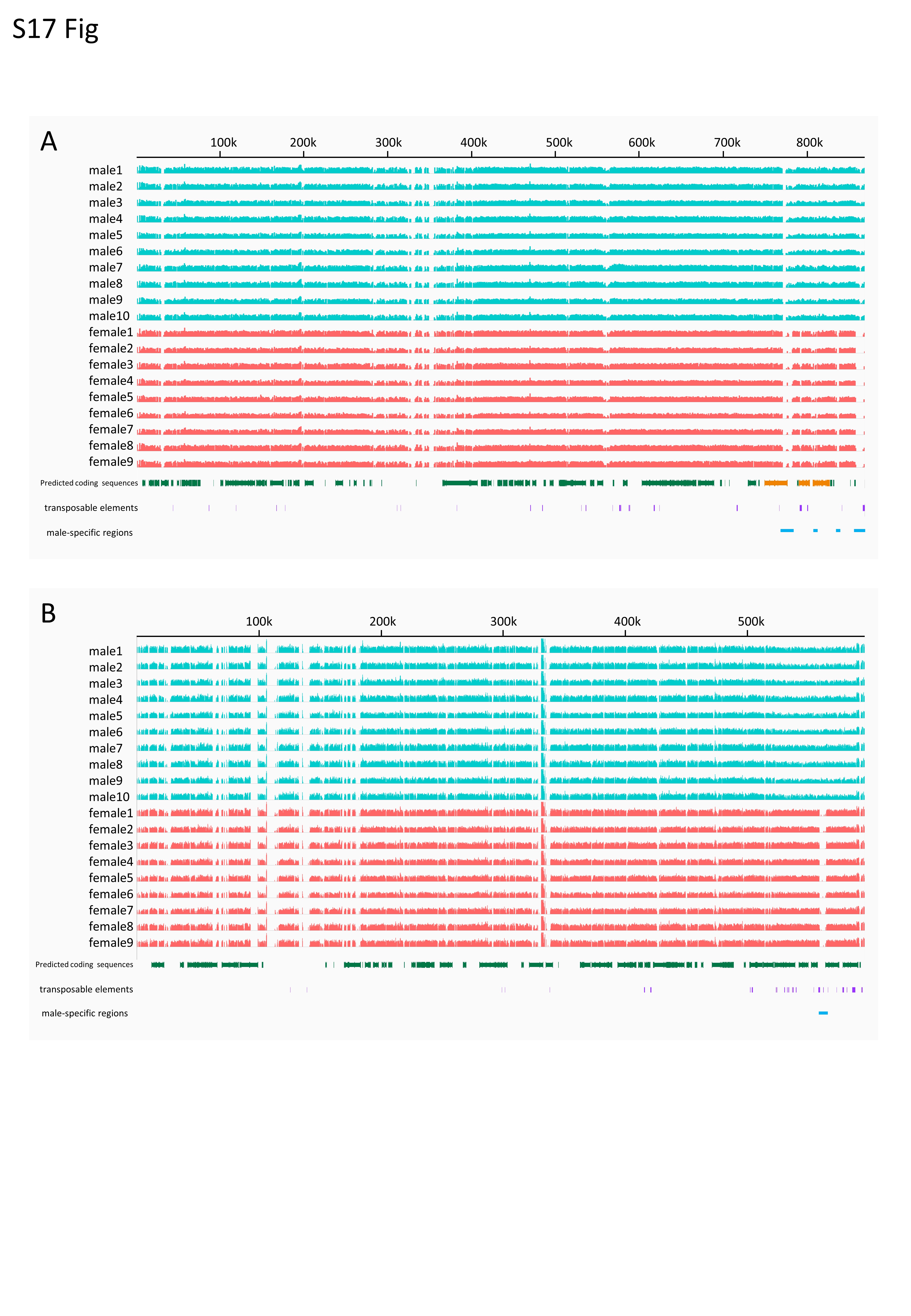

Supplement: S17 Fig — (A) Comparison of sequence depth between males and females in sex-associated scaffold A. (B) Comparison of sequence depth between males and females in sex-associated scaffold B. Y-axis indicates log-scaled sequence depth by whole-genome resequencing analysis; X-axis indicates physical position of the scaffold. Green boxes indicate predicted coding sequences. Orange boxes indicate Y-specific candidate coding sequences. Purple boxes indicate transposable elements detected by RepeatMasker. Blue line indicates putative male-specific regions. (JPG) [file pgen.1009705.s017.JPG]

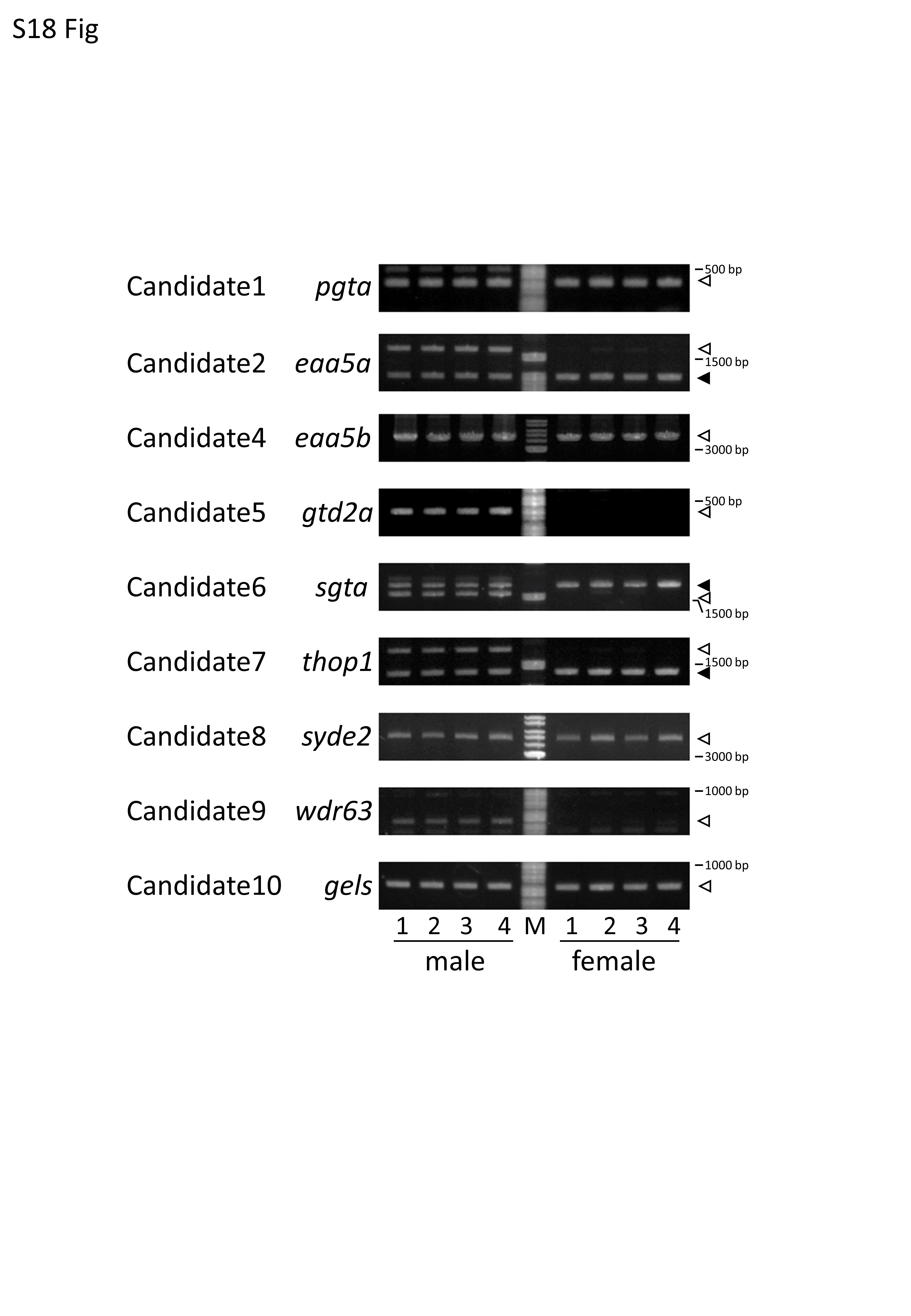

Supplement: S18 Fig — Results of agarose gel electrophoresis for 4 males and 4 females. M: size marker. White arrowheads: amplicon of Y allele. Black arrowheads: amplicon of X allele. (JPG) [file pgen.1009705.s018.JPG]

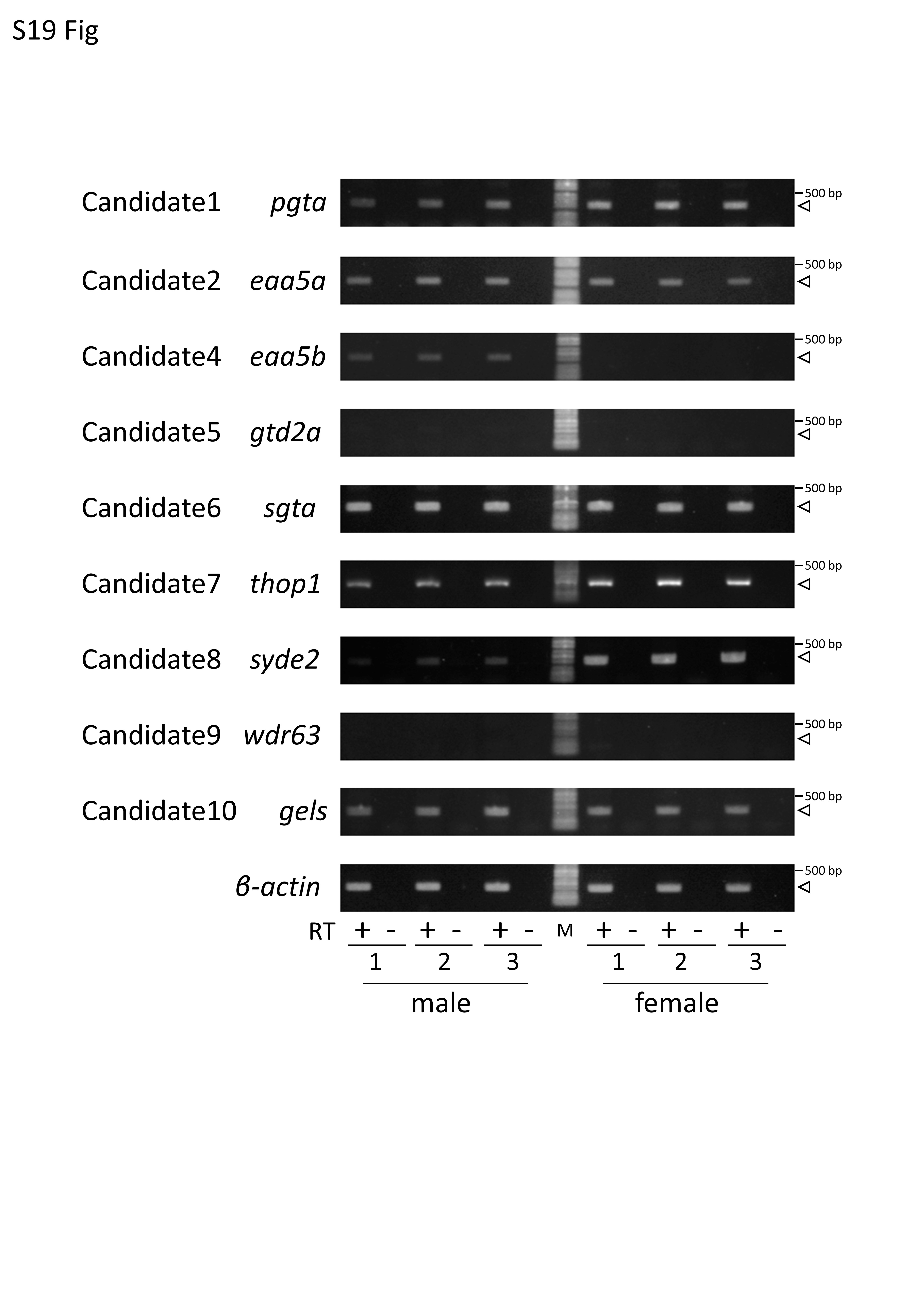

Supplement: S19 Fig — M: size marker. RT+: Reverse transcription reaction with reverse transcriptase. RT-, RT reaction without reverse transcriptase. (JPG) [file pgen.1009705.s019.JPG]

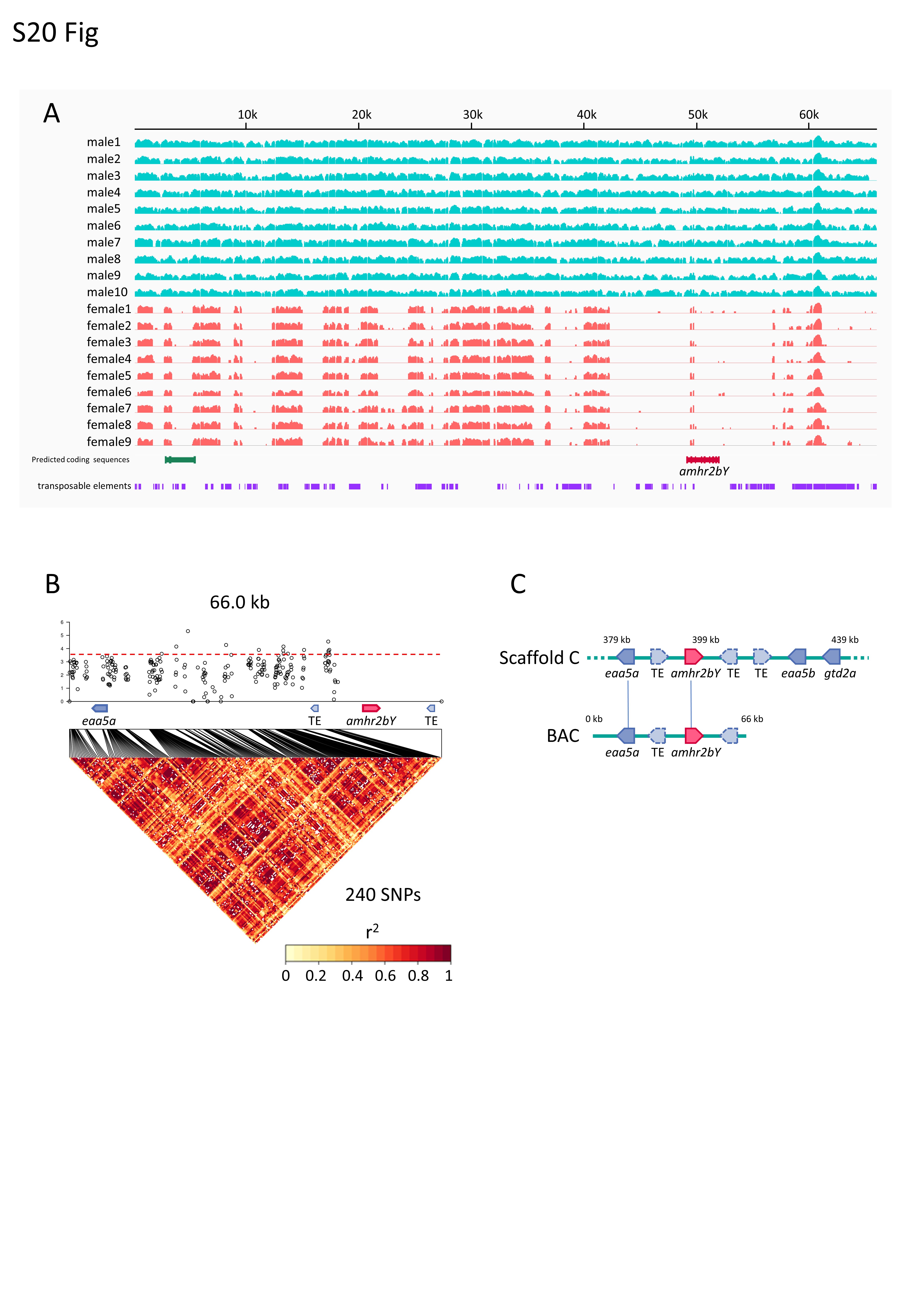

Supplement: S20 Fig — (A) Comparison of mapping coverage of whole-genome resequencing between males and females in BAC clone containing amhr2bY. Y-axis indicates log-scaled sequence depth by whole-genome resequencing analysis; X-axis indicates physical position of the scaffold. Green box indicate predicted coding sequences. Red box indicate Y-linked amhr2bY. Purple boxes indicate transposable elements detected by RepeatMasker. (B) Linkage disequilibrium analysis using BAC clone carrying amhr2bY. Figure shows heat maps of pairwise linkage disequilibrium (r2) between SNPs (lower column) and scaffold-specific high-resolution association test (upper column) using SNPs genotyped by resequencing using ten males and nine females. X-axis indicates physical position of scaffolds. The numbers of SNPs in the figure indicate that the total number of SNPs using calculation for pairwise r2 value and scaffold-specific association test. Red line indicates scaffold-wide significance threshold (Bonferroni-corrected p-value = 0.05). Color of each SNP reflects pairwise r2 value: red indicates higher values, yellow indicates lower values. (C) Comparison for order of predicted genes around amhr2bY between scaffold C and BAC clone carrying amhr2bY. (JPG) [file pgen.1009705.s020.JPG]

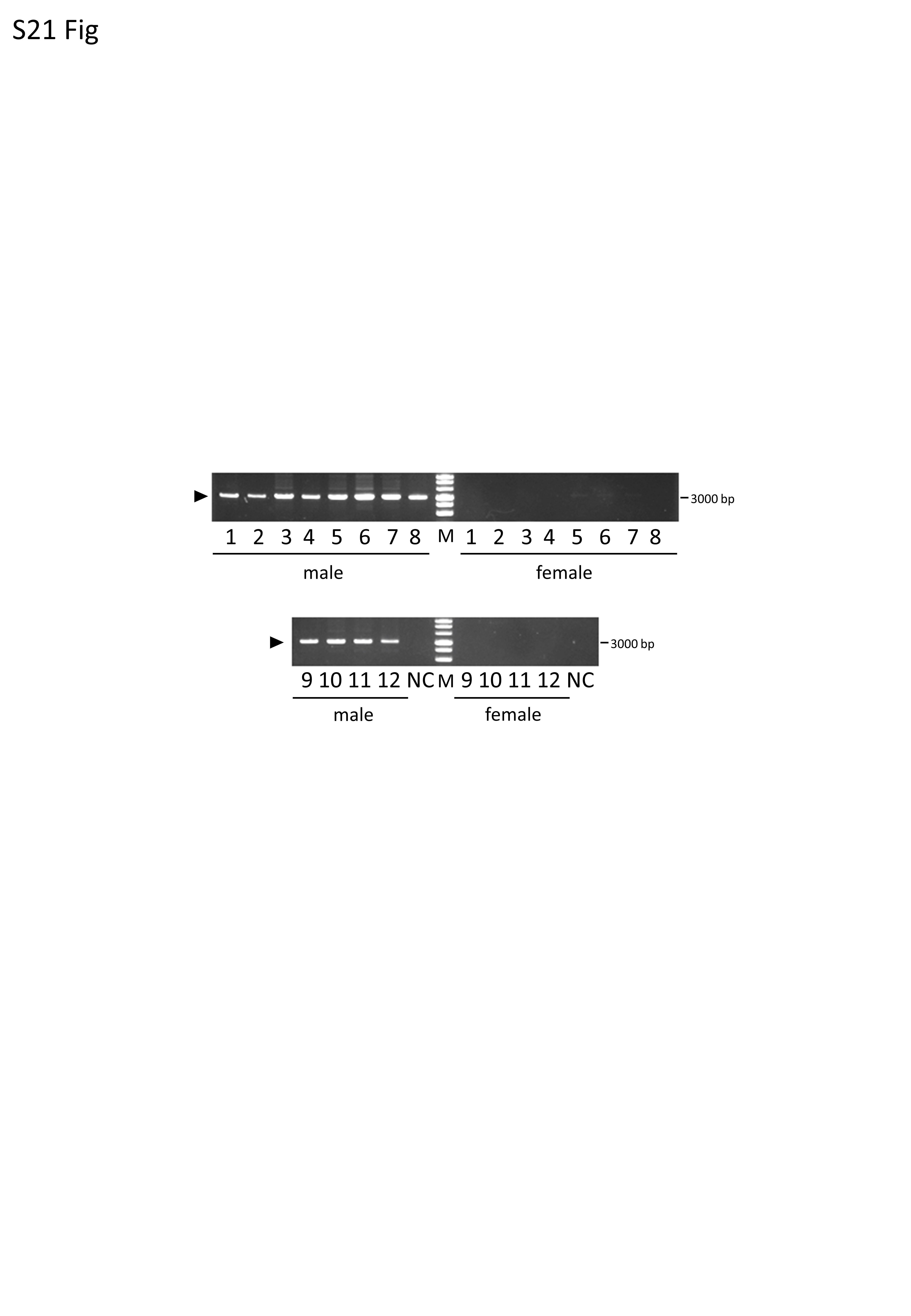

Supplement: S21 Fig — Results of agarose gel electrophoresis using primer set amhr2bY-1F and amhr2bY-2R for 12 males and 12 females from Tama River population. M: size marker, NC: negative control. (JPG) [file pgen.1009705.s021.JPG]

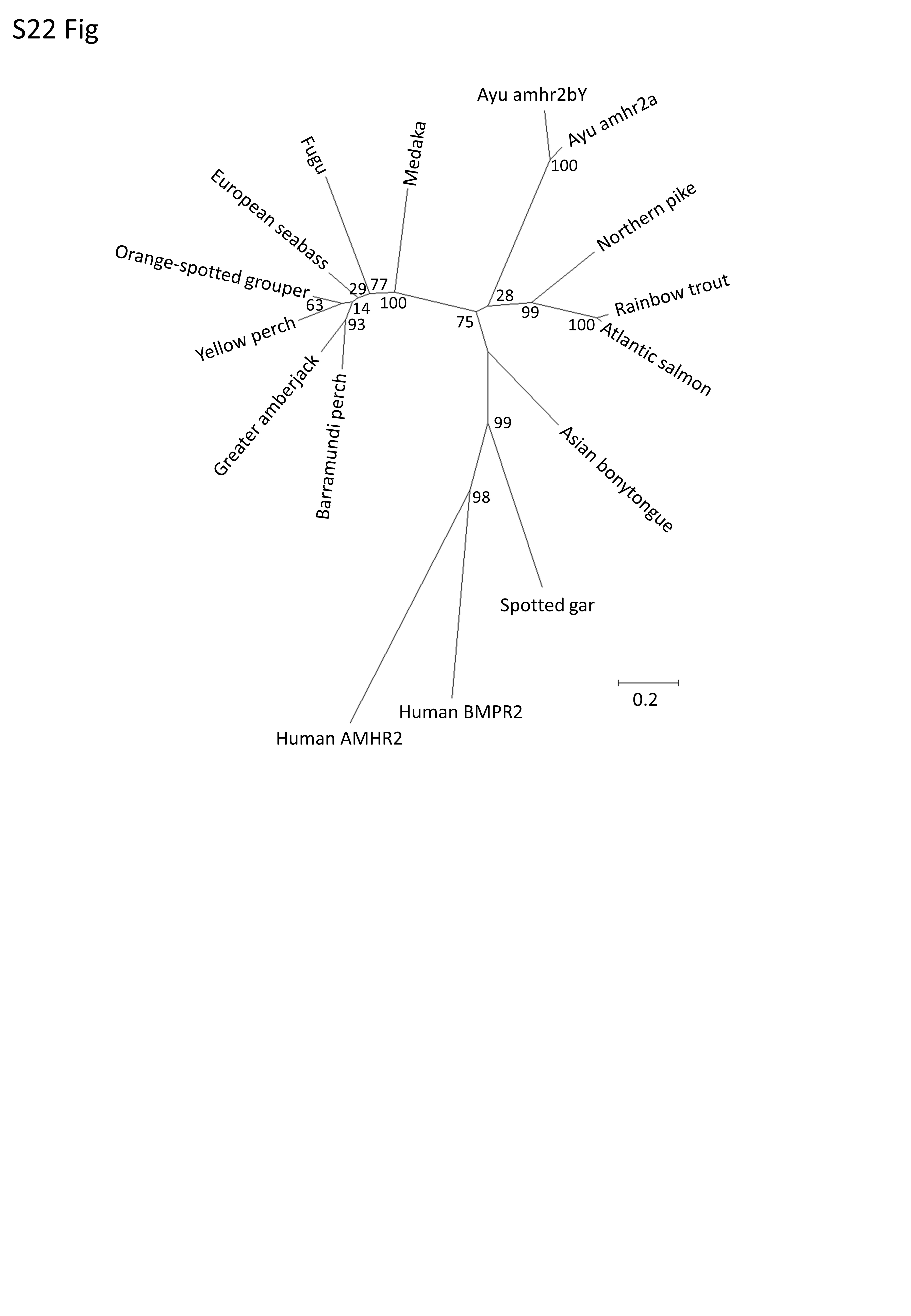

Supplement: S22 Fig — Bootstrap percentages based on 1000 replicates are indicated at nodes. (JPG) [file pgen.1009705.s022.JPG]

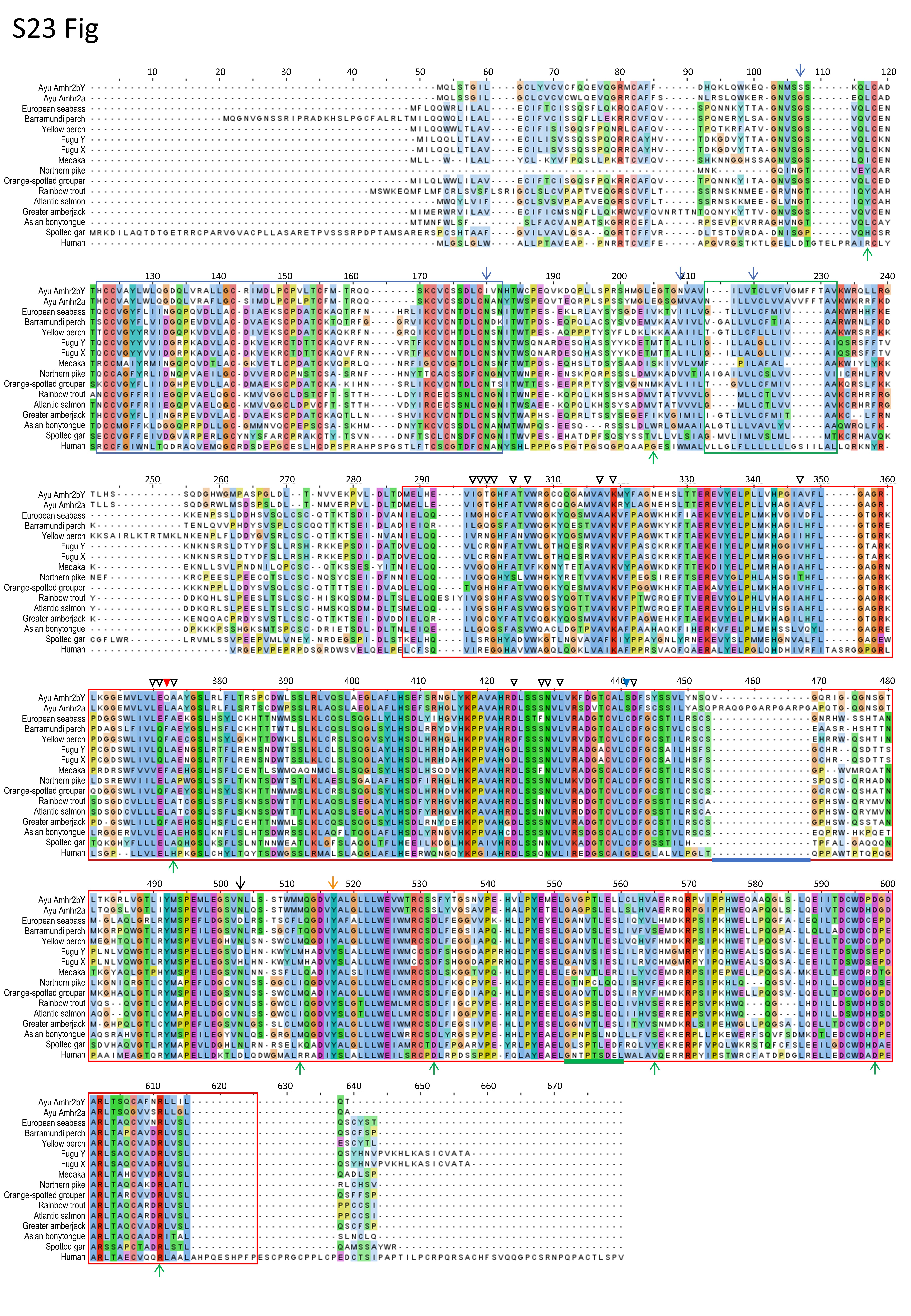

Supplement: S23 Fig — Blue box indicates activin type I and II receptor domain (amino acids 53–104 in Amhr2bY and 54–105 in Amhr2a). Green box indicates transmembrane domain (amino acids 135–151 in Amhr2bY and 136–152 in Amhr2a). Red boxes indicate catalytic domain of the serine/threonine kinases, bone morphogenetic protein, and anti-Müllerian hormone type II receptors (amino acids 208–492 in Amhr2bY and 209–507 in Amhr2a). Arrowheads indicate amino acid residues in evolutionarily conserved ATP binding site in catalytic domain of serine/threonine kinases in BMPR2 and AMHR2 family. Red arrowhead indicates ayu Amhr2bY-specific amino acid change in ATP binding site. Blue arrowhead indicates ayu-specific amino acid change in ATP binding site. Blue arrows indicate Amhr2bY-specific amino acid changes. Green arrows and green line indicate amino acid changes associated with human persistent Müllerian duct syndrome [70]. Orange arrow indicates amino acid residues critical for amhr2 function, as identified in medaka mutant [69]. It was conserved between Amhr2Yb and Amhr2a of ayu and that of other teleosts. Black arrow indicates amino acid residue determining genetic sex of fugu [13]. This amino acid did not alter between Amhr2bY and Amhr2a. Blue line indicates Amhr2a-specific 15-amino acid insertion. (JPG) [file pgen.1009705.s023.JPG]

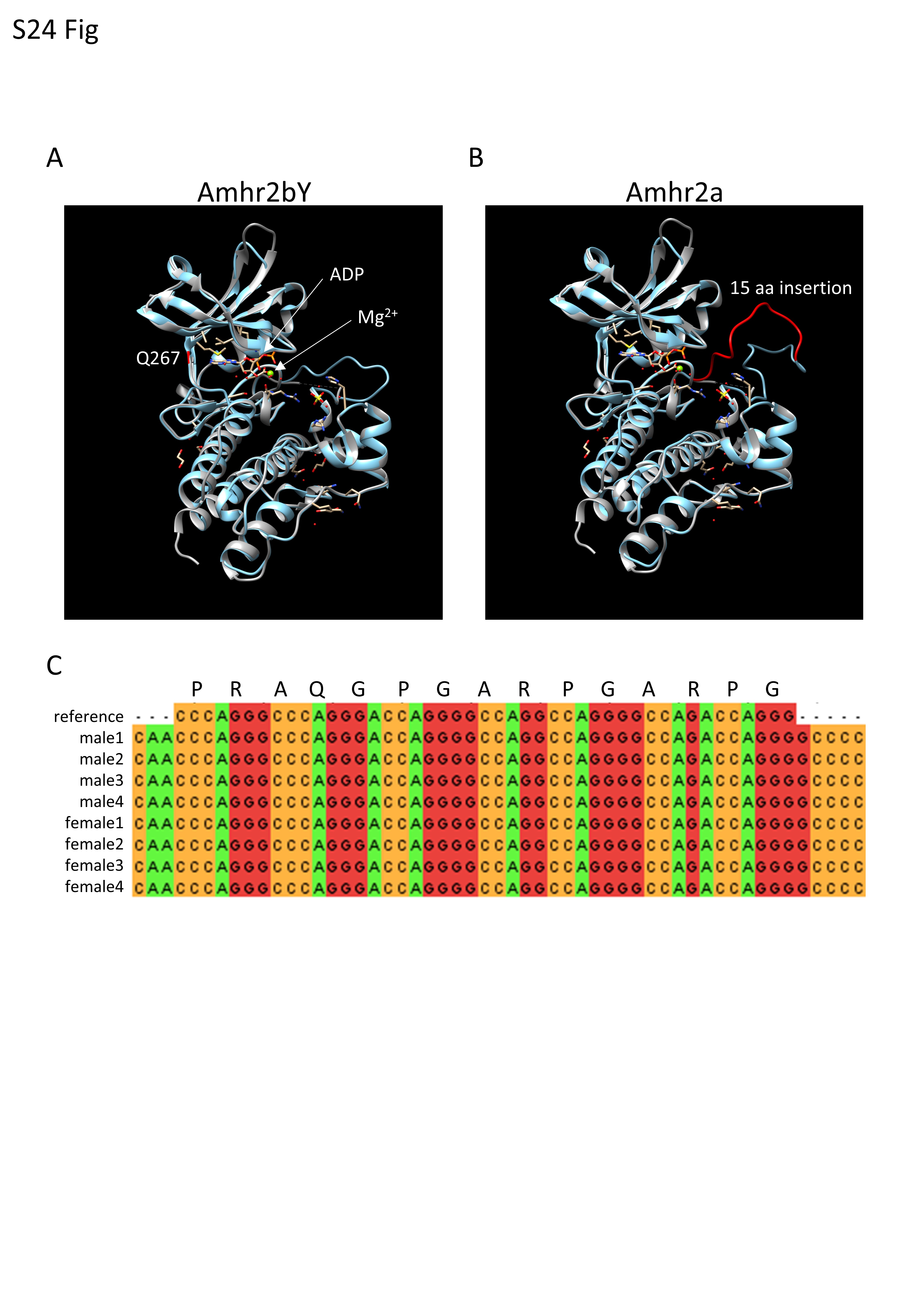

Supplement: S24 Fig — (A) Homology model of Amhr2bY (blue) with human BMPR2 as template (gray) generated using SWISS-MODEL. Red amino acid residues are Q267. (B) Homology model of Amhr2a (blue) with human BMPR2 as template (gray) generated using SWISS-MODEL. Red amino acid residues indicate 15-amino acid insertion. (C) Validation of 15-amino acid insertion in amhr2a by RT-PCR. (JPG) [file pgen.1009705.s024.JPG]

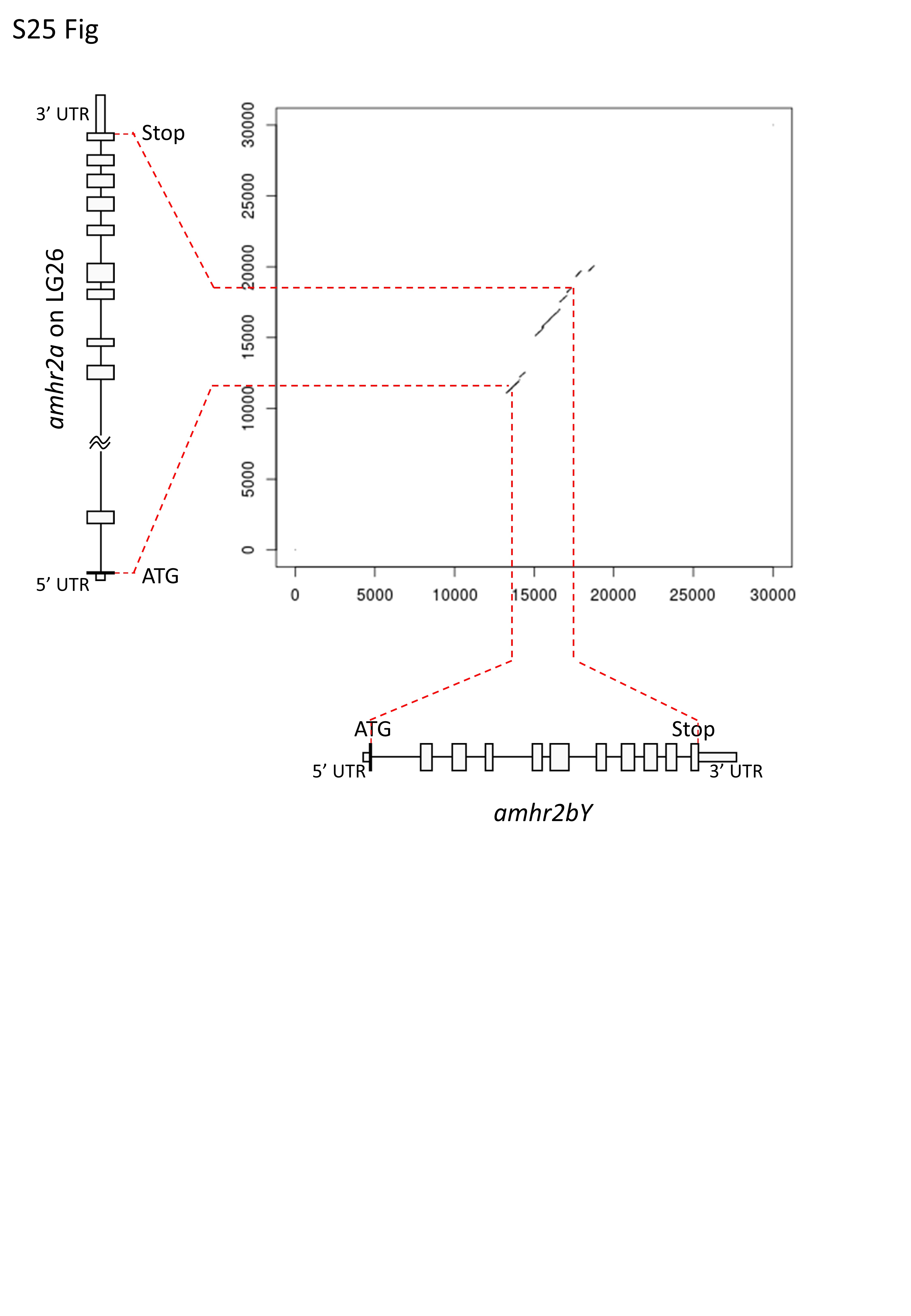

Supplement: S25 Fig — Dot plots of pairwise alignment are shown. Only exon regions of amhr2 showed similarities. (JPG) [file pgen.1009705.s025.JPG]

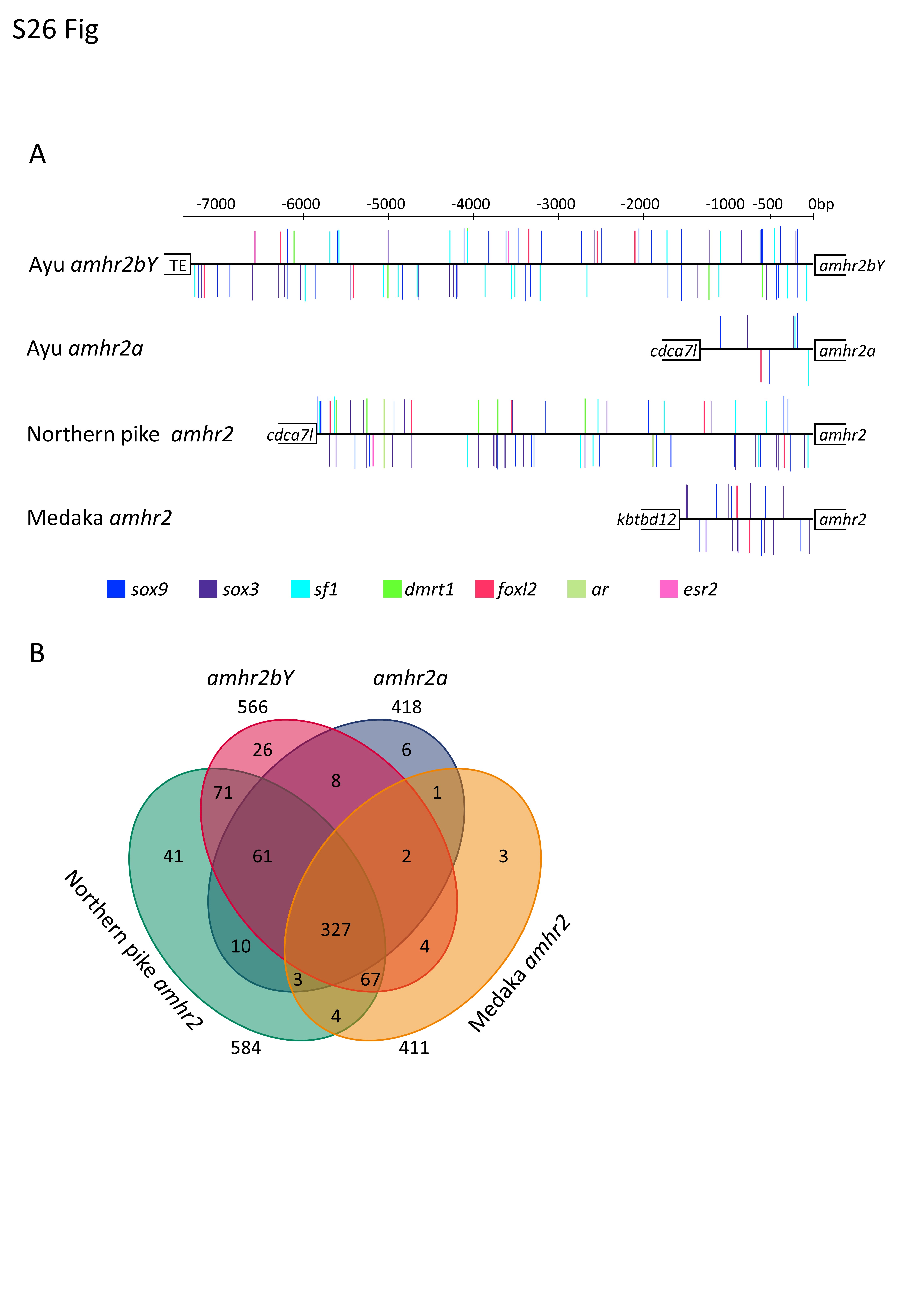

Supplement: S26 Fig — (A) Comparison of predicted transcription factor binding sites for sex differentiation-related genes in intergenic region between amhr2s and adjacent gene in ayu, northern pike and medaka. Multiple binding sites were detected for some sex differentiation-related transcription factors, as follows: 32 sox9 binding sites, 18 sox3 binding sites, 20 nr5a1 (sf1) binding sites, five dmrt1 binding sites, six foxl2 binding sites and two esr2 (estrogen receptor β) binding sites. In the putative promoter region of autosomal amhr2a (1,327 bp), 2,361 binding sites for 418 transcription factors were detected, including three sox9 binding sites, two sox3 binding sites, two nr5a1 binding sites and one foxl2 binding site. (B) Venn diagram showing number of transcription factors with predicted binding sites in putative promoter regions of amhr2bY, amhr2a, northern pike amhr2 and medaka amhr2. In total, 398 transcription factors had predicted binding sites in the promotor regions of both amhr2bY and amhr2a. Comparisons of the regulatory regions of amhr2s among ayu, northern pike and medaka, revealed 26 transcription factors that regulated only amhr2bY. (JPG) [file pgen.1009705.s026.JPG]

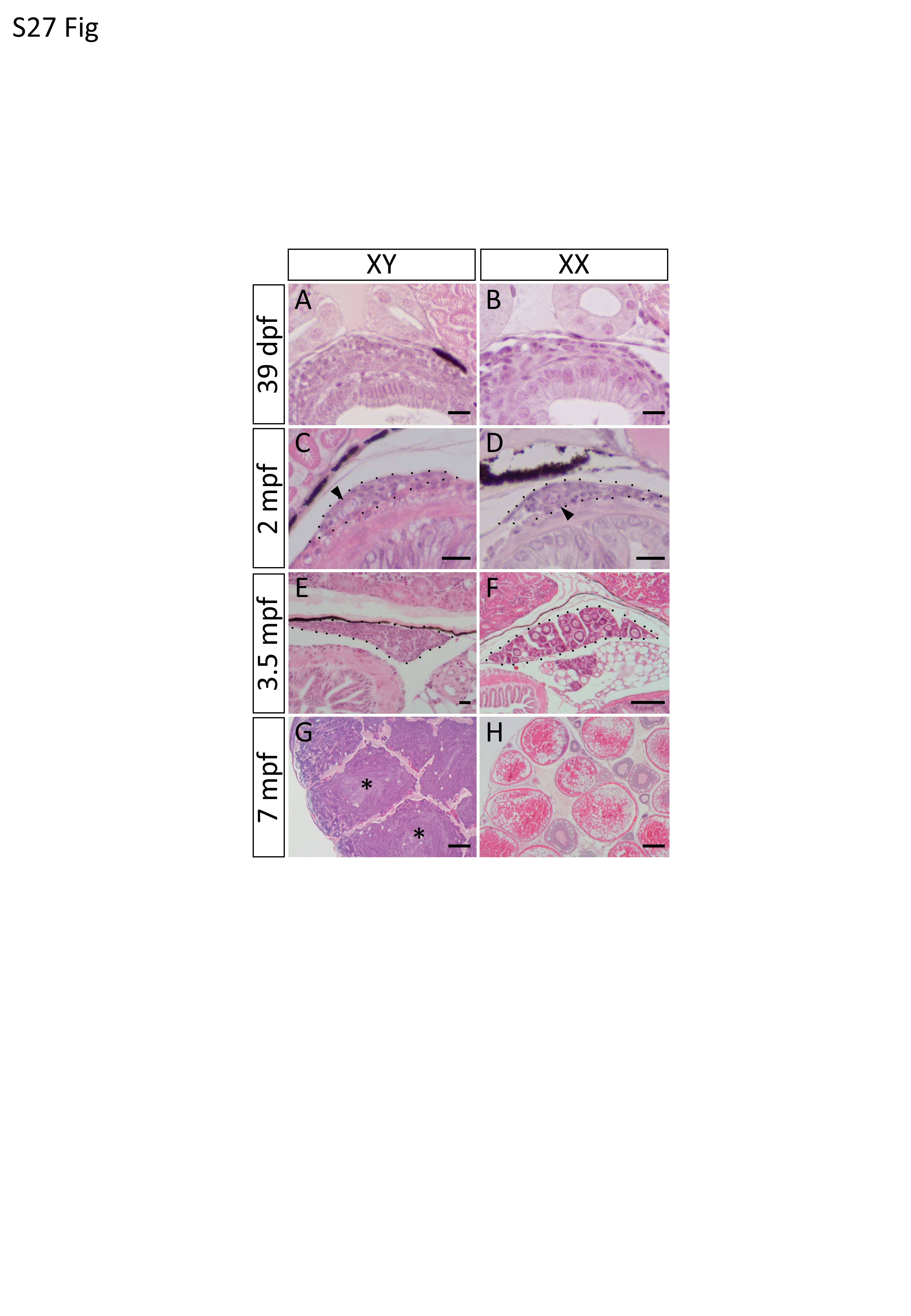

Supplement: S27 Fig — Tissue sections were stained with hematoxylin–eosin. Future gonadal region of ayu larvae at 39 days post fertilization in XY (A) and XX (B). Ayu gonads at 2 months post fertilization (mpf) XY (C), 2 mpf XX (D), 3.5 mpf XY (E), 3.5 mpf XX (F), 7 mpf XY (G), and 7 mpf XX (H). Arrowheads in C and D indicate germ cells. Asterisk in G indicates sperm. Dotted lines indicate gonad outline. Scale bar: 10 μm in A–E and 100 μm in F–H. (JPG) [file pgen.1009705.s027.JPG]

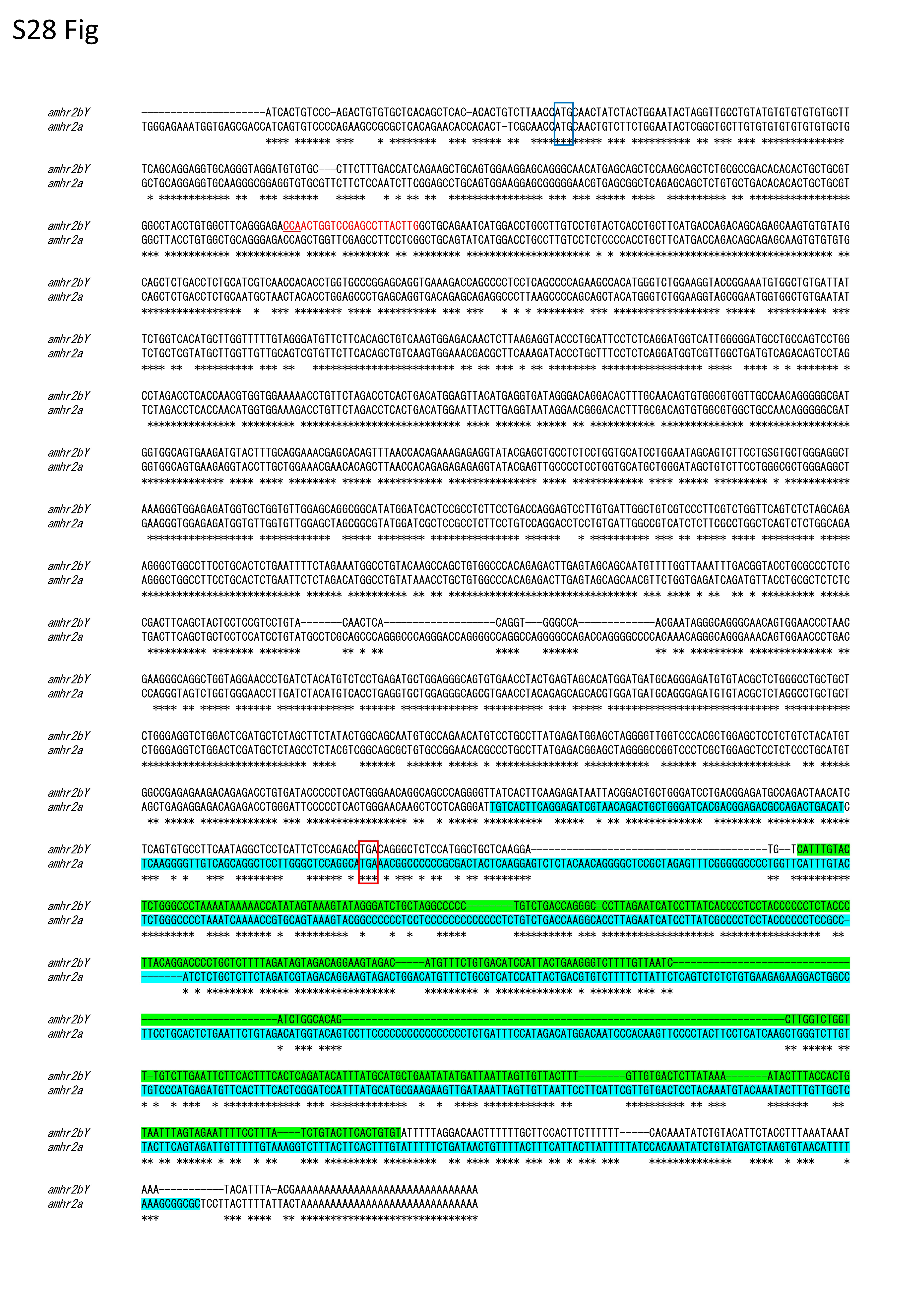

Supplement: S28 Fig — Blue box indicates start codon, red box indicates stop codon. Blue line indicates RNA probe position for 3′ UTR of amhr2bY. Green line indicates RNA probe position for 3′ UTR of autosomal amhr2a. Asterisks indicate identical nucleotides. Red characters indicate the target site for guide RNA of CRISPR/Cas9. Underline indicates PAM sequence. (JPG) [file pgen.1009705.s028.JPG]

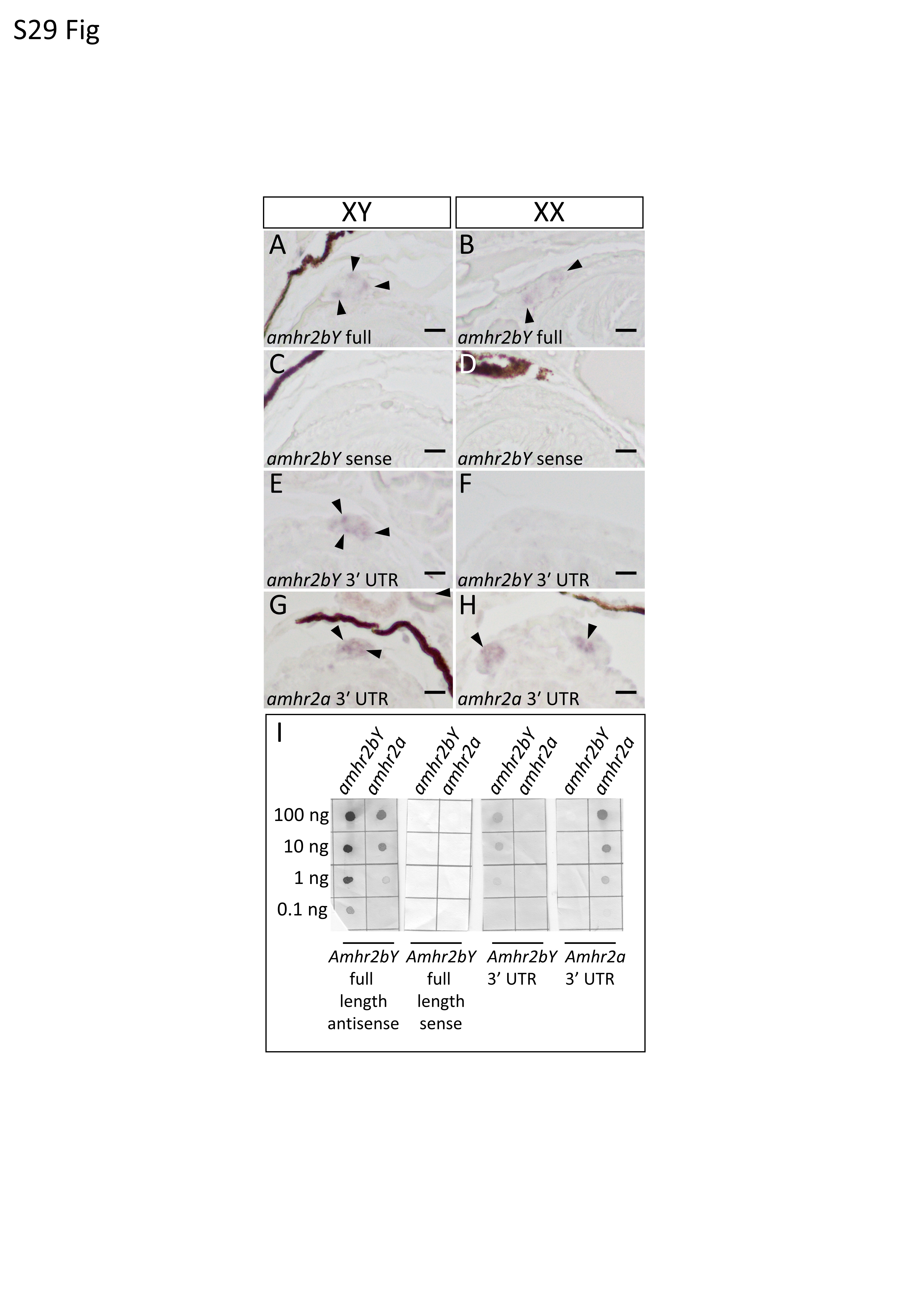

Supplement: S29 Fig — (A–D) In situ hybridization using antisense (A and B) and sense (C and D) probes of amhr2bY-containing coding region at 2 mpf XY (A and C) and 2 mpf XX (B and D). (E and F) In situ hybridization using probe for 3′ UTR of amhr2bY at 2 mpf XY (E) and 2 mpf XX (F). (G and H) In situ hybridization for 3′ UTR of amhr2a at 2 mpf XY (G) and 2 mpf XX (H). amhr2bY mRNA was specifically expressed in somatic cells of genetic males with undifferentiated gonads. Autosomal amhr2a mRNA was detected in both XY and XX undifferentiated gonads. Arrowheads indicate positive signals. Scale bar: 10 μm. (I) Validation of specificity of RNA probes by dot blot hybridization. RNA probe for amhr2bY-containing coding region detected both amhr2bY and autosomal amhr2a RNA, whereas probe derived from 3′ UTR of amhr2bY detected only amhr2bY RNA and not amhr2a RNA. Similarly, probe for 3′ UTR of amhr2a RNA only detected amhr2a. (JPG) [file pgen.1009705.s029.JPG]

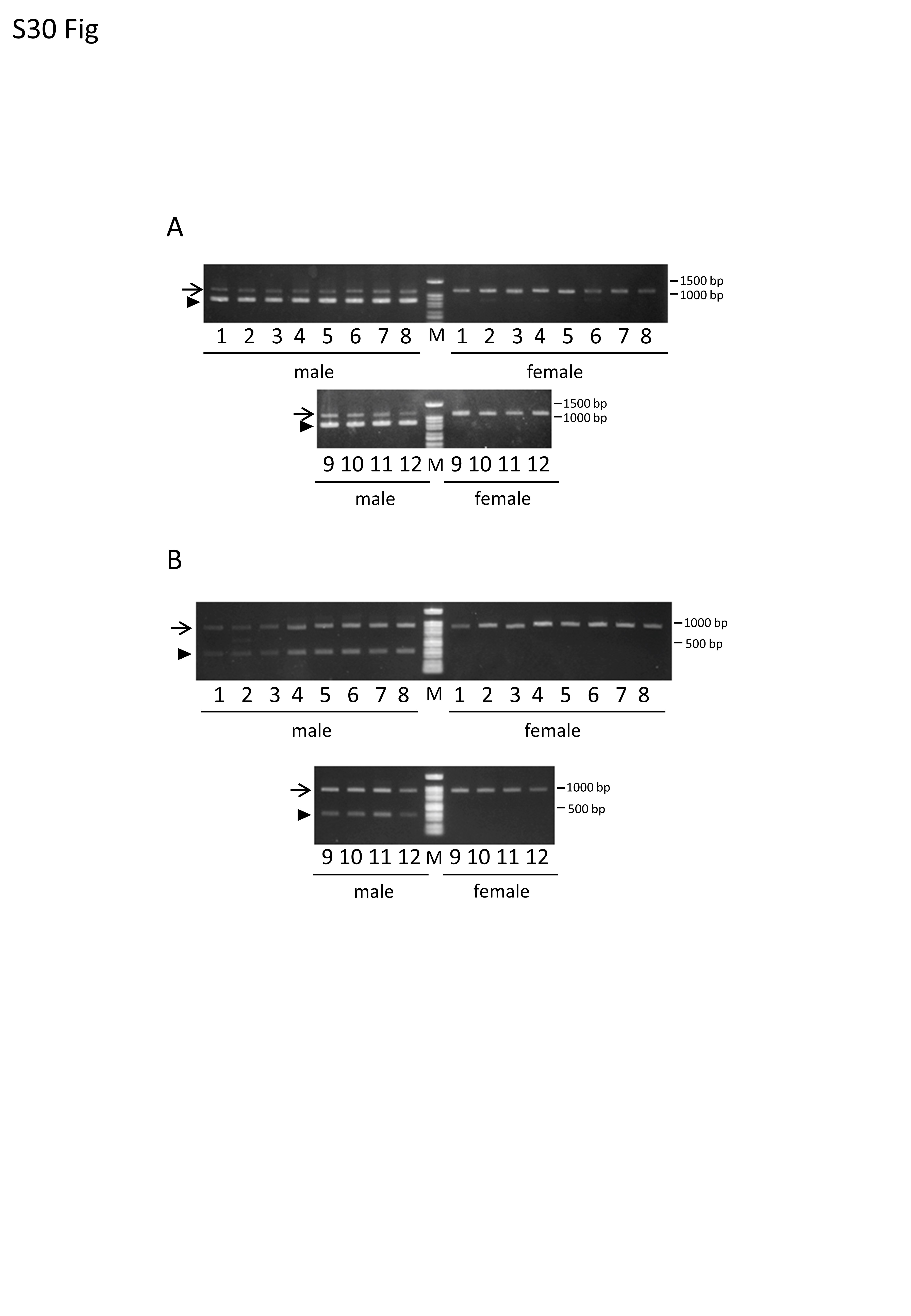

Supplement: S30 Fig — (A) Agarose gel electrophoresis results using primer set Ayu-sex-1F and Ayu-sex-2R for 12 males and 12 females from Tama River population. (B) Agarose gel electrophoresis results using primer set Ayu-sex-3F and Ayu-sex-4R for 12 males and 12 females from Tama River population. Genetic males have two amplified bands derived from amhr2bY (arrowhead) and autosomal amhr2a; genetic females have one amplified band derived from autosomal amhr2b (arrow). M: size marker. (JPG) [file pgen.1009705.s030.JPG]
