# Supplementary material for: A Y-linked anti-Müllerian hormone type-II receptor is the sex-determining gene in ayu, Plecoglossus altivelis
Source: PLoS Genet. 2021 Aug 26;17(8):e1009705. doi: 10.1371/journal.pgen.1009705 (PMC8389408; doi:10.1371/journal.pgen.1009705)
Supplement: S1 Text — (DOCX) [file pgen.1009705.s047.docx]

**Supporting information**

**Supplemental Materials and Methods**

*Screening and sequencing for BAC clones carrying sex determining locus of ayu*

A BAC library was constructed from genomic DNA of sperm cells taken from one male ayu according to Katagiri et al [1]. Genomic DNA was then integrated into BamHI site of pBACe3.6 vector and reactions were transfected to E. coli DH10B strain. BAC library three-dimensional pools were generated according to a PCR-based protocol [2,3]. The PCR screening was carried out Takara Ex Taq (Takara Bio, Shiga, Japan) using the primer set amhr2bY-1F and amhr2bY-2R (S12 Table) under the following cycling conditions: initial denaturation for 2 min at 95°C, followed by 30 cycles of 30 30 s at 95°C, 30 s at 56°C and 30 s at 72°C, with a final extension of 3 min at 72°C. BAC DNA was extracted using a QIAGEN Plasmid Midi Kit (Qiagen) according to the manufacturer’s instructions. Sequencing libraries were constructed by Nextera XT DNA Library Preparation Kit (Illumina, San Diego, CA) and sequenced on Illumina MiSeq system using 250 bp paired-end sequencing. The sequencing data was submitted to the DDBJ Sequence Read Archive (DRA) under accession number DRA011927. Sequence read were *de novo* assembled using the Platanus v1.2.4 genome assembler with default parameters. Then, the assembly contigs were ordered according to the whole genome sequences. The assembly sequences of BAC clone have been deposited in the DDBJ database under the accession number LC632297. For linkage disequilibrium analysis and association mapping by whole-genome resequencing, resequencing reads were mapped to the BAC sequence as reference by bwa mem. Variants were called with GATK HaplotypeCaller and were filtered in VCFtools.

*Validation of sex determining locus detected by genome wide association study using linkage analysis of other mapping families*

Mapping families were generated from artificial fertilization of two pair of ayu captured from a wild population in the Nagaragawa River. The siblings were mixed and reared until the phenotypic sex could be identified. Genomic DNA was extracted from the caudal fins of the ayu (88 siblings (44 males and 44 females) and duplicate of two pair of parents) using a DNeasy Blood and Tissue kit (Qiagen). Sequencing libraries were prepared GRAS-Di protocol [4] and sequenced on the Illumina NovaSeq 6000 platform with 150-bp paired-end reads. Library construction and sequencing were carried out by Eurofins Genomics. Sequencing data have been deposited in the DRA under the accession number DRA011943. Low-quality reads were trimmed by Trimmomatic v3.6. Trimmed reads were mapped to the ayu draft genome sequence by bwa mem v0.7.12. Variant calling was performed in GATK HaplotypeCaller v4.0.5 according to GATK best practice recommendations. Raw variants were filtered with VCFtools v0.1.16 (parameters: --minQ 100 --minDP 10 --maxDP 255 –min-meanDP 30 --max-missing 0.95 --remove-indels --maf 0.01 --hwe 0.0001 --thin 500). Parentage assignment using filtered SNP was performed by AlphaAssign software [5]. Genetic linkage maps using male parent informative markers were constructed with the pseudo-testcross strategy using Lep-Map3 with LOD threshold of 10. Linkage analysis of genetic sex by simple interval mapping with 1 cM intervals was performed using the R/qtl software package [6]. The LOD threshold for genome-wide significance was obtained based on permutation test (10000 permutations, P = 0.001). The sequences flanking all SNPs mapped to linkage maps of the Nagaragawa River families were listed in S15 and S16 Tables.

*Histological analysis of ayu gonad development*

Ayu larvae at 39 days post fertilization and 2 and 3.5 months post fertilization (mpf) and gonads at 7 mpf were fixed in Bouin’s solution. Genomic sex was identified by genomic PCR using the primer set Ayu-sex-1F and Ayu-sex-2R (S12 Table). Tissues were dehydrated and processed using standard procedures and then embedded in Paraplast Plus tissue embedding medium (McCormick Scientific, St. Louis, MO, USA). Sections of 5-µm thickness were serially cut and stained with hematoxylin–eosin according to standard protocols. At least three specimens were prepared for each time point and genetic sex.

*Dot blot hybridization for* amhr2

The cDNAs of *amhr2Y* and autosomal *amhr2* were obtained by RT-PCR using the primer sets amhr2Y-7F/amhr2Y-8R and amhr2-5F/amhr2-6R, respectively (S12 Table), and the amplified products were each cloned into the pGEM-T easy vector (Promega, Fitchburg, WI, USA). The sense RNA for *amhr2Y* and autosomal *amhr2* was synthesized by *in vitro* transcription with the ScriptMAX Thermo T7 Transcription kit (TOYOBO Co., Ltd, Osaka, Japan). The RNA was blotted onto a Hybond-N+ nylon membrane (GE Healthcare Bio-Sciences, Piscataway, NJ, USA) and fixed to the membrane by heating for 2 h at 80 °C. Sense and antisense digoxigenin (DIG)-labeled cRNA probes for full-length cDNA for *amhr2Y*, the 3’ untranslated region (UTR) of *amhr2Y*, and the 3’ UTR of autosomal *amhr2* were generated by *in vitro* transcription with the DIG RNA labeling kit (Roche Diagnostics, Mannheim, Germany). The template DNA for the 3’ UTR was obtained by RT-PCR using the primer sets amhr2Y-9F/amhr2Y-10R and amhr2-7F/amhr2-8R (S12 Table). The membranes were then hybridized with sense or antisense cRNA probes at 60 °C for 24 h. Hybridization signals were detected using alkaline phosphatase–conjugated anti-DIG antibody (Roche Diagnostics, 1/3,000 dilution) and NBT/BCIP chromogenic substrates (Roche Diagnostics).

**References**

**1.** Katagiri T, Asakawa S, Minagawa S, Shimizu N, Hirono I, Aoki T: Construction and characterization of BAC libraries for three fish species; rainbow trout, carp and tilapia. Anim Genet. 2001;32: 200-204. DOI: 10.1266/ggs.78.103

**2.** Bruno WJ, Knill E, Balding DJ, Bruce DC, Doggett NA, Sawhill WW, et al. Efficient pooling designs for library screening. Genomics. 1995; 26: 21-30. DOI: 10.1016/0888-7543(95)80078-z

**3.** Bouzidi MF, Franchel J, Tao Q, Stormo K, Mraz A, Nicolas P, et al. Sunflower BAC library suitable for PCR screening and physical mapping of targeted genomic regions. Theor Appl Gene. 2006;113: 81-89. DOI: 10.1007/s00122-006-0274-6

**4.** Hosoya S, Hirase S, Kikuchi K, Nanjo K, Nakamura Y, Kohno H, et al. Random PCR-based genotyping by sequencing technology GRAS-Di (genotyping by random amplicon sequencing, direct) reveals genetic structure of mangrove fishes. Mol Ecol Resour. 2019; 19: 1153-1163. DOI: 10.1111/1755-0998.13025

**5.** Whalen A, Gorjanc G, Hickey JM. Parentage assignment with genotyping-by-sequencing data. J Anim Breed Genet. 2019;136: 102-112. DOI: 10.1111/jbg.12370

**6.** Broman KW, Wu H, Sen S, Churchill GA. R/qtl: QTL mapping in experimental crosses. Bioinformatics. 2003;19: 889-890. DOI: 10.1093/bioinformatics/btg112
